# Supplementary material for: Trimester-Specific Serum Lipid Profiles in Gestational Diabetes Mellitus: A Systematic Review, Meta-Analysis, and Meta-Regression
Source: Medicina (Kaunas). 2025 Jul 17;61(7):1290. doi: 10.3390/medicina61071290 (PMC12300116; doi:10.3390/medicina61071290)

Study	Experimental Total	Experimental Mean	Experimental SD	Control Total	Control Mean	Control SD	SMD	95%−CI	Weight (fixed)	Weight (random)
Montelongo, 1992	9	1.54	0.6600	12	1.17	0.3500	0.70	[−0.19; 1.60]	0.0%	0.2%
Shelley−Jones, 1993	16	2.88	0.9200	15	2.03	0.6600	1.03	[0.27; 1.78]	0.0%	0.3%
Shelley−Jones, 1993	19	2.49	0.7200	15	2.03	0.6600	0.65	[−0.05; 1.34]	0.1%	0.3%
Nolan C, 1995	38	1.36	0.2100	350	1.62	1.0000	−0.27	[−0.61; 0.06]	0.2%	0.4%
Clark C, 1997	52	2.21	1.1000	127	1.93	0.7200	0.33	[0.00; 0.65]	0.2%	0.4%
Seghieri G, 2003	15	2.80	1.3000	78	2.40	0.8000	0.44	[−0.11; 1.00]	0.1%	0.3%
Toescu V, 2004	12	2.60	0.4000	17	1.70	0.4000	2.19	[1.23; 3.14]	0.0%	0.2%
Tarim E, 2004	28	2.81	0.4800	210	2.03	0.4500	1.71	[1.29; 2.14]	0.1%	0.4%
Di Cianni G, 2005	36	2.47	0.7700	121	1.99	0.6400	0.71	[0.33; 1.09]	0.2%	0.4%
Tarim E, 2006	30	2.54	0.7400	40	2.14	0.5400	0.62	[0.14; 1.11]	0.1%	0.3%
Qui C, 2007	105	2.51	0.9200	96	2.00	0.6900	0.62	[0.34; 0.90]	0.3%	0.4%
Altinova A, 2007	34	2.39	0.1300	31	2.21	0.8900	0.29	[−0.20; 0.78]	0.1%	0.3%
Sánchez−Vera I, 2007	62	1.60	1.6400	45	1.20	0.4400	0.31	[−0.08; 0.70]	0.2%	0.4%
Molnar J, 2008	17	2.00	0.6400	20	1.90	0.7600	0.14	[−0.51; 0.79]	0.1%	0.3%
Davari−Tanha F, 2008	40	2.93	0.5000	40	2.45	1.0700	0.57	[0.12; 1.02]	0.1%	0.4%
Idzior−Walus B, 2008	44	2.70	0.9000	17	1.90	0.3700	1.00	[0.41; 1.59]	0.1%	0.3%
Rizzo M, 2008	27	2.40	0.7000	23	2.20	0.8000	0.26	[−0.30; 0.82]	0.1%	0.3%
McGrowder D, 2009	84	1.83	0.9000	90	1.43	1.9000	0.26	[−0.03; 0.56]	0.3%	0.4%
Vijayalaxmi KD, 2009	25	2.08	0.5000	182	1.41	0.3300	1.88	[1.43; 2.34]	0.1%	0.3%
Kuzmicki M, 2009	81	2.40	0.9000	82	2.25	0.7400	0.18	[−0.13; 0.49]	0.3%	0.4%
Habib F, 2009	100	1.79	0.6900	100	1.44	0.3400	0.64	[0.36; 0.93]	0.3%	0.4%
Bo S, 2009	500	1.70	1.2000	500	1.40	0.8000	0.29	[0.17; 0.42]	1.6%	0.4%
Su Y, 2010	63	2.90	1.0000	58	2.60	0.9600	0.30	[−0.05; 0.66]	0.2%	0.4%
Stein S, 2010	40	2.20	1.3000	80	2.10	1.4000	0.07	[−0.31; 0.45]	0.2%	0.4%
Coskun A, 2010	21	2.28	0.5300	24	1.96	0.6900	0.51	[−0.09; 1.10]	0.1%	0.3%
Santos I, 2010	150	2.50	0.8000	600	1.40	0.6000	1.70	[1.51; 1.90]	0.6%	0.4%
Paradisi G, 2010	12	2.25	0.9700	38	1.84	1.1100	0.37	[−0.28; 1.03]	0.1%	0.3%
Schaefer−Graf U, 2011	150	2.92	0.9800	190	2.84	1.1000	0.08	[−0.14; 0.29]	0.5%	0.4%
Gomathi KG, 2011	26	1.20	0.1300	36	1.01	0.7200	0.34	[−0.17; 0.85]	0.1%	0.3%
Caglar G, 2011	19	2.34	0.7500	15	2.15	0.6800	0.26	[−0.42; 0.94]	0.1%	0.3%
Ozuguz U, 2011	61	2.67	1.0800	40	2.14	0.9300	0.51	[0.11; 0.92]	0.1%	0.4%
Zhaoxia L, 2012	28	5.90	1.9000	32	4.10	1.2000	1.14	[0.59; 1.68]	0.1%	0.3%
Winhofer Y, 2010	26	2.24	0.7900	52	2.06	0.7200	0.24	[−0.23; 0.71]	0.1%	0.3%
Ping F, 2012	488	2.73	1.0600	582	2.38	0.8400	0.37	[0.25; 0.49]	1.7%	0.4%
Vural M, 2012	39	2.63	1.0300	40	2.31	0.8800	0.33	[−0.11; 0.78]	0.1%	0.4%
Naf S, 2012	77	1.99	0.6800	130	1.88	0.5600	0.18	[−0.10; 0.46]	0.3%	0.4%
Baykus Y, 2012	20	2.60	1.0500	20	2.15	0.9400	0.44	[−0.19; 1.07]	0.1%	0.3%
Alanbay I, 2012	37	2.86	0.8000	42	2.12	0.7600	0.94	[0.47; 1.41]	0.1%	0.3%
Rezvan N, 2011	35	2.59	1.0500	35	2.06	1.0500	0.50	[0.02; 0.98]	0.1%	0.3%
Khan R,2012	103	2.14	0.1300	97	1.94	0.2400	1.04	[0.75; 1.34]	0.3%	0.4%
Gkiomisi A, 2013	44	3.03	1.8600	44	2.32	0.9300	0.48	[0.05; 0.90]	0.1%	0.4%
Atay A, 2013	65	2.31	0.3900	66	1.69	0.6600	1.13	[0.77; 1.50]	0.2%	0.4%
Park S, 2013	117	2.61	1.0400	136	2.05	0.6900	0.64	[0.39; 0.90]	0.4%	0.4%
dos Santos−Weiss I, 2012	288	2.20	0.6700	288	1.70	0.5900	0.79	[0.62; 0.96]	0.9%	0.4%
Barden, A 2013	53	3.00	1.0000	72	2.40	0.8000	0.67	[0.31; 1.03]	0.2%	0.4%
Todoric J, 2013	64	2.20	0.7200	165	1.95	0.9000	0.29	[0.00; 0.58]	0.3%	0.4%
Wang D, 2013	30	2.07	0.5300	60	1.93	0.6200	0.23	[−0.21; 0.67]	0.1%	0.4%
Kuzmicki M, 2014	130	2.20	0.8100	140	1.00	0.5900	1.70	[1.42; 1.98]	0.3%	0.4%
Atay A, 2014	68	1.60	0.1800	73	1.52	0.1500	0.48	[0.15; 0.82]	0.2%	0.4%
Javadian P, 2013	52	3.10	1.6100	50	2.16	1.0300	0.69	[0.29; 1.09]	0.2%	0.4%
Ebert T, 2014	74	2.14	1.3100	74	2.02	1.4300	0.09	[−0.24; 0.41]	0.2%	0.4%
Liang Zhaoxia, 2014	35	2.40	0.4300	35	2.10	0.2400	0.85	[0.36; 1.34]	0.1%	0.3%
Bullon, 2013	26	1.92	0.7500	162	1.71	0.8200	0.26	[−0.16; 0.67]	0.1%	0.4%
Guimarães, 2014	150	2.55	0.8300	295	1.38	0.6500	1.63	[1.41; 1.86]	0.5%	0.4%
Houde, 2014	27	1.88	0.7200	99	1.72	0.6400	0.24	[−0.18; 0.67]	0.1%	0.4%
Atay, 2013	37	2.31	0.3900	38	1.69	0.6700	1.12	[0.63; 1.60]	0.1%	0.3%
Wei, 2014	76	3.88	0.8000	86	2.67	0.5800	1.74	[1.38; 2.10]	0.2%	0.4%
Wei, 2014	37	5.50	1.0000	26	2.90	1.1000	2.46	[1.79; 3.13]	0.1%	0.3%
Reyes Lopez	90	2.49	0.7800	108	1.45	0.8700	1.25	[0.94; 1.55]	0.3%	0.4%
Saucedo R, 2014	60	3.24	0.3300	60	2.59	0.3000	2.05	[1.60; 2.49]	0.1%	0.4%
Hesham, 2015	112	2.09	0.6300	218	1.20	0.7400	1.26	[1.01; 1.51]	0.4%	0.4%
Beigi, 2015	40	2.70	0.9600	40	2.65	0.8500	0.05	[−0.38; 0.49]	0.1%	0.4%
Trebotic, 2015	21	2.90	0.8000	19	2.10	0.8000	0.98	[0.32; 1.64]	0.1%	0.3%
Telejko, 2015	49	2.20	0.8100	30	2.20	0.9600	0.00	[−0.45; 0.45]	0.1%	0.3%
Lehmann, 2015	9	2.60	0.8000	15	1.80	0.1100	1.58	[0.62; 2.53]	0.0%	0.2%
Simon Muela, 2015	66	1.95	0.5900	71	1.82	0.5000	0.24	[−0.10; 0.57]	0.2%	0.4%
Altinova A, 2015	30	2.28	1.0400	35	2.41	0.3100	−0.17	[−0.66; 0.32]	0.1%	0.3%
De Melo SF, 2015	200	2.53	0.8500	200	1.14	0.4600	2.03	[1.79; 2.27]	0.4%	0.4%
Takhshid MA, 2015	70	3.02	1.3800	70	3.03	1.1400	−0.01	[−0.34; 0.32]	0.2%	0.4%
Takhshid MA, 2015	65	2.91	0.9300	70	2.98	1.0300	−0.07	[−0.41; 0.27]	0.2%	0.4%
Wurst U, 2015	74	2.14	1.3100	74	2.07	1.4300	0.05	[−0.27; 0.37]	0.2%	0.4%
Li XM, 2015	16	3.97	1.0400	15	2.70	0.8900	1.27	[0.49; 2.06]	0.0%	0.2%
Li XM, 2015	16	3.53	0.8400	15	3.14	1.3600	0.34	[−0.37; 1.05]	0.0%	0.3%
Li XM, 2015	16	2.85	0.4400	15	2.93	0.6200	−0.15	[−0.85; 0.56]	0.0%	0.3%
Korkmazer E, 2015	39	2.50	0.9100	40	1.83	0.5300	0.89	[0.43; 1.36]	0.1%	0.3%
Jia X, 2014	86	2.22	0.9400	92	2.41	0.9400	−0.20	[−0.50; 0.09]	0.3%	0.4%
Iyidir OT, 2014	26	2.81	1.0600	24	5.27	1.8700	−1.61	[−2.26; −0.97]	0.1%	0.3%
Demirpençe M, 2016	20	2.04	0.7000	11	2.50	0.8100	−0.61	[−1.36; 0.15]	0.0%	0.3%
Zhang Y, 2016	40	2.91	1.5000	240	1.77	0.5500	1.50	[1.14; 1.86]	0.2%	0.4%
Liang ZX, 2015	55	5.80	1.4000	50	2.70	0.7000	2.74	[2.20; 3.28]	0.1%	0.3%
Edu A, 2016	13	2.11	0.7200	96	2.00	0.6800	0.16	[−0.42; 0.74]	0.1%	0.3%
Ertug EY, 2016	29	2.48	0.8800	20	1.81	0.5500	0.86	[0.27; 1.46]	0.1%	0.3%
Mou Y, 2016	40	2.03	0.7800	40	0.95	0.5000	1.63	[1.12; 2.14]	0.1%	0.3%
Zheng D, 2016	50	1.46	0.3800	50	0.63	0.2100	2.68	[2.14; 3.23]	0.1%	0.3%
Khosrowbeygi A, 2015	30	3.15	0.2600	30	2.53	0.2100	2.59	[1.89; 3.29]	0.1%	0.3%
Aydemir B, 2015	116	2.46	0.6600	120	2.26	0.3400	0.38	[0.12; 0.64]	0.4%	0.4%
Qiu YH, 2016	223	2.88	1.2300	265	3.05	1.3300	−0.13	[−0.31; 0.05]	0.8%	0.4%
Ye D, 2016	556	2.50	0.9000	500	2.10	0.8000	0.47	[0.35; 0.59]	1.6%	0.4%
Mac−Marcjanek K, 2017	104	2.40	0.7600	41	2.43	0.7600	−0.04	[−0.40; 0.32]	0.2%	0.4%
Tu WJ, 2017	135	1.32	0.3500	1015	1.23	0.3200	0.28	[0.10; 0.46]	0.8%	0.4%
Zhou X, 2017	180	3.63	0.3700	60	3.66	0.4500	−0.08	[−0.37; 0.22]	0.3%	0.4%
del Mar Roca−Rodríguez M, 2017	63	11.80	4.2000	63	8.47	2.4600	0.96	[0.59; 1.33]	0.2%	0.4%
Usluogullari B, 2017	48	1.53	0.5500	46	1.70	0.7600	−0.26	[−0.66; 0.15]	0.1%	0.4%
Saucedo R, 2017	80	3.24	1.1000	80	2.49	1.0100	0.71	[0.39; 1.03]	0.2%	0.4%
Li L, 2017	200	3.37	1.3300	410	2.76	1.2200	0.48	[0.31; 0.66]	0.8%	0.4%
Mousavi SN, 2017	200	2.52	0.9300	200	2.00	0.3100	0.75	[0.55; 0.95]	0.6%	0.4%
Ersoy GS, 2016	62	2.60	1.1600	73	2.02	0.7600	0.60	[0.25; 0.94]	0.2%	0.4%
Jameshorani M, 2018	65	12.21	4.5200	65	9.83	1.5400	0.70	[0.35; 1.06]	0.2%	0.4%
Yang Y, 2016	209	3.68	1.9300	215	2.86	0.8000	0.56	[0.36; 0.75]	0.7%	0.4%
Barat S, 2018	250	3.12	0.7800	87	2.32	0.8200	1.01	[0.75; 1.27]	0.4%	0.4%
Yuan X, 2018	86	2.85	1.1000	273	2.29	0.7200	0.68	[0.43; 0.92]	0.4%	0.4%
Eken MK, 2018	63	2.70	0.9000	64	2.20	0.8000	0.58	[0.23; 0.94]	0.2%	0.4%
Bukowiecka−Matusiak M, 2018	32	2.40	0.6700	11	1.80	0.7400	0.86	[0.15; 1.57]	0.0%	0.3%
Zhang L, 2018	77	1.56	0.5100	154	1.31	0.5400	0.47	[0.19; 0.75]	0.3%	0.4%
Bagci H, 2018	40	2.55	0.8600	40	2.29	0.7400	0.32	[−0.12; 0.76]	0.1%	0.4%
Khosrowbeygi A, 2018	40	2.89	1.3900	40	2.51	1.0100	0.31	[−0.13; 0.75]	0.1%	0.4%
Franzago M, 2018	104	2.64	0.8200	124	2.54	0.9500	0.11	[−0.15; 0.37]	0.4%	0.4%
Al−Ajlan A, 2018	116	1.50	0.6000	303	1.30	0.6000	0.33	[0.12; 0.55]	0.5%	0.4%
Al−Daghri NM, 2018	63	1.40	0.6000	54	1.20	0.6000	0.33	[−0.03; 0.70]	0.2%	0.4%
Bao W, 2018	107	2.56	1.4500	214	2.10	1.3200	0.34	[0.10; 0.57]	0.4%	0.4%
Siddiqui K, 2017	14	2.57	0.8300	21	2.45	0.9600	0.13	[−0.55; 0.81]	0.1%	0.3%
Cao W, 2018	33	3.05	0.5000	33	2.15	0.3000	2.16	[1.54; 2.77]	0.1%	0.3%
Yue CY, 2018	88	4.49	1.5000	456	3.75	1.9200	0.40	[0.17; 0.63]	0.5%	0.4%
Tuzun D, 2018	54	2.27	0.6900	33	2.07	0.5700	0.31	[−0.13; 0.74]	0.1%	0.4%
Siddiqui K, 2019	44	2.36	0.6800	48	1.71	0.4900	1.10	[0.66; 1.54]	0.1%	0.4%
Demi E, 2019	85	2.36	0.7900	90	2.21	0.8800	0.18	[−0.12; 0.48]	0.3%	0.4%
Li S, 2018	90	2.69	0.9000	90	2.27	0.9100	0.46	[0.17; 0.76]	0.3%	0.4%
de la Torre NG, 2019	130	1.77	0.5900	802	1.90	0.6500	−0.20	[−0.39; −0.02]	0.7%	0.4%
Pezeshki B, 2019	30	1.16	0.3700	301	1.07	0.3800	0.24	[−0.14; 0.61]	0.2%	0.4%
Wu, 2019	40	3.66	1.6100	40	3.07	1.1400	0.42	[−0.02; 0.86]	0.1%	0.4%
Kang, 2019	72	1.36	0.5400	100	1.15	0.4700	0.42	[0.11; 0.72]	0.3%	0.4%
Huang, 2019	33	2.07	0.7400	293	1.84	0.8000	0.29	[−0.07; 0.65]	0.2%	0.4%
Alhabri, 2019	200	1.80	0.8900	200	1.50	0.8100	0.35	[0.15; 0.55]	0.6%	0.4%
Anjum, 2019	25	1.38	0.5800	50	1.19	0.4700	0.37	[−0.11; 0.85]	0.1%	0.3%
Layton, 2018	67	1.97	0.7600	739	1.82	0.5600	0.26	[0.01; 0.51]	0.4%	0.4%
Wang, 2019	300	2.57	1.1300	1283	2.23	0.9300	0.35	[0.22; 0.48]	1.5%	0.4%
Abo−Elmatty, 2018	85	2.52	1.9100	80	1.96	0.3900	0.40	[0.09; 0.71]	0.3%	0.4%
Abo−Elmatty, 2018	85	2.47	0.5500	80	1.96	0.3900	1.06	[0.73; 1.39]	0.2%	0.4%
Alyas S, 2019	58	5.09	0.3800	100	4.79	0.4000	0.76	[0.43; 1.09]	0.2%	0.4%
Benhalima K, 2019	189	2.10	0.7400	1113	1.80	0.5900	0.49	[0.33; 0.64]	1.0%	0.4%
Wang F, 2019	100	2.48	0.4200	100	1.58	0.2800	2.51	[2.14; 2.88]	0.2%	0.4%
Abd KI, 2020	45	2.74	1.1400	45	1.38	0.8100	1.36	[0.90; 1.82]	0.1%	0.3%
Bulmus F, 2020	30	2.56	0.9200	30	2.05	0.7100	0.61	[0.09; 1.13]	0.1%	0.3%
Jack−Roberts C, 2020	33	2.60	1.3300	29	1.85	0.6800	0.69	[0.17; 1.20]	0.1%	0.3%
Khosrowbeygi A, 2020	49	2.81	0.1900	51	2.43	0.1500	2.21	[1.71; 2.71]	0.1%	0.3%
Liu Y, 2020	30	2.65	0.7600	30	2.46	0.9100	0.22	[−0.28; 0.73]	0.1%	0.3%
Mosavat M, 2020	53	2.68	2.6200	43	2.14	0.8500	0.26	[−0.14; 0.67]	0.2%	0.4%
Mohammed Ali D, 2020	60	2.07	1.7800	30	1.44	1.2600	0.38	[−0.06; 0.83]	0.1%	0.4%
Wei L, 2020	241	3.86	2.8700	391	3.01	1.1700	0.43	[0.26; 0.59]	0.9%	0.4%
Sun M, 2020	64	3.12	0.3200	128	3.02	0.2700	0.35	[0.04; 0.65]	0.3%	0.4%
Yavuzkir S, 2020	30	1.28	0.4400	30	0.87	0.2800	1.10	[0.55; 1.64]	0.1%	0.3%
Zhong L, 2020	40	2.37	1.0100	40	1.83	1.1300	0.50	[0.05; 0.94]	0.1%	0.4%
Liu L, 2020	61	2.24	1.4100	122	2.01	0.9700	0.20	[−0.11; 0.51]	0.3%	0.4%
Contreras−Duarte S, 2020	69	2.32	1.0700	41	1.90	0.6800	0.44	[0.05; 0.83]	0.2%	0.4%
Contreras−Duarte S, 2020	48	2.14	0.6800	41	1.90	0.6800	0.35	[−0.07; 0.77]	0.1%	0.4%
Gülcü Bulmus F, 2020	30	2.56	0.9200	30	2.05	0.7100	0.61	[0.09; 1.13]	0.1%	0.3%
O’Malley E, 2020	108	2.20	0.8000	94	1.90	0.7000	0.40	[0.12; 0.67]	0.3%	0.4%
Liu Y, 2020	110	3.26	1.1700	100	1.66	0.5000	1.74	[1.42; 2.06]	0.2%	0.4%
Liu T, 2020	300	1.10	0.2000	261	1.35	0.3200	−0.95	[−1.13; −0.78]	0.8%	0.4%
Zhu H, 2020	581	2.00	0.7000	2368	1.70	0.6000	0.48	[0.39; 0.57]	2.9%	0.4%
Lu W, 2021	199	2.33	0.8600	199	1.93	0.6500	0.52	[0.32; 0.72]	0.6%	0.4%
Mei J, 2021	104	2.11	0.9200	104	1.88	0.6100	0.29	[0.02; 0.57]	0.3%	0.4%
Meng X, 2021	34	2.86	1.4400	34	1.95	0.5700	0.82	[0.33; 1.32]	0.1%	0.3%
Sudana N, 2021	25	3.25	0.3400	50	2.69	0.6800	0.94	[0.43; 1.44]	0.1%	0.3%
Raczkowska B, 2021	26	1.96	0.6900	29	2.07	0.7700	−0.15	[−0.68; 0.38]	0.1%	0.3%
Ramasammy R, 2021	210	1.91	0.3300	209	1.86	0.3100	0.16	[−0.04; 0.35]	0.7%	0.4%
Ramanjaneya M, 2021a	34	1.50	0.9000	16	1.20	0.7000	0.35	[−0.25; 0.95]	0.1%	0.3%
Ramanjaneya M, 2021b	46	1.40	0.8000	26	1.20	0.6000	0.27	[−0.21; 0.75]	0.1%	0.3%
Kapustin V, 2021	88	2.90	0.3900	38	2.78	0.3600	0.31	[−0.07; 0.70]	0.2%	0.4%
Kapustin V, 2021	42	3.41	0.8100	38	2.78	0.3600	0.98	[0.51; 1.44]	0.1%	0.3%
Zhang L, 2021	36	1.42	1.1000	38	2.31	0.6700	−0.97	[−1.46; −0.49]	0.1%	0.3%
Rakchna M, 2021	73	1.97	0.7300	72	1.66	0.4700	0.50	[0.17; 0.83]	0.2%	0.4%
Kang M, 2021	116	1.83	0.7600	297	1.52	0.6000	0.48	[0.26; 0.69]	0.5%	0.4%
Baryla I, 2021	98	2.36	0.6800	37	2.36	0.6800	0.00	[−0.38; 0.38]	0.2%	0.4%
Abd−Eltawab A, 2020	109	2.56	0.5500	103	1.76	0.3700	1.69	[1.38; 2.01]	0.2%	0.4%
Ren M, 2021	56	6.69	0.7600	56	5.31	0.6700	1.91	[1.46; 2.36]	0.1%	0.4%
Xu D, 2021	80	2.83	0.9400	80	2.58	1.0900	0.24	[−0.07; 0.56]	0.3%	0.4%
Benhalima K, 2021	97	2.00	0.5900	327	1.70	0.3000	0.78	[0.54; 1.01]	0.5%	0.4%
Tabacu C, 2021	16	3.67	1.5100	83	2.12	0.7400	1.70	[1.12; 2.29]	0.1%	0.3%
Eppel D, 2021	27	2.47	0.5500	40	1.33	0.3700	2.50	[1.85; 3.15]	0.1%	0.3%
Onat T, 2021	60	1.81	0.5600	52	1.54	0.6300	0.45	[0.08; 0.83]	0.2%	0.4%
Wang Y, 2021	855	2.50	0.7000	785	1.80	0.6000	1.07	[0.97; 1.17]	2.3%	0.4%
Bulut A, 2021	22	2.29	0.6500	29	1.95	0.5800	0.55	[−0.02; 1.11]	0.1%	0.3%
Yu X, 2021	573	2.67	1.2000	740	2.37	1.0300	0.27	[0.16; 0.38]	2.0%	0.4%
Shalabi T, 2021	114	1.39	0.2000	114	1.28	0.1000	0.69	[0.43; 0.96]	0.3%	0.4%
Adiga U, 2021	100	2.68	0.4300	100	2.79	0.8200	−0.17	[−0.45; 0.11]	0.3%	0.4%
Cheng E, 2022	163	2.09	0.4300	145	1.33	0.2900	2.04	[1.77; 2.32]	0.3%	0.4%
Huang G, 2022	566	2.67	1.2100	710	2.39	1.0100	0.25	[0.14; 0.36]	2.0%	0.4%
Juchnicka I, 2022	24	1.80	0.5800	24	1.56	0.4200	0.47	[−0.11; 1.04]	0.1%	0.3%
Liu L, 2021	69	1.85	0.5600	71	1.98	0.7100	−0.20	[−0.53; 0.13]	0.2%	0.4%
Obaidullah M, 2021	132	2.50	0.7600	100	1.85	0.6700	0.90	[0.62; 1.17]	0.3%	0.4%
Adiga U, 2022	74	2.97	1.1900	132	2.62	0.8000	0.36	[0.08; 0.65]	0.3%	0.4%
Shen L, 2023	233	2.22	0.7900	1001	2.12	0.8500	0.12	[−0.02; 0.26]	1.2%	0.4%
Sahoo D, 2022	20	2.35	0.1300	45	2.12	0.0800	2.32	[1.65; 2.99]	0.1%	0.3%
Khan S, 2022	50	2.18	1.5600	226	1.72	0.7800	0.47	[0.17; 0.78]	0.3%	0.4%
Arslan A, 2022	40	2.02	0.5900	37	2.04	0.7300	−0.03	[−0.48; 0.42]	0.1%	0.4%
Hu W, 2022	315	1.58	0.7200	1876	1.35	0.6000	0.37	[0.25; 0.49]	1.7%	0.4%
Wu Y, 2022	130	1.97	1.0300	170	1.68	0.8100	0.32	[0.09; 0.55]	0.5%	0.4%
Cibickova L, 2022	43	2.20	0.8300	19	1.60	0.4500	0.80	[0.24; 1.36]	0.1%	0.3%
Karagoz ZK, 2022	30	2.37	0.8500	30	2.01	0.3300	0.55	[0.03; 1.07]	0.1%	0.3%
Wang, Y 2022	232	2.56	0.9600	696	2.21	0.8300	0.40	[0.25; 0.55]	1.1%	0.4%
Jia X, 2022	62	1.75	0.5900	58	1.23	0.3400	1.06	[0.68; 1.45]	0.2%	0.4%
Zeljkovic A, 2022	15	2.35	0.8800	48	1.91	0.5300	0.69	[0.10; 1.29]	0.1%	0.3%
Wang J, 2023	47	1.26	0.5400	47	1.02	0.3100	0.54	[0.13; 0.95]	0.1%	0.4%
Ma, 2023	110	3.15	1.1500	110	2.62	0.9600	0.50	[0.23; 0.77]	0.3%	0.4%
Akash, 2023	400	1.96	0.6200	100	1.75	0.1900	0.37	[0.15; 0.59]	0.5%	0.4%
Hou, 2021	189	3.02	0.4900	100	2.28	0.3000	1.70	[1.42; 1.98]	0.3%	0.4%
Kagitci, 2023	30	2.47	1.1900	30	2.18	0.7500	0.29	[−0.22; 0.80]	0.1%	0.3%
Gawlik, 2023	74	2.03	0.7300	32	2.32	0.5600	−0.42	[−0.84; 0.00]	0.1%	0.4%
Burzynska−Pedziwiatr, 2023	32	2.33	1.0000	11	1.78	0.9900	0.54	[−0.15; 1.24]	0.1%	0.3%
Štambuk, 2023	41	2.12	0.7500	48	1.66	0.6200	0.67	[0.24; 1.10]	0.1%	0.4%
Shi, 2023	1632	2.52	0.8400	9067	2.02	0.8100	0.61	[0.56; 0.67]	8.6%	0.4%
Li, 2023	148	1.78	0.8100	296	1.63	0.7000	0.20	[0.01; 0.40]	0.6%	0.4%
Ali Khan, 2023	90	1.87	1.0700	90	1.54	2.1100	0.20	[−0.10; 0.49]	0.3%	0.4%
Linares−Pineda, 2023	16	2.07	0.4300	16	2.19	0.7700	−0.19	[−0.88; 0.51]	0.1%	0.3%
Shen, 2024	12	2.50	0.1900	10	2.37	0.3700	0.44	[−0.41; 1.29]	0.0%	0.2%
Thilak, 2024	60	2.19	0.6700	60	2.11	0.4400	0.14	[−0.22; 0.50]	0.2%	0.4%
Ortega−Montiel, 2024	294	2.40	0.9000	1762	2.00	0.8000	0.49	[0.37; 0.61]	1.6%	0.4%
Ortega−Montiel, 2024	158	2.50	1.0000	1762	2.00	0.8000	0.61	[0.45; 0.77]	0.9%	0.4%
Ortega−Montiel, 2024	101	2.20	0.7000	1762	2.00	0.8000	0.25	[0.05; 0.45]	0.6%	0.4%
Ortega−Montiel, 2024	35	2.20	0.6000	1762	2.00	0.8000	0.25	[−0.08; 0.59]	0.2%	0.4%
Özer, 2024	44	3.20	0.9900	44	2.87	0.8700	0.35	[−0.07; 0.77]	0.1%	0.4%
Ghaneialvar, 2024	40	2.30	0.2500	40	1.95	0.3200	1.21	[0.73; 1.69]	0.1%	0.3%
Aziz, 2024	50	0.83	0.2800	50	0.66	0.2100	0.68	[0.28; 1.09]	0.2%	0.4%
Uysal, 2024	52	2.61	0.8600	31	2.40	0.7200	0.26	[−0.19; 0.70]	0.1%	0.4%
Wu, 2024	35	2.22	1.1600	70	1.89	0.5400	0.41	[0.00; 0.82]	0.1%	0.4%
Li, 2024	1323	1.30	0.5300	1421	1.30	0.5300	0.00	[−0.07; 0.07]	4.4%	0.4%
Ademoglu EN, 2015	47	2.60	1.0000	32	2.10	0.7000	0.56	[0.10; 1.01]	0.1%	0.3%
Anghebem−Oliveira MI, 2017	127	2.50	0.8700	125	1.41	0.5900	1.46	[1.18; 1.74]	0.3%	0.4%
Boghossian NS, 2017	93	2.70	1.3000	136	1.99	0.6900	0.72	[0.45; 0.99]	0.3%	0.4%
Dudzik D, 2014	20	2.19	0.6400	20	1.58	0.6000	0.96	[0.31; 1.62]	0.1%	0.3%
Dudzik D, 2017	24	2.08	0.6500	24	1.68	0.5100	0.67	[0.09; 1.26]	0.1%	0.3%
Yorulmaz Ertug E, 2016	29	2.49	0.8900	20	1.80	0.7000	0.83	[0.23; 1.42]	0.1%	0.3%
Guimarães LO,2014	150	2.54	0.8300	295	1.39	0.6500	1.60	[1.38; 1.83]	0.5%	0.4%
Inegol Gumus I, 2013	34	1.25	0.6400	50	1.03	0.3600	0.44	[0.00; 0.88]	0.1%	0.4%
Hollingsworth DR, 1982	5	2.26	0.4500	21	1.76	0.3800	1.23	[0.19; 2.27]	0.0%	0.2%
Huang Y, 2018	88	1.33	0.5500	386	1.24	0.3900	0.21	[−0.02; 0.44]	0.5%	0.4%
Knopp RH, 1980	22	2.34	1.2400	38	2.14	0.6600	0.22	[−0.31; 0.74]	0.1%	0.3%
Knopp RH, 1992	95	2.29	0.6800	521	1.86	0.6800	0.63	[0.41; 0.85]	0.5%	0.4%
Liang Z, 2015	38	3.20	1.3000	35	2.40	0.9000	0.70	[0.23; 1.18]	0.1%	0.3%
Liu B, 2016	276	2.31	0.8400	1270	2.09	0.7600	0.28	[0.15; 0.41]	1.4%	0.4%
Liu B, 2016	28	3.06	1.2500	25	2.73	1.0000	0.29	[−0.26; 0.83]	0.1%	0.3%
Rojas I, 2002	38	2.17	0.8000	38	1.95	0.6100	0.31	[−0.15; 0.76]	0.1%	0.3%
Whyte K, 2013	22	2.33	0.7800	167	1.84	0.8600	0.57	[0.12; 1.02]	0.1%	0.4%
Anderlová K, 2022	36	1.85	0.6600	22	2.49	0.8400	−0.86	[−1.42; −0.31]	0.1%	0.3%
Attique H, 2022	37	2.14	0.6100	47	1.92	0.5900	0.36	[−0.07; 0.80]	0.1%	0.4%
Beunen K, 2014	231	2.00	0.4000	1612	1.70	0.3000	0.95	[0.81; 1.10]	1.2%	0.4%
Botta RM, 1997	75	2.62	1.2500	75	1.67	0.7900	0.90	[0.57; 1.24]	0.2%	0.4%
Chen Y, 2017	28	2.48	0.9300	56	2.09	0.7300	0.48	[0.02; 0.94]	0.1%	0.3%
Diboun I, 2020	32	1.40	0.9000	21	1.20	0.6000	0.25	[−0.31; 0.80]	0.1%	0.3%
Franzago M, 2020	50	2.80	0.8000	50	1.90	0.5000	1.34	[0.90; 1.77]	0.1%	0.4%
Grieger J, 2021	103	2.10	1.0000	2812	1.50	0.6000	0.97	[0.77; 1.17]	0.6%	0.4%
Guan L, 2021	461	3.88	1.6700	626	3.62	1.4600	0.17	[0.05; 0.29]	1.7%	0.4%
Guo Y, 2020	87	1.40	0.5900	88	1.20	0.3700	0.40	[0.11; 0.70]	0.3%	0.4%
Huo Y, 2019	30	2.79	0.7700	28	2.52	0.7100	0.36	[−0.16; 0.88]	0.1%	0.3%
Iimura Y, 2015	53	2.21	0.3600	1108	1.94	0.2600	1.02	[0.74; 1.30]	0.3%	0.4%
Kralisch S, 2009	40	2.20	1.3000	80	2.10	1.4000	0.07	[−0.31; 0.45]	0.2%	0.4%
Li J, 2015	35	3.89	1.0200	43	2.57	1.0200	1.28	[0.79; 1.77]	0.1%	0.3%
Li J, 2017	100	2.92	1.3600	100	2.51	0.7900	0.37	[0.09; 0.65]	0.3%	0.4%
Li F, 2020	48	2.08	1.3300	202	1.95	0.7500	0.15	[−0.17; 0.46]	0.2%	0.4%
Li Y, 2021	359	2.38	1.3700	273	2.05	0.9700	0.27	[0.11; 0.43]	1.0%	0.4%
Lu L, 2023	83	3.79	1.4100	649	3.03	1.1400	0.65	[0.42; 0.88]	0.5%	0.4%
Ma J, 2022	31	2.24	0.3800	69	2.17	0.4000	0.18	[−0.25; 0.60]	0.1%	0.4%
Meyer B, 2023	43	3.27	1.1500	26	2.41	0.8100	0.82	[0.31; 1.33]	0.1%	0.3%
Onat T, 2021	40	1.61	0.7900	40	1.62	0.5800	−0.01	[−0.45; 0.42]	0.1%	0.4%
Qin L, 2023	82	2.25	0.8000	89	2.10	0.6400	0.21	[−0.09; 0.51]	0.3%	0.4%
Sun J, 2022	80	2.51	0.9800	79	2.03	0.7100	0.56	[0.24; 0.87]	0.2%	0.4%
Takhshid M, 2015	75	3.02	1.3700	70	3.02	1.1500	0.00	[−0.33; 0.33]	0.2%	0.4%
Theodorakopoulou M, 2021	21	2.20	0.9600	15	2.20	0.6700	0.00	[−0.66; 0.66]	0.1%	0.3%
Tönjes A, 2019	74	2.47	1.0700	74	2.36	0.9400	0.11	[−0.21; 0.43]	0.2%	0.4%
Torun G, 2023	50	2.80	0.9800	50	2.18	0.8400	0.67	[0.27; 1.08]	0.2%	0.4%
Tutar D, 2022	40	2.27	0.7800	40	2.03	0.7500	0.31	[−0.13; 0.75]	0.1%	0.4%
Wang D, 2015	110	2.18	0.7300	526	1.95	0.6100	0.36	[0.16; 0.57]	0.6%	0.4%
Wang X, 2015	839	2.54	0.8700	900	2.22	0.6800	0.41	[0.32; 0.51]	2.7%	0.4%
Wang H, 2019	35	3.69	0.7000	35	3.23	0.6000	0.70	[0.21; 1.18]	0.1%	0.3%
Wang H, 2019	35	3.89	0.9000	35	3.55	0.8000	0.39	[−0.08; 0.87]	0.1%	0.3%
Wójcik M, 2015	77	2.97	1.0000	34	2.53	0.8000	0.46	[0.05; 0.87]	0.1%	0.4%
Xu L, 2013	120	2.51	0.8500	60	2.24	0.4400	0.36	[0.05; 0.68]	0.3%	0.4%
Xu M, 2015	55	3.03	1.0100	66	2.60	0.8300	0.47	[0.10; 0.83]	0.2%	0.4%
Yakout S, 2023	46	1.47	0.6700	77	1.29	0.5900	0.29	[−0.08; 0.66]	0.2%	0.4%
Yi J, 2018	141	2.39	0.7900	230	2.27	0.8300	0.15	[−0.06; 0.36]	0.6%	0.4%
Zhang Y, 2015	186	2.37	1.7200	330	2.33	1.3100	0.03	[−0.15; 0.21]	0.8%	0.4%
Zhang H, 2022	110	2.02	1.0600	416	1.62	0.6500	0.53	[0.32; 0.74]	0.5%	0.4%
**Fixed effect model**	**28292**			**64613**			**0.49**	**[0.47; 0.50]**	**100.0%**	**−−**
**Random effects model**							**0.57**	**[0.51; 0.63]**	**−−**	**100.0%**

Heterogeneity: $I^2 = 92\%$, $\tau^2 = 0.2014$, $p = 0$

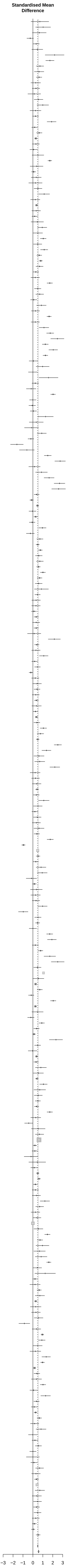

Supplement: Supplementary file 1 [file medicina-61-01290-s001.zip › Figure S20 TG 2nd trimester.pdf]
