# Supplementary material for: Trimester-Specific Serum Lipid Profiles in Gestational Diabetes Mellitus: A Systematic Review, Meta-Analysis, and Meta-Regression
Source: Medicina (Kaunas). 2025 Jul 17;61(7):1290. doi: 10.3390/medicina61071290 (PMC12300116; doi:10.3390/medicina61071290)

Study	Experimental Total	Experimental Mean	Experimental SD	Control Total	Control Mean	Control SD	SMD	95%−CI	Weight (fixed)	Weight (random)
Hornnes P, 1984	9	2.84	0.7500	8.000	2.28	0.9900	0.61	[−0.37; 1.59]	0.1%	0.5%
Montelongo, 1992	9	2.06	0.6900	12.000	2.03	0.9000	0.04	[−0.83; 0.90]	0.1%	0.5%
Nolan C, 1995	38	2.57	1.3900	350.000	2.15	0.9600	0.42	[0.08; 0.75]	0.6%	1.0%
Meyer B, 1996	44	2.61	1.2400	36.000	2.26	1.2400	0.28	[−0.16; 0.72]	0.3%	0.9%
Koukkou E, 1997	20	2.92	1.9400	22.000	2.10	0.5700	0.57	[−0.04; 1.19]	0.2%	0.7%
Couch S, 1998	25	2.04	0.5100	25.000	1.53	0.4600	1.03	[0.44; 1.63]	0.2%	0.7%
Bartha J, 2000	34	2.79	0.9400	32.000	2.63	0.8900	0.17	[−0.31; 0.66]	0.3%	0.8%
Paradisi G, 2002	13	2.25	0.1100	15.000	2.47	0.5400	−0.53	[−1.29; 0.23]	0.1%	0.6%
Vitoratos G, 2002	15	3.05	1.0100	21.000	3.50	0.9500	−0.45	[−1.12; 0.22]	0.1%	0.7%
Toescu V, 2004	12	2.80	0.6000	17.000	2.95	0.9000	−0.18	[−0.92; 0.56]	0.1%	0.6%
Ranheim T, 2004	22	3.40	0.3000	29.000	2.90	1.1000	0.58	[0.01; 1.14]	0.2%	0.8%
Tsai P, 2005	34	2.90	1.0000	219.000	2.70	1.0000	0.20	[−0.16; 0.56]	0.5%	0.9%
Grissa O, 2007	59	3.07	0.2500	60.000	3.07	0.2700	0.00	[−0.36; 0.36]	0.5%	0.9%
Sánchez−Vera I, 2007	62	2.10	0.9400	45.000	1.70	0.6500	0.48	[0.09; 0.87]	0.4%	0.9%
Bartha J, 2008	30	2.70	0.8500	20.000	2.80	0.9900	−0.11	[−0.67; 0.46]	0.2%	0.8%
Szymanska M, 2008	81	2.80	0.8100	41.000	2.31	0.7000	0.63	[0.24; 1.01]	0.5%	0.9%
Akturk M, 2008	47	3.11	1.0300	31.000	2.64	0.8400	0.48	[0.02; 0.95]	0.3%	0.8%
Pfau D, 2010	40	2.20	1.3000	80.000	2.10	1.4000	0.07	[−0.31; 0.45]	0.5%	0.9%
Paradisi G, 2010	12	2.70	0.8700	38.000	2.39	1.4800	0.22	[−0.43; 0.87]	0.2%	0.7%
Akturk M, 2010	54	3.08	1.5000	69.000	2.60	1.1600	0.36	[0.00; 0.72]	0.5%	0.9%
Retnakaran R, 2010	136	2.49	0.7000	87.000	2.59	0.8400	−0.13	[−0.40; 0.14]	0.9%	1.0%
Culha C, 2011	24	2.80	0.4400	20.000	2.56	0.4600	0.52	[−0.08; 1.13]	0.2%	0.7%
Saucedo R, 2011	60	3.25	1.1500	60.000	2.65	1.1100	0.53	[0.16; 0.89]	0.5%	0.9%
Giannubilo S, 2011	40	1.86	0.3100	40.000	1.82	0.2500	0.14	[−0.30; 0.58]	0.4%	0.9%
Giannubilo S, 2011	40	2.55	0.7800	40.000	2.15	0.5600	0.58	[0.14; 1.03]	0.3%	0.9%
Giannubilo S, 2011	40	2.89	0.6300	40.000	2.58	0.4800	0.55	[0.10; 1.00]	0.3%	0.9%
Ghafoor S, 2012	46	2.43	1.1500	50.000	2.10	0.9200	0.32	[−0.09; 0.72]	0.4%	0.9%
Farhan S, 2012	10	2.33	0.2800	10.000	2.22	0.7000	0.20	[−0.68; 1.08]	0.1%	0.5%
Cocelli V, 2012	62	3.07	0.9800	61.000	2.35	0.8800	0.77	[0.40; 1.13]	0.5%	0.9%
Gkiomisi A, 2013	44	3.55	2.2600	44.000	2.84	1.7900	0.35	[−0.08; 0.77]	0.4%	0.9%
Park S, 2013	117	3.17	1.2300	136.000	2.37	0.8100	0.78	[0.52; 1.03]	1.0%	1.0%
Khan R, 2013	103	2.13	0.2200	97.000	1.90	0.2400	1.00	[0.70; 1.29]	0.8%	1.0%
dos Santos−Weiss I, 2012	288	2.50	0.8100	288.000	1.90	0.7400	0.77	[0.60; 0.94]	2.4%	1.1%
Soydinc S, 2013	42	2.52	0.8800	33.000	2.14	1.0800	0.39	[−0.07; 0.85]	0.3%	0.8%
Kärkkäinen H, 2013	42	2.66	0.1200	32.000	2.12	0.5100	1.54	[1.02; 2.07]	0.2%	0.8%
Agakidou E, 2013	27	3.29	0.9000	27.000	3.00	1.1000	0.28	[−0.25; 0.82]	0.2%	0.8%
Eslamian R, 2013	112	1.97	0.2700	159.000	1.91	0.3000	0.21	[−0.03; 0.45]	1.2%	1.0%
Eslamian R, 2013	112	2.70	0.3600	159.000	2.28	0.3500	1.18	[0.92; 1.44]	1.0%	1.0%
Ortega−Senovilla S, 2013	40	2.80	0.1300	45.000	2.89	0.1300	−0.69	[−1.12; −0.25]	0.4%	0.9%
Yousefzadeh G, 2013	60	2.91	1.0000	30.000	2.90	1.3800	0.01	[−0.43; 0.45]	0.4%	0.9%
Al−Hakeem M, 2014	200	2.30	1.8000	300.000	1.70	0.9800	0.44	[0.26; 0.62]	2.1%	1.1%
Wójcik M, 2014	132	2.82	0.5300	43.000	2.60	0.8600	0.35	[0.00; 0.70]	0.6%	0.9%
Al Rubeaan, 2014	201	1.84	0.9500	328.000	1.81	0.9400	0.03	[−0.14; 0.21]	2.2%	1.1%
Megia, 2014	79	2.17	0.7000	78.000	1.99	0.6600	0.26	[−0.05; 0.58]	0.7%	1.0%
Du M, 2015	38	2.05	0.4800	38.000	1.59	0.4700	0.96	[0.48; 1.43]	0.3%	0.8%
Zhang Y, 2016	40	2.85	1.2100	240.000	1.74	0.5300	1.66	[1.29; 2.02]	0.5%	0.9%
Savona−Ventura C, 2016	459	3.40	2.3000	603.000	2.10	0.8000	0.80	[0.67; 0.92]	4.3%	1.1%
Yang X, 2017	19	4.96	0.5900	20.000	4.62	0.4300	0.65	[0.00; 1.29]	0.2%	0.7%
Zhang Y, 2017	50	2.94	1.5300	50.000	2.12	0.9000	0.65	[0.25; 1.05]	0.4%	0.9%
Burlina S, 2016	21	1.92	0.5300	21.000	1.78	0.6000	0.24	[−0.36; 0.85]	0.2%	0.7%
Hussain Z, 2018	60	2.83	0.2800	60.000	1.78	0.1900	4.36	[3.70; 5.03]	0.2%	0.7%
Yuan X, 2018	86	4.09	1.0400	273.000	3.48	1.2500	0.51	[0.26; 0.75]	1.1%	1.0%
Zhang Y, 2018	50	3.80	1.5000	47.000	3.60	2.6000	0.09	[−0.30; 0.49]	0.4%	0.9%
Bao W, 2018	107	2.92	1.5500	214.000	2.70	1.7600	0.13	[−0.10; 0.36]	1.3%	1.0%
Bugatto F, 2018	22	2.85	0.9300	23.000	2.16	0.7800	0.79	[0.18; 1.40]	0.2%	0.7%
Al−Daghri NM, 2019	39	2.30	1.0000	63.000	2.10	0.8000	0.23	[−0.18; 0.63]	0.4%	0.9%
Ma Y, 2019	37	4.24	2.0300	97.000	3.77	1.6000	0.27	[−0.11; 0.65]	0.5%	0.9%
Wu, 2019	65	3.61	1.5900	65.000	2.76	0.9800	0.64	[0.29; 0.99]	0.5%	0.9%
Kang, 2019	72	3.78	1.1900	100.000	3.30	1.3000	0.38	[0.08; 0.69]	0.7%	1.0%
Wang, 2019	300	3.36	1.5100	1283.000	3.08	1.2300	0.22	[0.09; 0.34]	4.3%	1.1%
Aydemir B, 2019	99	2.30	0.8500	98.000	2.23	0.8400	0.08	[−0.20; 0.36]	0.9%	1.0%
Fan Y, 2020	65	4.66	0.2500	55.000	3.01	0.1300	8.04	[6.94; 9.13]	0.1%	0.4%
Mohammed Ali D, 2020	60	2.49	1.3200	30.000	1.60	0.9300	0.73	[0.28; 1.18]	0.3%	0.9%
Contreras−Duarte S, 2020	69	2.59	0.9800	41.000	2.73	0.9500	−0.14	[−0.53; 0.24]	0.5%	0.9%
Contreras−Duarte S, 2020	48	2.54	0.8000	41.000	2.73	0.9500	−0.22	[−0.63; 0.20]	0.4%	0.9%
Liu M, 2020	50	3.71	1.2900	47.000	3.41	1.0700	0.25	[−0.15; 0.65]	0.4%	0.9%
Li G, 2021	23	3.09	1.2000	29.000	2.11	0.6900	1.02	[0.43; 1.60]	0.2%	0.7%
Hussain Z, 2021	60	2.77	2.0900	60.000	1.65	1.0100	0.68	[0.31; 1.05]	0.5%	0.9%
Wu L, 2021	213	2.36	0.6800	191.000	2.08	0.8900	0.36	[0.16; 0.55]	1.7%	1.0%
Zhou J, 2021	50	3.30	1.0400	50.000	2.79	1.0700	0.48	[0.08; 0.88]	0.4%	0.9%
Wang F, 2021	53	3.65	1.4600	46.000	2.92	1.3600	0.51	[0.11; 0.91]	0.4%	0.9%
Balachandiran M, 2021	40	2.29	0.9100	40.000	1.60	0.4100	0.97	[0.50; 1.43]	0.3%	0.8%
Abdualhay R, 2022	44	2.05	0.3300	45.000	1.59	0.2700	1.51	[1.04; 1.99]	0.3%	0.8%
Franzago M, 2022	33	2.29	0.4100	27.000	2.45	0.7200	−0.28	[−0.79; 0.23]	0.3%	0.8%
Dualib P, 2022	56	2.24	0.7700	59.000	1.50	0.6000	1.07	[0.68; 1.46]	0.4%	0.9%
Yang J, 2022	21	3.94	1.6400	60.000	3.37	0.8500	0.51	[0.01; 1.01]	0.3%	0.8%
Parveen S, 2022	37	2.77	0.2200	163.000	2.32	0.4800	1.01	[0.64; 1.38]	0.5%	0.9%
Mahmood K, 2022	50	1.10	0.0700	50.000	1.03	0.1600	0.56	[0.16; 0.96]	0.4%	0.9%
Sobczynska−Malefora A, 2021	24	2.10	0.5900	35.000	2.40	1.1000	−0.32	[−0.84; 0.20]	0.2%	0.8%
Han L, 2022	100	1.88	0.2100	100.000	1.36	0.1700	2.71	[2.33; 3.10]	0.5%	0.9%
Yuan J, 2022	23	2.26	0.8200	19.000	1.76	0.5900	0.68	[0.05; 1.30]	0.2%	0.7%
Algaba−Chueca F, 2022	62	2.30	0.9200	74.000	2.32	0.8900	−0.02	[−0.36; 0.32]	0.6%	1.0%
Bernea E, 2022	10	2.59	0.8500	8.000	1.65	0.3300	1.33	[0.28; 2.38]	0.1%	0.4%
Zeljkovic A, 2022	15	3.27	0.9300	48.000	2.43	0.7500	1.04	[0.43; 1.65]	0.2%	0.7%
Zheng W, 2022	396	3.39	1.7100	2789.000	2.98	1.1100	0.34	[0.24; 0.45]	6.1%	1.1%
Wang J, 2023	47	3.00	1.1000	47.000	3.01	1.0400	−0.01	[−0.41; 0.40]	0.4%	0.9%
Akhtar, 2023	100	2.54	1.1000	100.000	2.44	1.0600	0.09	[−0.19; 0.37]	0.9%	1.0%
Visiedo, 2023	20	3.10	0.9500	17.000	2.28	0.7600	0.92	[0.24; 1.61]	0.1%	0.7%
Jiang, 2023	719	3.92	1.7100	1205.000	3.64	1.4200	0.18	[0.09; 0.27]	7.9%	1.1%
Shi, 2023	1632	3.83	1.6700	9067.000	3.34	1.3400	0.35	[0.30; 0.40]	24.2%	1.1%
Linares−Pineda, 2023	16	2.73	0.8900	16.000	2.81	1.2700	−0.07	[−0.76; 0.62]	0.1%	0.7%
Nahdh Saleem, 2024	30	1.64	0.6100	22.000	2.03	0.6200	−0.63	[−1.19; −0.06]	0.2%	0.8%
Nahdh Saleem, 2024	24	2.07	0.6400	22.000	2.03	0.6200	0.06	[−0.52; 0.64]	0.2%	0.7%
Heiskanen N, 2010	51	2.70	1.0000	28.000	2.30	0.8000	0.42	[−0.04; 0.89]	0.3%	0.8%
Hsu HW, 1997	8	2.72	1.3200	8.000	2.33	1.0200	0.31	[−0.68; 1.30]	0.1%	0.5%
Huo Y, 2015	30	3.26	1.0300	27.000	2.80	0.9300	0.46	[−0.07; 0.99]	0.2%	0.8%
Keskin FE, 2015	44	2.35	0.9400	45.000	2.53	0.9700	−0.19	[−0.60; 0.23]	0.4%	0.9%
Liao Y, 2018	16	3.72	1.8800	16.000	3.57	2.4300	0.07	[−0.63; 0.76]	0.1%	0.7%
Maitland RA, 2014	29	1.67	1.4200	77.000	1.53	1.3800	0.10	[−0.33; 0.53]	0.4%	0.9%
Maple−Brown, 2012	198	2.70	0.8000	477.000	2.40	0.8000	0.37	[0.21; 0.54]	2.4%	1.1%
Marseille−Tremblay C, 2018	7	3.01	0.4900	7.000	2.95	0.4700	0.12	[−0.93; 1.17]	0.1%	0.4%
Metzger BE, 1980	6	5.00	0.6000	8.000	2.10	0.3000	6.03	[3.19; 8.87]	0.0%	0.1%
Miettinen HE, 2014	22	2.76	0.2400	30.000	2.44	0.1700	1.56	[0.93; 2.19]	0.2%	0.7%
Miettine HE, 2018	15	2.53	0.1800	13.000	2.35	0.2100	0.90	[0.11; 1.68]	0.1%	0.6%
Morimitsu LK, 2007	23	2.26	0.6900	11.000	1.66	0.6400	0.87	[0.12; 1.62]	0.1%	0.6%
Mrizak I, 2013	28	2.40	0.9300	24.000	1.99	0.2300	0.58	[0.02; 1.13]	0.2%	0.8%
Mrizak I, 2014	30	2.48	0.1500	30.000	1.92	1.5000	0.52	[0.00; 1.03]	0.3%	0.8%
Sreckovic I, 2014	9	2.50	0.4000	11.000	2.21	0.3000	0.80	[−0.13; 1.72]	0.1%	0.5%
Visiedo F, 2013	8	2.21	0.1400	14.000	2.10	0.7500	0.17	[−0.70; 1.04]	0.1%	0.5%
Weng Q, 2019	113	2.75	1.0400	396.000	2.79	1.4200	−0.03	[−0.24; 0.18]	1.5%	1.0%
Zakovicova E, 2014	35	2.75	1.0000	31.000	2.67	0.7600	0.09	[−0.40; 0.57]	0.3%	0.8%
Anderlová K, 2022	36	2.75	1.0200	22.000	2.77	1.0300	−0.02	[−0.55; 0.51]	0.2%	0.8%
Barrett HL, 2021	22	2.70	0.3000	7.000	3.20	0.7500	−1.10	[−2.00; −0.20]	0.1%	0.5%
Bronisz A, 2007	121	2.94	1.1900	36.000	2.49	0.8700	0.40	[0.02; 0.77]	0.5%	0.9%
Franzago M, 2021	33	2.20	0.6700	27.000	2.50	0.8000	−0.40	[−0.92; 0.11]	0.3%	0.8%
Genova M, 2024	54	2.30	0.8100	54.000	1.70	0.9600	0.67	[0.28; 1.06]	0.4%	0.9%
Guo Y, 2020	87	1.50	0.4400	88.000	1.30	0.5200	0.41	[0.11; 0.71]	0.8%	1.0%
Ozyildirim S, 2023	87	1.48	0.5100	36.000	1.37	0.6400	0.20	[−0.19; 0.59]	0.4%	0.9%
Prakash G, 2017	139	2.24	0.5900	139.000	1.59	0.4700	1.22	[0.96; 1.47]	1.0%	1.0%
Radojicic O, 2024	65	2.47	0.8600	49.000	2.13	0.6800	0.43	[0.04; 0.83]	0.4%	0.9%
Tang F, 2023	660	3.89	1.6900	1.097	3.67	1.4700	0.13	[−1.74; 2.00]	0.0%	0.2%
**Fixed effect model**	10435			22987.097			0.40	[0.37; 0.43]	100.0%	−−
**Random effects model**							0.48	[0.39; 0.57]	−−	100.0%

Heterogeneity: $I^2 = 89\%$, $\tau^2 = 0.1793$, $p < 0.01$

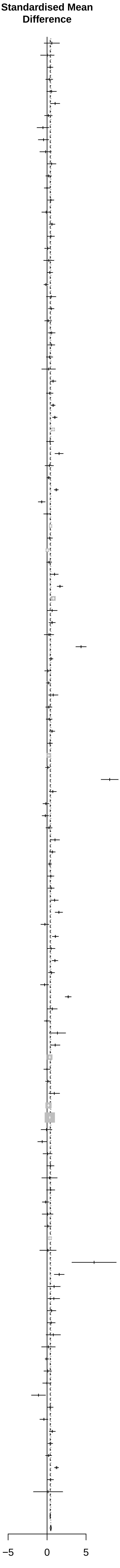

Supplement: Supplementary file 1 [file medicina-61-01290-s001.zip › Figure S21 TG 3rd trimester.pdf]
