# Supplementary material for: Trimester-Specific Serum Lipid Profiles in Gestational Diabetes Mellitus: A Systematic Review, Meta-Analysis, and Meta-Regression
Source: Medicina (Kaunas). 2025 Jul 17;61(7):1290. doi: 10.3390/medicina61071290 (PMC12300116; doi:10.3390/medicina61071290)
Supplement: Supplementary file 1 [file medicina-61-01290-s001.zip › Figure S22 Total choletserol 1st trimester.pdf]

Study	Experimental Total	Experimental Mean	Experimental SD	Control Total	Control Mean	Control SD	Standardised Mean Difference	SMD	95%−CI	Weight (fixed)	Weight (random)
Montelongo, 1992	9	4.67	1.1100	12	4.48	0.6200		0.21	[−0.66; 1.08]	0.0%	0.6%
Sánchez−Vera I, 2007	62	5.20	1.2700	45	4.30	1.3200		0.69	[0.30; 1.09]	0.2%	1.1%
Paradisi G, 2010	12	4.75	0.5500	38	4.81	1.0500		−0.06	[−0.71; 0.59]	0.1%	0.8%
Savvidou M, 2010	124	4.88	0.9000	248	4.59	0.8500		0.33	[0.12; 0.55]	0.7%	1.3%
Makgoba M, 2011	90	4.86	0.9000	158	4.62	0.7600		0.29	[0.03; 0.55]	0.5%	1.3%
dos Santos−Weiss I, 2012	288	5.00	1.0000	288	4.80	0.9000		0.21	[0.05; 0.37]	1.2%	1.4%
Li G, 2015	379	4.79	1.0800	2166	4.56	0.8200		0.27	[0.16; 0.38]	2.7%	1.4%
Wang C, 2016	1062	4.47	0.8300	4203	4.44	0.7800		0.04	[−0.03; 0.11]	7.2%	1.5%
Kumru P, 2016	38	5.02	1.0100	295	4.44	0.8500		0.67	[0.32; 1.01]	0.3%	1.2%
Yang X, 2017	19	4.75	0.2600	20	5.49	0.2600		−2.79	[−3.69; −1.88]	0.0%	0.5%
Yuan X, 2018	86	5.06	0.7800	273	4.98	0.7400		0.11	[−0.14; 0.35]	0.6%	1.3%
Hou W, 2018	131	4.80	0.9000	138	1.80	1.0000		3.14	[2.78; 3.50]	0.3%	1.2%
Yuan X, 2018	86	5.08	0.6700	273	4.92	0.6400		0.25	[0.00; 0.49]	0.6%	1.3%
Bao W, 2018	107	4.62	1.4500	214	4.52	1.3200		0.07	[−0.16; 0.31]	0.6%	1.3%
Madhu SV, 2019	45	5.87	2.9900	45	3.78	1.1700		0.91	[0.48; 1.35]	0.2%	1.1%
Bawah AT, 2019	21	7.24	0.1600	291	5.77	0.1700		8.66	[7.84; 9.47]	0.0%	0.6%
Pezeshki B, 2019	25	3.88	0.5200	301	3.79	0.4500		0.20	[−0.21; 0.61]	0.2%	1.1%
Correa, 2018	16	4.99	1.3600	80	4.28	0.7400		0.81	[0.26; 1.36]	0.1%	0.9%
Wang, 2019	300	4.07	0.6700	1283	3.84	0.7000		0.33	[0.20; 0.46]	2.0%	1.4%
Alyas S, 2019	58	8.00	0.2300	100	7.45	0.4000		1.57	[1.21; 1.94]	0.2%	1.1%
Zheng T, 2019	612	4.79	0.8200	4152	4.71	0.7900		0.10	[0.02; 0.19]	4.5%	1.5%
Jia H, 2019	136	5.66	1.2800	138	5.37	1.1500		0.24	[0.00; 0.48]	0.6%	1.3%
Benhalima K, 2019	189	4.90	0.8900	1113	4.60	0.8200		0.36	[0.21; 0.52]	1.4%	1.4%
Ma S, 2020	98	4.58	0.7300	98	4.49	0.7000		0.13	[−0.15; 0.41]	0.4%	1.3%
Mohammed Ali D, 2020	60	4.68	0.3300	30	4.35	0.3000		1.02	[0.56; 1.49]	0.2%	1.0%
Sun T, 2020	258	5.30	1.2700	1154	5.47	1.0900		−0.15	[−0.29; −0.02]	1.8%	1.4%
Ye Y, 2020	2181	4.71	0.7700	2719	4.60	0.7600		0.14	[0.09; 0.20]	10.2%	1.5%
Contreras−Duarte S, 2020	69	4.72	0.6700	41	4.72	0.8200		0.00	[−0.39; 0.39]	0.2%	1.1%
Jiang R, 2019	65	4.91	0.8300	366	4.73	0.8200		0.22	[−0.05; 0.48]	0.5%	1.3%
Deischinger C. 2020	45	5.89	1.2200	48	5.72	1.1100		0.14	[−0.26; 0.55]	0.2%	1.1%
Zhang X, 2020	274	4.14	0.7900	1111	3.91	0.6700		0.33	[0.20; 0.46]	1.8%	1.4%
McMichael L, 2021	34	5.27	1.2600	34	5.01	1.0600		0.22	[−0.26; 0.70]	0.1%	1.0%
Tian M, 2021	51	4.60	0.7000	51	4.40	0.7000		0.28	[−0.11; 0.67]	0.2%	1.1%
Wang X, 2021	607	4.65	0.7600	833	4.49	0.6900		0.22	[0.12; 0.33]	3.0%	1.4%
Wang Y, 2021	336	5.20	1.1000	672	5.10	1.1000		0.09	[−0.04; 0.22]	1.9%	1.4%
Catov J, 2021	1102	4.90	0.9000	3285	4.80	0.9000		0.11	[0.04; 0.18]	7.0%	1.5%
Coussa R, 2021	34	5.40	1.8000	124	5.10	1.5000		0.19	[−0.19; 0.57]	0.2%	1.1%
Kotzaeridi G, 2021	239	5.50	0.9000	893	5.10	0.8000		0.49	[0.34; 0.63]	1.6%	1.4%
Abdualhay R, 2022	44	5.56	0.1400	45	4.75	0.1100		6.39	[5.34; 7.43]	0.0%	0.4%
An R, 2022	94	4.63	0.7300	572	4.57	0.7900		0.08	[−0.14; 0.29]	0.7%	1.3%
Chen X, 2022	6	4.42	0.6000	27	4.90	0.8700		−0.56	[−1.46; 0.33]	0.0%	0.5%
Juchnicka I, 2022	24	4.39	0.5600	24	4.39	0.6200		0.00	[−0.57; 0.57]	0.1%	0.9%
Song S, 2022	249	4.09	0.8700	879	3.98	0.7600		0.14	[0.00; 0.28]	1.6%	1.4%
Shen L, 2023	233	5.11	0.9700	1001	4.94	0.7900		0.21	[0.06; 0.35]	1.6%	1.4%
Zheng Y, 2022	142	5.31	0.9400	442	4.85	0.8400		0.53	[0.34; 0.72]	0.9%	1.4%
Sahoo D, 2022	20	4.60	0.3000	45	4.54	0.1900		0.26	[−0.27; 0.79]	0.1%	0.9%
Song S, 2022	145	3.73	0.8400	555	3.70	0.7900		0.04	[−0.15; 0.22]	1.0%	1.4%
Aslan Çin N, 2022	46	5.53	3.3100	768	4.81	3.8200		0.19	[−0.11; 0.49]	0.4%	1.2%
Zeljkovic A, 2022	15	5.61	1.1300	48	5.24	1.0100		0.35	[−0.23; 0.94]	0.1%	0.9%
Zheng W, 2022	396	4.40	0.7100	2789	4.20	0.6800		0.29	[0.19; 0.40]	2.9%	1.4%
Wang F, 2023	59	4.38	0.6800	243	4.17	0.6100		0.34	[0.05; 0.62]	0.4%	1.3%
Cui, 2023	750	4.69	0.8400	4122	4.55	0.8200		0.17	[0.09; 0.25]	5.4%	1.5%
Liu, 2023	67	4.25	0.7400	446	4.17	0.6800		0.12	[−0.14; 0.37]	0.5%	1.3%
Li, 2023	100	4.71	1.0600	218	4.86	0.9100		−0.16	[−0.39; 0.08]	0.6%	1.3%
Duo, 2022	300	4.20	0.8200	1043	3.95	0.7300		0.33	[0.20; 0.46]	2.0%	1.4%
Zou, 2023	40	6.09	0.9700	65	5.00	1.1800		0.98	[0.56; 1.40]	0.2%	1.1%
Wang, 2023	162	4.26	0.5200	48	4.20	0.4700		0.12	[−0.20; 0.44]	0.3%	1.2%
Gao, 2023	37	4.68	0.7700	553	4.46	0.7000		0.31	[−0.02; 0.65]	0.3%	1.2%
Mustaniemi, 2023	1040	4.81	0.8000	958	4.63	0.8000		0.22	[0.14; 0.31]	4.2%	1.5%
Cui, 2023	150	6.23	0.7100	150	5.52	1.3000		0.68	[0.44; 0.91]	0.6%	1.3%
Duo, 2023	272	4.10	0.6700	1017	3.90	0.8200		0.25	[0.12; 0.39]	1.8%	1.4%
Ortega−Montiel, 2024	294	4.60	1.0000	1762	4.40	0.9000		0.22	[0.09; 0.34]	2.1%	1.4%
Ma, 2024	201	4.52	0.8100	872	4.51	0.7800		0.01	[−0.14; 0.17]	1.4%	1.4%
Zhao, 2024	261	4.14	0.7900	1327	3.91	0.6700		0.33	[0.20; 0.47]	1.8%	1.4%
Rajeevan, 2024	29	5.64	1.2600	143	5.66	1.2800		−0.02	[−0.41; 0.38]	0.2%	1.1%
Niu, 2024	519	4.61	0.8800	1281	4.65	0.8800		−0.05	[−0.15; 0.06]	3.1%	1.4%
Ma, 2024	103	4.70	1.0800	225	4.86	0.9200		−0.16	[−0.40; 0.07]	0.6%	1.3%
Hou W, 2016	268	4.90	1.0000	474	4.90	0.9000		0.00	[−0.15; 0.15]	1.5%	1.4%
Houde AA, 2013	26	4.75	0.9000	74	4.73	0.7700		0.02	[−0.42; 0.47]	0.2%	1.0%
Pazhohan A, 2017	176	5.23	1.0400	778	5.06	0.8700		0.19	[0.02; 0.35]	1.2%	1.4%
Ren Z, 2020	51	5.45	1.3400	48	5.25	1.4200		0.14	[−0.25; 0.54]	0.2%	1.1%
Ruchat SM, 2013	30	4.73	0.8400	14	4.96	0.9100		−0.26	[−0.90; 0.38]	0.1%	0.8%
Bawah AT, 2019	70	7.20	1.2000	70	5.18	1.5600		1.44	[1.07; 1.82]	0.2%	1.1%
Li J, 2021	80	4.62	0.7900	317	4.42	0.7800		0.26	[0.01; 0.50]	0.5%	1.3%
Lu L, 2022	74	6.62	0.6100	414	5.12	0.5300		2.76	[2.46; 3.06]	0.4%	1.2%
Meyer B, 2023	43	6.60	1.4300	26	6.18	0.9700		0.32	[−0.17; 0.82]	0.1%	1.0%
Song S, 2021	239	4.02	0.5900	843	3.92	0.5500		0.18	[0.03; 0.32]	1.6%	1.4%
Sun J, 2021	144	4.09	0.9000	600	3.93	0.6500		0.23	[0.04; 0.41]	1.0%	1.4%
Wang X, 2022	49	4.46	0.7300	50	4.11	0.6500		0.50	[0.10; 0.90]	0.2%	1.1%
Wang W, 2023	256	5.00	0.9800	2272	4.87	0.9400		0.14	[0.01; 0.27]	2.0%	1.4%
Wani K, 2020	123	5.40	1.4000	375	5.00	1.1000		0.34	[0.13; 0.54]	0.8%	1.4%
Zhao X, 2023	231	4.06	0.6700	1091	3.93	0.6900		0.19	[0.05; 0.33]	1.6%	1.4%
**Fixed effect model**	**17006**			**56447**				**0.20**	**[0.19; 0.22]**	**100.0%**	**--**
**Random effects model**								**0.38**	**[0.30; 0.47]**	**--**	**100.0%**

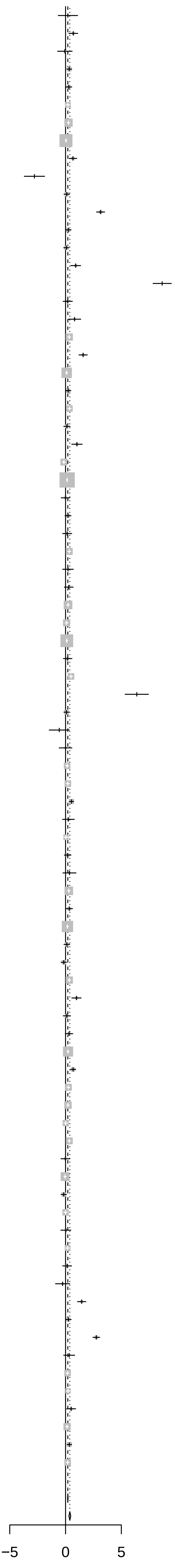

Heterogeneity: $I^2 = 95\%$, $\tau^2 = 0.1239$, $p < 0.01$
