# Supplementary material for: Trimester-Specific Serum Lipid Profiles in Gestational Diabetes Mellitus: A Systematic Review, Meta-Analysis, and Meta-Regression
Source: Medicina (Kaunas). 2025 Jul 17;61(7):1290. doi: 10.3390/medicina61071290 (PMC12300116; doi:10.3390/medicina61071290)

Study	Experimental Total	Experimental Mean	Experimental SD	Control Total	Control Mean	Control SD	SMD	95%−CI	Weight (fixed)	Weight (random)
Montelongo, 1992	9	5.90	1.0500	12	6.02	0.7300	−0.13	[−1.00; 0.73]	0.0%	0.3%
Shelley−Jones, 1993	16	7.38	1.6800	15	7.39	1.7400	−0.01	[−0.71; 0.70]	0.1%	0.3%
Shelley−Jones, 1993	19	5.97	1.2600	15	7.39	1.7400	−0.93	[−1.65; −0.21]	0.1%	0.3%
Clark C, 1997	52	5.30	1.0100	127	5.51	1.1100	−0.19	[−0.52; 0.13]	0.3%	0.4%
Seghieri G, 2003	15	7.30	1.2000	78	6.70	1.1000	0.53	[−0.02; 1.09]	0.1%	0.3%
Toescu V, 2004	12	6.20	0.8000	17	5.95	0.8000	0.30	[−0.44; 1.05]	0.0%	0.3%
Tarim E, 2004	28	6.22	0.9000	210	5.74	1.0800	0.45	[0.05; 0.85]	0.2%	0.4%
Di Cianni G, 2005	36	6.59	0.9000	121	6.34	1.3000	0.20	[−0.17; 0.58]	0.2%	0.4%
Tarim E, 2006	30	6.25	0.8500	40	5.92	1.0600	0.33	[−0.14; 0.81]	0.1%	0.4%
Qui C, 2007	105	4.70	1.1300	96	4.96	0.9800	−0.24	[−0.52; 0.03]	0.3%	0.4%
Altinova A, 2007	34	6.25	0.9900	31	5.92	0.7900	0.36	[−0.13; 0.85]	0.1%	0.4%
Sánchez−Vera I, 2007	62	5.80	1.5500	45	5.00	1.5500	0.51	[0.12; 0.90]	0.2%	0.4%
Molnar J, 2008	17	6.40	1.1200	20	6.30	0.9400	0.10	[−0.55; 0.74]	0.1%	0.3%
Davari−Tanha F, 2008	40	5.58	0.6900	40	5.27	0.8700	0.39	[−0.05; 0.83]	0.1%	0.4%
Idzior−Walus B, 2008	44	6.60	1.0000	17	6.60	1.0000	0.00	[−0.56; 0.56]	0.1%	0.3%
Rizzo M, 2008	27	6.30	0.8000	23	6.00	0.8000	0.37	[−0.19; 0.93]	0.1%	0.3%
McGrowder D, 2009	84	5.71	2.2000	90	5.01	3.0300	0.26	[−0.04; 0.56]	0.3%	0.4%
Vijayalaxmi KD, 2009	25	5.77	1.2400	182	4.60	1.1100	1.04	[0.61; 1.47]	0.1%	0.4%
Kuzmicki M, 2009	81	6.70	1.3300	82	6.10	1.3300	0.45	[0.14; 0.76]	0.3%	0.4%
Habib F, 2009	100	5.31	1.1700	100	5.13	1.2400	0.15	[−0.13; 0.43]	0.3%	0.4%
Su Y, 2010	63	6.00	1.2000	58	6.10	1.1000	−0.09	[−0.44; 0.27]	0.2%	0.4%
Stein S, 2010	40	6.60	2.2000	80	6.30	1.8000	0.15	[−0.23; 0.53]	0.2%	0.4%
Coskun A, 2010	21	4.96	0.7800	24	5.13	0.7100	−0.22	[−0.81; 0.36]	0.1%	0.3%
Santos I, 2010	150	5.90	1.2000	600	5.10	1.3000	0.62	[0.44; 0.81]	0.8%	0.4%
Paradisi G, 2010	12	6.83	1.1800	38	6.46	1.9100	0.21	[−0.44; 0.86]	0.1%	0.3%
Schaefer−Graf U, 2011	150	6.67	1.4700	190	6.56	1.5200	0.07	[−0.14; 0.29]	0.6%	0.4%
Gomathi KG, 2011	26	5.94	0.6900	36	4.70	0.2800	2.48	[1.80; 3.15]	0.1%	0.3%
Caglar G, 2011	19	6.20	1.0300	15	6.00	0.9500	0.20	[−0.48; 0.87]	0.1%	0.3%
Ozuguz U, 2011	61	6.49	1.2100	40	6.02	1.0800	0.40	[0.00; 0.80]	0.2%	0.4%
Zhaoxia L, 2012	28	5.20	1.3000	32	2.90	0.8000	2.14	[1.49; 2.78]	0.1%	0.3%
Winhofer Y, 2010	26	6.85	1.1300	52	6.90	1.1100	−0.04	[−0.52; 0.43]	0.1%	0.4%
Ping F, 2012	488	6.05	1.0200	582	6.14	1.0000	−0.09	[−0.21; 0.03]	1.8%	0.5%
Vural M, 2012	39	5.45	1.2900	40	5.25	0.5900	0.20	[−0.24; 0.64]	0.1%	0.4%
Naf S, 2012	77	6.59	1.0900	130	6.68	1.1000	−0.08	[−0.36; 0.20]	0.3%	0.4%
Baykus Y, 2012	20	6.12	0.9800	20	6.33	1.0800	−0.20	[−0.82; 0.42]	0.1%	0.3%
Alanbay I, 2012	37	6.28	1.0000	42	6.41	0.8700	−0.14	[−0.58; 0.30]	0.1%	0.4%
Rezvan N, 2011	35	5.38	1.0000	35	5.22	0.8300	0.17	[−0.30; 0.64]	0.1%	0.4%
Khan R,2012	103	5.32	0.4900	97	5.04	0.6200	0.50	[0.22; 0.78]	0.3%	0.4%
Gkiomisi A, 2013	44	6.32	1.3900	44	6.92	1.2600	−0.45	[−0.87; −0.03]	0.1%	0.4%
Atay A, 2013	65	5.72	0.8300	66	4.09	0.6500	2.18	[1.74; 2.61]	0.1%	0.4%
dos Santos−Weiss I, 2012	288	5.60	1.2000	288	5.90	1.2000	−0.25	[−0.41; −0.09]	1.0%	0.4%
Todoric J, 2013	64	6.38	1.2000	165	6.75	1.0300	−0.34	[−0.63; −0.05]	0.3%	0.4%
Wang D, 2013	30	6.48	1.0100	60	6.16	0.8600	0.35	[−0.09; 0.79]	0.1%	0.4%
Kuzmicki M, 2014	130	6.50	1.1100	140	4.40	1.1100	1.89	[1.60; 2.17]	0.3%	0.4%
Atay A, 2014	68	3.67	0.3300	73	3.57	0.2800	0.33	[−0.01; 0.66]	0.2%	0.4%
Javadian P, 2013	52	5.22	2.0900	50	6.72	2.0100	−0.73	[−1.13; −0.32]	0.2%	0.4%
Ebert T, 2014	74	6.71	1.7400	74	6.31	1.8400	0.22	[−0.10; 0.55]	0.3%	0.4%
Liang Zhaoxia, 2014	35	4.40	1.0300	35	3.90	0.5100	0.61	[0.13; 1.09]	0.1%	0.4%
Bullon, 2013	26	6.26	1.5400	162	2.58	0.4700	5.13	[4.46; 5.80]	0.1%	0.3%
Guimarães, 2014	150	5.86	1.2300	295	2.28	0.6000	4.13	[3.80; 4.47]	0.2%	0.4%
Houde, 2014	27	5.90	0.7500	99	6.26	1.1200	−0.34	[−0.77; 0.09]	0.1%	0.4%
Atay, 2013	37	5.70	0.8200	38	4.09	0.6500	2.16	[1.58; 2.73]	0.1%	0.3%
Wei, 2014	76	6.14	0.9300	86	5.02	0.7700	1.31	[0.97; 1.65]	0.2%	0.4%
Wei, 2014	37	5.50	1.0000	26	6.30	0.9000	−0.82	[−1.35; −0.30]	0.1%	0.4%
Reyes Lopez	90	5.30	0.9800	108	5.30	0.9900	0.00	[−0.28; 0.28]	0.3%	0.4%
Hesham, 2015	112	4.55	1.2700	218	3.80	2.0700	0.41	[0.18; 0.64]	0.5%	0.4%
Beigi, 2015	40	5.71	1.0400	40	5.70	0.7700	0.01	[−0.43; 0.45]	0.1%	0.4%
Trebotic, 2015	21	7.00	1.6000	19	6.00	1.1000	0.71	[0.07; 1.35]	0.1%	0.3%
Telejko, 2015	49	5.80	1.4100	30	6.00	1.7800	−0.13	[−0.58; 0.33]	0.1%	0.4%
Lehmann, 2015	9	6.00	1.1700	15	6.20	0.7700	−0.21	[−1.04; 0.62]	0.0%	0.3%
Simon Muela, 2015	66	6.52	1.0600	71	6.77	1.0900	−0.23	[−0.57; 0.11]	0.2%	0.4%
Altinova A, 2015	30	6.35	1.2400	35	6.18	1.7600	0.11	[−0.38; 0.60]	0.1%	0.4%
De Melo SF, 2015	200	5.94	1.2700	200	4.92	1.3200	0.79	[0.58; 0.99]	0.6%	0.4%
Takhshid MA, 2015	70	6.03	1.2900	70	6.21	1.3200	−0.14	[−0.47; 0.19]	0.2%	0.4%
Takhshid MA, 2015	65	5.71	1.2900	70	6.21	1.3700	−0.37	[−0.71; −0.03]	0.2%	0.4%
Wurst U, 2015	74	6.71	1.7400	74	6.31	1.8400	0.22	[−0.10; 0.55]	0.3%	0.4%
Li XM, 2015	16	5.73	0.7200	15	5.31	1.0500	0.46	[−0.26; 1.17]	0.1%	0.3%
Li XM, 2015	16	4.75	1.0400	15	5.49	1.0100	−0.70	[−1.43; 0.03]	0.0%	0.3%
Li XM, 2015	16	4.91	0.8400	15	5.24	1.4300	−0.28	[−0.98; 0.43]	0.1%	0.3%
Korkmazer E, 2015	39	6.08	1.1100	40	6.31	1.2700	−0.19	[−0.63; 0.25]	0.1%	0.4%
Jia X, 2014	86	5.40	1.1800	92	6.14	1.1300	−0.64	[−0.94; −0.34]	0.3%	0.4%
Iyidir OT, 2014	26	6.71	1.3400	24	6.00	1.4600	0.50	[−0.06; 1.06]	0.1%	0.3%
Demirpençe M, 2016	20	5.83	1.1900	11	6.80	1.8100	−0.66	[−1.41; 0.10]	0.0%	0.3%
Zhang Y, 2016	40	5.52	1.0800	240	5.56	0.9700	−0.04	[−0.38; 0.29]	0.2%	0.4%
Edu A, 2016	13	5.84	0.9900	96	5.85	1.2900	−0.01	[−0.59; 0.57]	0.1%	0.3%
Ertug EY, 2016	29	6.05	1.1900	20	6.23	1.4000	−0.14	[−0.71; 0.43]	0.1%	0.3%
Mou Y, 2016	40	4.74	0.5500	40	3.90	0.6000	1.45	[0.95; 1.94]	0.1%	0.4%
Zheng D, 2016	50	4.45	0.7500	50	4.41	0.7100	0.05	[−0.34; 0.45]	0.2%	0.4%
Khosrowbeygi A, 2015	30	6.07	1.6400	30	6.62	1.7500	−0.32	[−0.83; 0.19]	0.1%	0.4%
Aydemir B, 2015	116	5.28	0.8600	120	4.76	0.6300	0.69	[0.43; 0.95]	0.4%	0.4%
Qiu YH, 2016	223	6.02	1.2000	265	6.20	1.1800	−0.15	[−0.33; 0.03]	0.8%	0.4%
Ye D, 2016	556	6.20	1.0000	500	6.10	1.0000	0.10	[−0.02; 0.22]	1.8%	0.5%
Mac−Marcjanek K, 2017	104	6.23	1.0500	41	6.52	0.8400	−0.29	[−0.65; 0.07]	0.2%	0.4%
Tu WJ, 2017	135	4.98	0.6400	1015	4.70	0.6100	0.46	[0.28; 0.64]	0.8%	0.4%
Zhou X, 2017	180	5.85	0.7800	60	5.82	0.8100	0.04	[−0.25; 0.33]	0.3%	0.4%
del Mar Roca−Rodríguez M, 2017	63	13.10	2.5000	63	14.11	2.4900	−0.40	[−0.76; −0.05]	0.2%	0.4%
Saucedo R, 2017	80	6.13	1.0200	80	5.96	0.7700	0.19	[−0.12; 0.50]	0.3%	0.4%
Li L, 2017	200	6.33	1.4600	410	6.48	1.3100	−0.11	[−0.28; 0.06]	0.9%	0.4%
Mousavi SN, 2017	200	5.19	1.2000	200	5.36	1.1000	−0.15	[−0.34; 0.05]	0.7%	0.4%
Ersoy GS, 2016	62	6.59	1.2900	73	6.25	6.5900	0.07	[−0.27; 0.41]	0.2%	0.4%
Jameshorani M, 2018	65	5.11	1.3600	65	5.36	1.1000	−0.20	[−0.55; 0.14]	0.2%	0.4%
Yang Y, 2016	209	5.69	1.0700	215	5.16	1.1100	0.49	[0.29; 0.68]	0.7%	0.4%
Barat S, 2018	250	5.93	1.0700	87	6.07	3.4200	−0.07	[−0.32; 0.17]	0.4%	0.4%
Yuan X, 2018	86	5.37	0.8800	273	5.56	0.8900	−0.21	[−0.46; 0.03]	0.4%	0.4%
Eken MK, 2018	63	6.70	1.1000	64	6.40	1.4000	0.24	[−0.11; 0.59]	0.2%	0.4%
Bukowiecka−Matusiak M, 2018	32	6.70	0.9600	11	5.70	1.0400	1.00	[0.28; 1.72]	0.1%	0.3%
Zhang L, 2018	77	4.57	1.0100	154	4.42	0.8400	0.17	[−0.11; 0.44]	0.4%	0.4%
Bagci H, 2018	40	5.94	1.0800	40	5.35	1.0100	0.56	[0.11; 1.01]	0.1%	0.4%
Khosrowbeygi A, 2018	40	5.84	1.6400	40	6.28	1.6400	−0.27	[−0.71; 0.17]	0.1%	0.4%
Franzago M, 2018	104	6.80	1.2900	124	6.21	1.4000	0.44	[0.17; 0.70]	0.4%	0.4%
Al−Ajlan A, 2018	116	5.10	1.0000	303	4.90	1.0000	0.20	[−0.01; 0.41]	0.6%	0.4%
Al−Daghri NM, 2018	63	5.10	1.0000	54	4.80	1.1000	0.28	[−0.08; 0.65]	0.2%	0.4%
Bao W, 2018	107	5.39	1.5500	214	5.49	1.7600	−0.06	[−0.29; 0.17]	0.5%	0.4%
Siddiqui K, 2017	14	6.32	1.1100	21	6.52	1.2400	−0.16	[−0.84; 0.51]	0.1%	0.3%
Cao W, 2018	33	3.40	1.1500	33	2.47	0.1100	1.13	[0.60; 1.65]	0.1%	0.4%
Yue CY, 2018	88	6.20	0.8400	456	6.17	1.2800	0.02	[−0.20; 0.25]	0.5%	0.4%
Siddiqui K, 2019	44	5.45	1.0100	48	5.59	0.7800	−0.15	[−0.56; 0.25]	0.2%	0.4%
Demi E, 2019	85	5.76	1.4200	90	5.58	1.2400	0.13	[−0.16; 0.43]	0.3%	0.4%
Li S, 2018	90	6.13	0.9800	90	6.03	1.0200	0.10	[−0.19; 0.39]	0.3%	0.4%
de la Torre NG, 2019	130	6.32	1.0900	802	6.19	1.1200	0.12	[−0.07; 0.30]	0.8%	0.4%
Pezeshki B, 2019	30	3.59	0.8800	301	3.79	0.4500	−0.40	[−0.77; −0.02]	0.2%	0.4%
Wu, 2019	40	5.72	0.9000	40	5.70	0.8300	0.02	[−0.42; 0.46]	0.1%	0.4%
Kang, 2019	72	4.78	0.9900	100	4.54	0.8300	0.27	[−0.04; 0.57]	0.3%	0.4%
Alhabri, 2019	200	5.80	1.2000	200	5.20	1.1000	0.52	[0.32; 0.72]	0.7%	0.4%
Anjum, 2019	25	4.79	0.5900	50	5.11	1.0500	−0.34	[−0.83; 0.14]	0.1%	0.4%
Layton, 2018	67	6.23	1.6200	739	6.18	1.0400	0.05	[−0.20; 0.30]	0.4%	0.4%
Wang, 2019	300	5.65	1.0000	1283	5.13	0.9400	0.55	[0.42; 0.67]	1.6%	0.5%
Abo−Elmatty, 2018	85	7.00	1.0500	80	3.47	0.5200	4.20	[3.65; 4.75]	0.1%	0.4%
Abo−Elmatty, 2018	85	6.91	0.9200	80	5.76	1.0700	1.15	[0.82; 1.48]	0.2%	0.4%
Alyas S, 2019	58	9.52	0.4600	100	8.81	0.4000	1.67	[1.30; 2.04]	0.2%	0.4%
Benhalima K, 2019	189	6.30	1.0400	1113	6.30	1.0400	0.00	[−0.15; 0.15]	1.1%	0.4%
Wang F, 2019	100	7.35	0.9000	100	5.32	0.4900	2.79	[2.40; 3.18]	0.2%	0.4%
Abd KI, 2020	45	5.65	1.1100	45	4.59	0.8000	1.09	[0.64; 1.53]	0.1%	0.4%
Bulmus F, 2020	30	5.92	1.0400	30	5.83	1.3200	0.07	[−0.43; 0.58]	0.1%	0.4%
Khosrowbeygi A, 2020	49	5.70	1.6100	51	6.19	1.5700	−0.31	[−0.70; 0.09]	0.2%	0.4%
Liu Y, 2020	30	6.12	1.0600	30	6.37	1.2700	−0.21	[−0.72; 0.30]	0.1%	0.4%
Mosavat M, 2020	53	5.81	1.0200	43	6.11	0.9200	−0.30	[−0.71; 0.10]	0.2%	0.4%
Mohammed Ali D, 2020	60	5.75	0.5300	30	5.18	0.5400	1.06	[0.59; 1.53]	0.1%	0.4%
Wei L, 2020	241	5.64	1.1100	391	5.87	1.1800	−0.20	[−0.36; −0.04]	1.0%	0.4%
Sun M, 2020	64	6.32	0.4500	128	5.18	0.6200	1.99	[1.63; 2.36]	0.2%	0.4%
Yavuzkir S, 2020	30	5.36	1.2000	30	4.98	0.8600	0.36	[−0.15; 0.87]	0.1%	0.4%
Zhong L, 2020	40	5.75	1.1400	40	5.59	0.9600	0.15	[−0.29; 0.59]	0.1%	0.4%
Liu L, 2020	61	4.78	3.9200	122	4.42	1.0800	0.15	[−0.16; 0.46]	0.3%	0.4%
Contreras−Duarte S, 2020	69	5.87	1.2500	41	6.16	0.9600	−0.25	[−0.64; 0.14]	0.2%	0.4%
Contreras−Duarte S, 2020	48	5.22	0.8900	41	6.16	0.9600	−1.01	[−1.45; −0.57]	0.1%	0.4%
Gülcü Bulmus F, 2020	30	5.92	1.0500	30	5.85	1.3300	0.06	[−0.45; 0.56]	0.1%	0.4%
O’Malley E, 2020	108	6.00	1.3000	94	6.10	1.3000	−0.08	[−0.35; 0.20]	0.3%	0.4%
Liu Y, 2020	110	6.44	1.0700	100	3.33	0.9400	3.07	[2.67; 3.47]	0.2%	0.4%
Liu T, 2020	300	4.37	0.4900	261	4.52	0.8300	−0.22	[−0.39; −0.06]	1.0%	0.4%
Lu W, 2021	199	5.83	1.1100	199	6.13	1.0500	−0.28	[−0.47; −0.08]	0.7%	0.4%
Mei J, 2021	104	5.66	0.3700	104	4.87	0.2200	2.59	[2.22; 2.96]	0.2%	0.4%
Meng X, 2021	34	5.65	1.0500	34	6.04	0.7900	−0.41	[−0.90; 0.07]	0.1%	0.4%
Sudana N, 2021	25	5.53	0.2800	50	4.27	0.5900	2.45	[1.82; 3.07]	0.1%	0.3%
Raczkowska B, 2021	26	1.96	0.6900	29	6.00	2.1000	−2.49	[−3.21; −1.78]	0.1%	0.3%
Ramasammy R, 2021	210	5.19	0.8100	209	4.76	0.5700	0.61	[0.42; 0.81]	0.7%	0.4%
Ramanjaneya M, 2021a	34	4.90	1.2000	16	5.20	1.3000	−0.24	[−0.84; 0.36]	0.1%	0.3%
Ramanjaneya M, 2021b	46	4.90	1.1000	26	5.00	1.1000	−0.09	[−0.57; 0.39]	0.1%	0.4%
Kapustin V, 2021	88	6.60	0.3000	38	6.53	0.3900	0.21	[−0.17; 0.59]	0.2%	0.4%
Kapustin V, 2021	42	6.32	0.6000	38	6.53	0.3900	−0.41	[−0.85; 0.04]	0.1%	0.4%
Zhang L, 2021	36	5.24	0.9800	38	5.44	0.8700	−0.21	[−0.67; 0.24]	0.1%	0.4%
Rakchna M, 2021	73	5.75	0.8500	72	5.29	0.7800	0.56	[0.23; 0.89]	0.2%	0.4%
Kang M, 2021	116	5.10	0.9100	297	5.11	0.8600	−0.01	[−0.23; 0.20]	0.6%	0.4%
Baryla I, 2021	98	6.60	1.1300	37	6.87	1.2000	−0.23	[−0.61; 0.15]	0.2%	0.4%
Abd−Eltawab A, 2020	109	5.46	1.2100	103	4.86	1.1300	0.51	[0.24; 0.78]	0.4%	0.4%
Ren M, 2021	56	4.64	0.4900	56	3.29	0.4300	2.91	[2.37; 3.44]	0.1%	0.4%
Xu D, 2021	80	5.51	1.3200	80	5.59	1.8700	−0.05	[−0.36; 0.26]	0.3%	0.4%
Benhalima K, 2021	97	6.20	0.9600	327	6.40	1.1100	−0.19	[−0.41; 0.04]	0.5%	0.4%
Tabacu C, 2021	16	6.44	1.4100	83	5.37	1.0700	0.94	[0.39; 1.49]	0.1%	0.4%
Eppel D, 2021	27	5.99	1.0400	40	5.53	0.8400	0.49	[0.00; 0.99]	0.1%	0.4%
Onat T, 2021	60	4.97	0.5800	52	5.11	0.6800	−0.22	[−0.59; 0.15]	0.2%	0.4%
Wang Y, 2021	855	6.30	1.2000	785	5.80	1.1000	0.43	[0.34; 0.53]	2.8%	0.5%
Bulut A, 2021	22	6.57	0.9500	29	6.54	1.1900	0.03	[−0.53; 0.58]	0.1%	0.3%
Yu X, 2021	573	5.37	1.1600	740	5.24	1.0700	0.12	[0.01; 0.23]	2.2%	0.5%
Shalabi T, 2021	114	3.96	0.3000	114	3.56	0.4000	1.13	[0.85; 1.41]	0.3%	0.4%
Adiga U, 2021	100	5.74	1.2300	100	5.97	1.1800	−0.19	[−0.47; 0.09]	0.3%	0.4%
Cheng E, 2022	163	5.17	0.8700	145	4.98	1.8800	0.13	[−0.09; 0.36]	0.5%	0.4%
Huang G, 2022	566	5.37	1.1600	710	5.27	1.0500	0.09	[−0.02; 0.20]	2.2%	0.5%
Juchnicka I, 2022	24	6.16	1.3300	24	6.92	1.6200	−0.50	[−1.08; 0.07]	0.1%	0.3%
Obaidullah M, 2021	132	5.47	1.0500	100	5.00	1.5500	0.36	[0.10; 0.63]	0.4%	0.4%
Adiga U, 2022	74	5.83	1.0100	132	5.91	1.3600	−0.06	[−0.35; 0.22]	0.3%	0.4%
Shen L, 2023	233	6.35	1.2000	1001	6.33	1.0600	0.02	[−0.12; 0.16]	1.3%	0.4%
Sahoo D, 2022	20	5.16	0.2800	45	5.10	0.2100	0.25	[−0.27; 0.78]	0.1%	0.4%
Khan S, 2022	50	5.31	1.3200	226	5.24	0.9600	0.07	[−0.24; 0.37]	0.3%	0.4%
Arslan A, 2022	40	5.83	1.0600	37	5.95	1.5000	−0.09	[−0.54; 0.36]	0.1%	0.4%
Hu W, 2022	315	4.70	0.8100	1876	4.71	0.7700	−0.01	[−0.13; 0.11]	1.9%	0.5%
Wu Y, 2022	130	5.46	0.8700	170	5.43	1.1000	0.03	[−0.20; 0.26]	0.5%	0.4%
Cibickova L, 2022	43	6.10	1.0100	19	6.30	1.0300	−0.19	[−0.74; 0.35]	0.1%	0.4%
Karagoz ZK, 2022	30	5.73	0.4300	30	5.10	0.4600	1.40	[0.83; 1.96]	0.1%	0.3%
Wang, Y 2022	232	6.03	1.0800	696	6.25	1.0500	−0.21	[−0.36; −0.06]	1.2%	0.4%
Jia X, 2022	62	4.63	0.8300	58	4.48	0.7200	0.19	[−0.17; 0.55]	0.2%	0.4%
Zeljkovic A, 2022	15	6.77	1.4800	48	6.77	1.3500	0.00	[−0.58; 0.58]	0.1%	0.3%
Wang J, 2023	47	4.46	0.7400	47	4.12	0.6500	0.48	[0.07; 0.89]	0.2%	0.4%
Ma, 2023	110	6.14	1.2700	110	6.15	1.4400	−0.01	[−0.27; 0.26]	0.4%	0.4%
Akash, 2023	400	4.71	0.9300	100	3.70	0.9100	1.09	[0.86; 1.32]	0.5%	0.4%
Hou, 2021	189	5.86	0.7500	100	5.82	0.7300	0.05	[−0.19; 0.30]	0.4%	0.4%
Kagitci, 2023	30	6.42	1.4600	30	6.62	1.2200	−0.15	[−0.65; 0.36]	0.1%	0.4%
Gawlik, 2023	74	6.73	1.1500	32	6.81	1.0000	−0.07	[−0.49; 0.34]	0.2%	0.4%
Burzynska−Pedziwiatr, 2023	32	6.62	1.2500	11	5.68	0.9700	0.78	[0.07; 1.48]	0.1%	0.3%
Štambuk, 2023	41	6.45	1.2400	48	6.52	1.5900	−0.05	[−0.47; 0.37]	0.2%	0.4%
Shi, 2023	1632	6.13	1.0400	9067	5.59	0.9600	0.56	[0.50; 0.61]	9.3%	0.5%
Li, 2023	148	4.89	1.0800	296	4.87	1.1600	0.02	[−0.18; 0.21]	0.7%	0.4%
Ali Khan, 2023	90	5.39	1.1400	90	5.70	1.2700	−0.26	[−0.55; 0.04]	0.3%	0.4%
Linares−Pineda, 2023	16	6.69	1.3200	16	7.03	1.2400	−0.26	[−0.96; 0.44]	0.1%	0.3%
Shen, 2024	12	6.57	1.0700	10	6.00	1.2700	0.47	[−0.38; 1.32]	0.0%	0.3%
Thilak, 2024	60	5.79	3.2800	60	3.89	0.6800	0.80	[0.42; 1.17]	0.2%	0.4%
Ortega−Montiel, 2024	294	5.60	1.1000	1762	5.50	2.2000	0.05	[−0.08; 0.17]	1.7%	0.5%
Ortega−Montiel, 2024	158	5.70	1.2000	1762	5.50	2.2000	0.09	[−0.07; 0.26]	1.0%	0.4%
Ortega−Montiel, 2024	101	5.50	0.9000	1762	5.50	2.2000	0.00	[−0.20; 0.20]	0.7%	0.4%
Ortega−Montiel, 2024	35	5.10	1.0000	1762	5.50	2.2000	−0.18	[−0.52; 0.15]	0.2%	0.4%
Özer, 2024	44	5.68	1.0500	44	6.08	1.1800	−0.36	[−0.78; 0.07]	0.1%	0.4%
Ghaneialvar, 2024	40	4.48	0.6500	40	4.33	0.6000	0.24	[−0.20; 0.68]	0.1%	0.4%
Aziz, 2024	50	3.70	0.1100	50	3.38	0.0800	3.30	[2.69; 3.91]	0.1%	0.3%
Uysal, 2024	52	5.89	1.1800	31	5.82	1.1300	0.06	[−0.39; 0.50]	0.1%	0.4%
Wu, 2024	35	6.30	1.2600	70	6.19	0.8300	0.11	[−0.30; 0.52]	0.2%	0.4%
Li, 2024	1323	4.52	0.8100	1421	4.52	0.8100	0.00	[−0.07; 0.07]	4.7%	0.5%
Ademoglu EN, 2015	47	6.10	1.3000	32	6.30	1.2000	−0.16	[−0.61; 0.29]	0.1%	0.4%
Anghebem−Oliveira MI, 2017	127	5.81	1.1800	125	5.54	1.2800	0.22	[−0.03; 0.47]	0.4%	0.4%
Boghossian NS, 2017	93	5.84	1.1000	136	6.23	0.9900	−0.38	[−0.64; −0.11]	0.4%	0.4%
Dudzik D, 2014	20	6.94	0.9200	20	6.30	0.9500	0.67	[0.03; 1.31]	0.1%	0.3%
Dudzik D, 2017	24	6.63	0.7400	24	6.44	1.1200	0.20	[−0.37; 0.76]	0.1%	0.3%
Yorulmaz Ertug E, 2016	29	6.06	1.1900	20	6.23	1.1700	−0.14	[−0.71; 0.43]	0.1%	0.3%
Guimarães LO,2014	150	5.85	1.2300	295	5.21	1.3800	0.48	[0.28; 0.68]	0.7%	0.4%
Inegol Gumus I, 2013	34	4.73	1.0100	50	3.99	1.0500	0.71	[0.26; 1.16]	0.1%	0.4%
Hollingsworth DR, 1982	5	6.20	0.6200	21	5.44	0.4500	1.53	[0.45; 2.60]	0.0%	0.2%
Huang Y, 2018	88	4.80	0.8700	386	4.73	0.8200	0.08	[−0.15; 0.32]	0.5%	0.4%
Knopp RH, 1980	22	5.41	1.1900	38	5.46	0.7200	−0.05	[−0.58; 0.47]	0.1%	0.4%
Liang Z, 2015	38	5.90	2.7000	35	4.80	2.0000	0.46	[−0.01; 0.92]	0.1%	0.4%
Liu B, 2016	276	6.09	1.0200	1270	6.11	0.9900	−0.02	[−0.15; 0.11]	1.6%	0.5%
Liu B, 2016	28	6.01	1.3000	25	5.95	1.0900	0.05	[−0.49; 0.59]	0.1%	0.4%
Rojas I, 2002	38	6.42	1.2500	38	6.23	1.3700	0.14	[−0.31; 0.59]	0.1%	0.4%
Whyte K, 2013	22	5.31	0.9700	167	5.08	0.8900	0.25	[−0.19; 0.70]	0.1%	0.4%
Anderlová K, 2022	36	6.30	1.2600	22	7.47	1.5000	−0.85	[−1.41; −0.30]	0.1%	0.3%
Attique H, 2022	37	5.86	1.1500	47	5.31	1.0900	0.49	[0.05; 0.93]	0.1%	0.4%
Beunen K, 2014	231	5.80	1.2000	1612	5.40	1.1000	0.36	[0.22; 0.50]	1.4%	0.4%
Chen Y, 2017	28	5.77	0.9400	56	6.23	1.1000	−0.43	[−0.89; 0.02]	0.1%	0.4%
Diboun I, 2020	32	4.70	0.8000	21	5.00	1.0000	−0.33	[−0.89; 0.22]	0.1%	0.3%
Franzago M, 2020	50	6.00	1.1000	50	5.20	1.0000	0.76	[0.35; 1.16]	0.2%	0.4%
Guan L, 2021	461	5.98	1.3100	626	6.02	1.1800	−0.03	[−0.15; 0.09]	1.8%	0.5%
Guo Y, 2020	87	4.60	0.7000	88	4.50	0.7000	0.14	[−0.15; 0.44]	0.3%	0.4%
Huo Y, 2019	30	5.52	0.8700	28	5.85	0.7800	−0.39	[−0.91; 0.13]	0.1%	0.4%
Iimura Y, 2015	53	6.54	1.1000	1108	6.63	1.0000	−0.09	[−0.37; 0.19]	0.3%	0.4%
Kralisch S, 2009	40	6.60	2.2000	80	6.30	1.8000	0.15	[−0.23; 0.53]	0.2%	0.4%
Li J, 2015	35	4.90	1.7000	43	4.30	1.1000	0.42	[−0.03; 0.88]	0.1%	0.4%
Li J, 2017	100	5.53	1.0700	100	5.02	1.0500	0.48	[0.20; 0.76]	0.3%	0.4%
Li Y, 2021	359	5.42	0.9200	273	5.56	0.9200	−0.15	[−0.31; 0.01]	1.1%	0.4%
Lu L, 2023	83	5.85	1.4300	649	4.15	1.1800	1.40	[1.16; 1.64]	0.5%	0.4%
Meyer B, 2023	43	6.56	1.7600	26	6.81	1.1700	−0.16	[−0.65; 0.33]	0.1%	0.4%
Onat T, 2021	40	4.82	1.2100	40	4.74	1.1400	0.07	[−0.37; 0.51]	0.1%	0.4%
Qin L, 2023	82	5.75	1.1000	89	6.04	1.0000	−0.28	[−0.58; 0.03]	0.3%	0.4%
Sun J, 2022	80	6.12	1.0700	79	5.89	0.8900	0.23	[−0.08; 0.54]	0.3%	0.4%
Takhshid M, 2015	75	5.97	1.3100	70	6.21	1.3200	−0.18	[−0.51; 0.14]	0.2%	0.4%
Theodorakopoulou M, 2021	21	6.50	1.1900	15	6.70	1.1100	−0.17	[−0.83; 0.49]	0.1%	0.3%
Tönjes A, 2019	74	6.73	1.3400	74	6.49	1.3800	0.18	[−0.15; 0.50]	0.3%	0.4%
Torun G, 2023	50	3.29	0.6300	50	2.82	0.5800	0.77	[0.36; 1.18]	0.2%	0.4%
Tutar D, 2022	40	5.85	1.2100	40	6.29	0.9800	−0.40	[−0.84; 0.05]	0.1%	0.4%
Wang X, 2015	839	6.06	1.0700	900	6.10	1.0100	−0.04	[−0.13; 0.06]	3.0%	0.5%
Wang H, 2019	35	5.91	1.0200	35	5.58	0.7100	0.37	[−0.10; 0.84]	0.1%	0.4%
Wang H, 2019	35	6.22	1.0700	35	5.92	0.7300	0.32	[−0.15; 0.80]	0.1%	0.4%
Wójcik M, 2015	77	6.98	1.0000	34	6.68	1.2000	0.28	[−0.13; 0.69]	0.2%	0.4%
Xu L, 2013	120	5.95	1.1600	60	5.43	0.8700	0.48	[0.17; 0.80]	0.3%	0.4%
Xu M, 2015	55	5.83	1.2000	66	5.86	1.0200	−0.03	[−0.38; 0.33]	0.2%	0.4%
Yakout S, 2023	46	5.35	1.2000	77	4.70	1.0000	0.60	[0.23; 0.97]	0.2%	0.4%
Yi J, 2018	141	6.00	0.7600	230	6.17	0.9500	−0.19	[−0.40; 0.02]	0.6%	0.4%
Zhang Y, 2015	186	5.12	2.4600	330	4.86	1.5600	0.13	[−0.05; 0.31]	0.8%	0.4%
Zhang H, 2022	110	5.47	0.9600	416	5.31	0.9500	0.17	[−0.04; 0.38]	0.6%	0.4%
**Fixed effect model**	**26189**			**56400**			**0.20**	**[0.19; 0.22]**	**100.0%**	**−−**
**Random effects model**							**0.27**	**[0.20; 0.33]**	**−−**	**100.0%**

Heterogeneity: $I^2 = 94\%$, $\tau^2 = 0.2576$, $p = 0$

Standardised Mean Difference

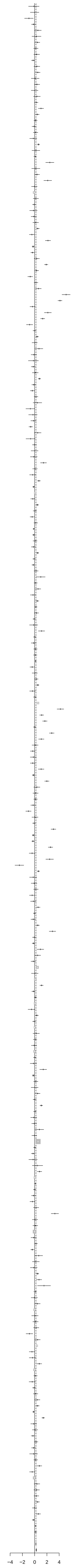

Supplement: Supplementary file 1 [file medicina-61-01290-s001.zip › Figure S23 Total cholesterol 2nd trimester.pdf]
