# Supplementary material for: Trimester-Specific Serum Lipid Profiles in Gestational Diabetes Mellitus: A Systematic Review, Meta-Analysis, and Meta-Regression
Source: Medicina (Kaunas). 2025 Jul 17;61(7):1290. doi: 10.3390/medicina61071290 (PMC12300116; doi:10.3390/medicina61071290)
Supplement: Supplementary file 1 [file medicina-61-01290-s001.zip › Figure S24 Total cholesterol 3rd trimester.pdf]

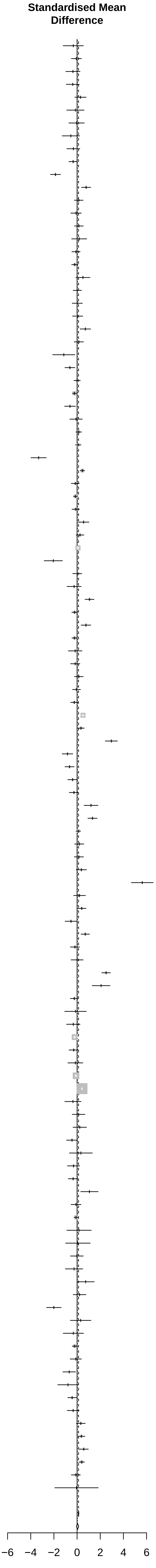

Study	Experimental Total	Experimental Mean	Experimental SD	Control Total	Control Mean	Control SD	SMD	95%−CI	Weight (fixed)	Weight (random)
Montelongo, 1992	9	6.30	0.9900	12.000	6.69	1.2500	−0.33	[−1.20; 0.54]	0.1%	0.6%
Meyer B, 1996	44	6.03	1.1900	36.000	6.10	1.3300	−0.06	[−0.50; 0.39]	0.4%	0.9%
Koukkou E, 1997	20	6.23	1.6000	22.000	6.71	1.0000	−0.36	[−0.97; 0.25]	0.2%	0.8%
Couch S, 1998	25	5.13	1.0800	25.000	5.57	1.2500	−0.37	[−0.93; 0.19]	0.2%	0.8%
Bartha J, 2000	34	6.39	1.0800	32.000	6.08	1.0100	0.29	[−0.19; 0.78]	0.3%	0.9%
Paradisi G, 2002	13	6.21	0.4300	15.000	6.32	0.9300	−0.14	[−0.89; 0.60]	0.1%	0.7%
Vitoratos G, 2002	15	7.70	2.5000	21.000	7.80	2.1400	−0.04	[−0.71; 0.62]	0.2%	0.8%
Toescu V, 2004	12	6.30	0.9000	17.000	6.90	1.2000	−0.54	[−1.29; 0.22]	0.1%	0.7%
Ranheim T, 2004	22	6.50	1.4100	29.000	7.00	1.6200	−0.32	[−0.88; 0.24]	0.2%	0.8%
Tsai P, 2005	34	6.40	0.9000	219.000	6.80	1.2000	−0.34	[−0.70; 0.02]	0.5%	1.0%
Grissa O, 2007	59	5.37	0.6500	60.000	6.33	0.3200	−1.87	[−2.30; −1.43]	0.4%	0.9%
Sánchez−Vera I, 2007	62	7.20	1.6500	45.000	5.90	1.6500	0.78	[0.38; 1.18]	0.4%	1.0%
Szymanska M, 2008	81	6.50	0.8100	41.000	6.36	1.2200	0.14	[−0.23; 0.52]	0.5%	1.0%
Akturk M, 2008	47	6.41	0.8200	31.000	6.50	1.3400	−0.08	[−0.54; 0.37]	0.3%	0.9%
Pfau D, 2010	40	6.60	2.2000	80.000	6.30	1.8000	0.15	[−0.23; 0.53]	0.5%	1.0%
Paradisi G, 2010	12	7.33	1.2800	38.000	6.98	2.0300	0.18	[−0.47; 0.83]	0.2%	0.8%
Akturk M, 2010	54	6.36	0.8800	69.000	6.46	1.2500	−0.09	[−0.45; 0.27]	0.5%	1.0%
Retnakaran R, 2010	136	6.21	1.2000	87.000	6.45	1.2500	−0.20	[−0.47; 0.07]	1.0%	1.0%
Culha C, 2011	24	6.21	0.4900	20.000	5.88	0.7800	0.51	[−0.10; 1.11]	0.2%	0.8%
Saucedo R, 2011	60	6.89	1.3700	60.000	6.86	1.5700	0.02	[−0.34; 0.38]	0.5%	1.0%
Giannubilo S, 2011	40	7.16	0.5600	40.000	7.15	0.4100	0.02	[−0.42; 0.46]	0.4%	0.9%
Giannubilo S, 2011	40	7.21	0.4500	40.000	7.19	0.3200	0.05	[−0.39; 0.49]	0.4%	0.9%
Giannubilo S, 2011	40	7.86	0.9100	40.000	7.22	0.8600	0.72	[0.26; 1.17]	0.3%	0.9%
Ghafoor S, 2012	46	5.09	1.9000	50.000	4.83	1.4800	0.15	[−0.25; 0.55]	0.4%	1.0%
Farhan S, 2012	10	5.89	0.9800	10.000	7.16	1.1400	−1.14	[−2.11; −0.18]	0.1%	0.6%
Gkiomisi A, 2013	44	6.45	1.1900	44.000	7.24	1.3300	−0.62	[−1.05; −0.19]	0.4%	0.9%
Khan R, 2013	103	5.30	0.4900	97.000	5.30	0.4900	0.00	[−0.28; 0.28]	0.9%	1.0%
dos Santos−Weiss I, 2012	288	5.90	1.3000	288.000	6.20	1.3000	−0.23	[−0.39; −0.07]	2.6%	1.1%
Kärkkäinen H, 2013	42	6.22	0.2300	32.000	6.37	0.2600	−0.61	[−1.08; −0.14]	0.3%	0.9%
Agakidou E, 2013	27	6.94	1.2300	27.000	7.05	1.2000	−0.09	[−0.62; 0.44]	0.2%	0.9%
Eslamian R, 2013	112	5.66	0.8700	159.000	5.56	0.5600	0.14	[−0.10; 0.38]	1.2%	1.1%
Eslamian R, 2013	112	6.23	0.7500	159.000	6.15	0.9000	0.09	[−0.15; 0.34]	1.2%	1.1%
Yousefzadeh G, 2013	60	2.56	0.5100	30.000	5.66	1.4400	−3.32	[−3.98; −2.66]	0.2%	0.8%
Al−Hakeem M, 2014	200	5.70	1.2000	300.000	5.20	1.0000	0.46	[0.28; 0.64]	2.1%	1.1%
Wójcik M, 2014	132	6.46	1.4700	43.000	6.67	1.0100	−0.15	[−0.50; 0.19]	0.6%	1.0%
Al Rubeaan, 2014	201	5.41	1.3200	328.000	5.58	1.2900	−0.13	[−0.31; 0.05]	2.3%	1.1%
Megia, 2014	79	6.59	1.1200	78.000	6.73	1.1200	−0.12	[−0.44; 0.19]	0.7%	1.0%
Du M, 2015	38	5.94	2.0900	38.000	4.82	1.8200	0.57	[0.11; 1.02]	0.3%	0.9%
Zhang Y, 2016	40	6.12	1.0800	240.000	5.86	1.0200	0.25	[−0.08; 0.59]	0.6%	1.0%
Savona−Ventura C, 2016	459	6.70	1.4000	603.000	6.60	1.3000	0.07	[−0.05; 0.20]	4.7%	1.1%
Yang X, 2017	19	6.32	0.3300	20.000	7.25	0.5300	−2.05	[−2.84; −1.26]	0.1%	0.7%
Zhang Y, 2017	50	5.54	1.0300	50.000	5.52	0.8500	0.02	[−0.37; 0.41]	0.5%	1.0%
Burlina S, 2016	21	6.89	1.2100	21.000	7.18	0.9900	−0.26	[−0.86; 0.35]	0.2%	0.8%
Hussain Z, 2018	60	6.54	3.1600	60.000	3.91	1.3700	1.07	[0.69; 1.46]	0.5%	1.0%
Yuan X, 2018	86	5.68	0.9900	273.000	5.88	0.9500	−0.21	[−0.45; 0.03]	1.2%	1.1%
Zhang Y, 2018	50	7.40	0.9000	47.000	6.54	1.3000	0.77	[0.35; 1.18]	0.4%	0.9%
Bao W, 2018	107	5.62	1.9700	214.000	6.17	2.7800	−0.22	[−0.45; 0.02]	1.3%	1.1%
Bugatto F, 2018	22	6.46	1.1600	23.000	6.65	1.1100	−0.16	[−0.75; 0.42]	0.2%	0.8%
Al−Daghri NM, 2019	39	6.50	1.3000	63.000	6.70	1.2000	−0.16	[−0.56; 0.24]	0.4%	1.0%
Ma Y, 2019	37	6.48	1.0700	97.000	6.32	1.0800	0.15	[−0.23; 0.53]	0.5%	1.0%
Wu, 2019	65	5.74	1.0900	65.000	5.80	1.1700	−0.05	[−0.40; 0.29]	0.6%	1.0%
Kang, 2019	72	6.70	1.3200	100.000	7.02	1.3300	−0.24	[−0.54; 0.06]	0.8%	1.0%
Wang, 2019	300	6.04	1.0700	1283.000	5.49	1.0700	0.51	[0.39; 0.64]	4.3%	1.1%
Aydemir B, 2019	99	6.29	0.9700	98.000	5.93	1.1500	0.34	[0.06; 0.62]	0.9%	1.0%
Fan Y, 2020	65	6.93	0.7200	55.000	5.12	0.4400	2.96	[2.43; 3.48]	0.3%	0.9%
Mohammed Ali D, 2020	60	5.04	0.5300	30.000	5.46	0.4400	−0.83	[−1.28; −0.37]	0.3%	0.9%
Contreras−Duarte S, 2020	69	6.00	1.2700	41.000	6.82	1.2300	−0.65	[−1.04; −0.25]	0.4%	1.0%
Contreras−Duarte S, 2020	48	6.35	1.2000	41.000	6.82	1.2300	−0.38	[−0.80; 0.04]	0.4%	0.9%
Liu M, 2020	50	6.29	1.1300	47.000	6.60	1.1600	−0.27	[−0.67; 0.13]	0.4%	1.0%
Li G, 2021	23	5.78	0.7900	29.000	4.96	0.5600	1.20	[0.61; 1.80]	0.2%	0.8%
Hussain Z, 2021	60	7.20	2.3200	60.000	4.43	1.7800	1.33	[0.93; 1.73]	0.4%	1.0%
Wu L, 2021	213	5.98	1.2100	191.000	5.85	1.3300	0.10	[−0.09; 0.30]	1.8%	1.1%
Zhou J, 2021	50	6.10	1.4200	50.000	5.84	1.3000	0.19	[−0.20; 0.58]	0.5%	1.0%
Wang F, 2021	53	6.50	1.0200	46.000	6.19	2.6500	0.16	[−0.24; 0.55]	0.4%	1.0%
Balachandiran M, 2021	40	5.15	1.3900	40.000	4.59	1.6100	0.37	[−0.07; 0.81]	0.4%	0.9%
Abdualhay R, 2022	44	5.76	0.1500	45.000	4.99	0.1200	5.63	[4.68; 6.57]	0.1%	0.6%
Franzago M, 2022	33	6.64	0.5900	27.000	6.43	1.3100	0.21	[−0.30; 0.72]	0.3%	0.9%
Dualib P, 2022	56	5.32	1.6800	59.000	4.78	0.8700	0.40	[0.03; 0.77]	0.5%	1.0%
Yang J, 2022	21	5.78	0.6300	60.000	6.40	1.3000	−0.53	[−1.03; −0.02]	0.3%	0.9%
Parveen S, 2022	37	8.10	0.7200	163.000	7.28	1.2300	0.71	[0.34; 1.07]	0.5%	1.0%
Mahmood K, 2022	50	3.88	0.4200	50.000	3.96	0.4500	−0.18	[−0.58; 0.21]	0.5%	1.0%
Sobczynska−Malefora A, 2021	24	5.90	0.9600	35.000	5.90	1.5600	0.00	[−0.52; 0.52]	0.3%	0.9%
Han L, 2022	100	4.79	0.5200	100.000	3.59	0.4300	2.51	[2.13; 2.88]	0.5%	1.0%
Yuan J, 2022	23	5.66	0.2600	19.000	5.12	0.2500	2.07	[1.31; 2.84]	0.1%	0.7%
Algaba−Chueca F, 2022	62	6.11	1.1400	74.000	6.37	1.0400	−0.24	[−0.58; 0.10]	0.6%	1.0%
Bernea E, 2022	10	6.42	1.0200	8.000	6.57	1.1400	−0.13	[−1.06; 0.80]	0.1%	0.6%
Zeljkovic A, 2022	15	6.93	1.3300	48.000	7.45	1.6300	−0.33	[−0.91; 0.25]	0.2%	0.8%
Zheng W, 2022	396	6.29	1.1200	2789.000	6.52	1.1000	−0.21	[−0.31; −0.10]	6.3%	1.1%
Wang J, 2023	47	6.38	1.1300	47.000	6.71	1.1400	−0.29	[−0.69; 0.12]	0.4%	0.9%
Visiedo, 2023	20	6.47	1.3300	17.000	6.66	1.1800	−0.15	[−0.79; 0.50]	0.2%	0.8%
Jiang, 2023	719	5.97	1.0700	1205.000	6.07	1.0800	−0.09	[−0.19; 0.00]	8.2%	1.1%
Shi, 2023	1632	6.51	1.2200	9067.000	6.03	1.1200	0.42	[0.37; 0.48]	24.8%	1.1%
Linares−Pineda, 2023	16	6.59	1.4000	16.000	7.08	1.2700	−0.36	[−1.06; 0.34]	0.1%	0.7%
Nahdh Saleem, 2024	30	5.19	0.9300	22.000	5.03	1.5200	0.13	[−0.42; 0.68]	0.2%	0.8%
Nahdh Saleem, 2024	24	5.41	1.6900	22.000	5.03	1.5200	0.23	[−0.35; 0.81]	0.2%	0.8%
Heiskanen N, 2010	51	6.20	1.1000	28.000	6.70	1.2000	−0.44	[−0.90; 0.03]	0.3%	0.9%
Hsu HW, 1997	8	6.20	1.1000	8.000	5.80	1.2000	0.33	[−0.66; 1.32]	0.1%	0.5%
Huo Y, 2015	30	4.67	0.8900	27.000	4.93	0.7900	−0.30	[−0.83; 0.22]	0.3%	0.9%
Keskin FE, 2015	44	5.83	1.2900	45.000	6.26	1.1900	−0.34	[−0.76; 0.08]	0.4%	0.9%
Liao Y, 2018	16	7.35	1.4700	16.000	5.78	1.4000	1.07	[0.32; 1.81]	0.1%	0.7%
Maitland RA, 2014	29	5.31	1.1800	77.000	5.42	1.2100	−0.09	[−0.52; 0.34]	0.4%	0.9%
Maple−Brown, 2012	198	5.80	1.2000	477.000	5.90	1.1000	−0.09	[−0.25; 0.08]	2.5%	1.1%
Marseille−Tremblay C, 2018	7	6.87	1.5900	7.000	6.62	1.1900	0.17	[−0.88; 1.22]	0.1%	0.5%
Metzger BE, 1980	6	5.60	1.4700	8.000	5.50	1.1300	0.07	[−0.99; 1.13]	0.1%	0.5%
Miettinen HE, 2014	22	6.27	1.1700	30.000	6.29	1.0400	−0.02	[−0.57; 0.53]	0.2%	0.8%
Miettine HE, 2018	15	5.80	0.7000	13.000	6.00	0.7900	−0.26	[−1.01; 0.48]	0.1%	0.7%
Morimitsu LK, 2007	23	5.89	1.1300	11.000	5.06	1.0000	0.74	[0.00; 1.49]	0.1%	0.7%
Mrizak I, 2013	28	5.43	1.5400	24.000	5.12	1.2600	0.22	[−0.33; 0.76]	0.2%	0.8%
Mrizak I, 2014	30	5.19	0.3200	30.000	5.74	0.2100	−2.01	[−2.63; −1.38]	0.2%	0.8%
Sreckovic I, 2014	9	6.73	0.9000	11.000	6.40	1.1000	0.31	[−0.58; 1.20]	0.1%	0.6%
Visiedo F, 2013	8	6.03	1.2300	14.000	6.54	1.6600	−0.32	[−1.20; 0.55]	0.1%	0.6%
Weng Q, 2019	113	5.68	1.0800	396.000	5.93	1.2500	−0.21	[−0.41; 0.00]	1.6%	1.1%
Zakovicova E, 2014	35	7.00	1.0000	31.000	7.10	0.8000	−0.11	[−0.59; 0.38]	0.3%	0.9%
Anderlová K, 2022	36	6.79	1.3800	22.000	7.85	1.7800	−0.68	[−1.22; −0.13]	0.2%	0.8%
Barrett HL, 2021	22	6.80	1.3500	7.000	8.20	2.6700	−0.79	[−1.66; 0.09]	0.1%	0.6%
Bronisz A, 2007	121	5.52	1.2500	36.000	6.06	1.3000	−0.43	[−0.80; −0.05]	0.5%	1.0%
Franzago M, 2021	33	6.20	1.0400	27.000	6.60	1.3000	−0.34	[−0.85; 0.17]	0.3%	0.9%
Genova M, 2024	54	6.10	1.4100	54.000	5.70	1.0400	0.32	[−0.06; 0.70]	0.5%	1.0%
Guo Y, 2020	87	4.80	0.9000	88.000	4.50	0.7000	0.37	[0.07; 0.67]	0.8%	1.0%
Ozyildirim S, 2023	87	5.00	0.6300	36.000	4.61	0.7700	0.58	[0.18; 0.97]	0.4%	1.0%
Prakash G, 2017	139	5.02	0.8200	139.000	4.66	0.9700	0.40	[0.16; 0.64]	1.2%	1.1%
Radojicic O, 2024	53	6.85	1.2600	49.000	6.98	1.1200	−0.11	[−0.50; 0.28]	0.5%	1.0%
Tang F, 2023	660	5.98	1.2400	1.097	6.04	1.0900	−0.05	[−1.92; 1.82]	0.0%	0.2%
**Fixed effect model**	**9997**			**22234.097**			**0.13**	**[0.10; 0.16]**	**100.0%**	**−−**
**Random effects model**							**0.04**	**[−0.06; 0.14]**	**−−**	**100.0%**

Heterogeneity: $I^2 = 91\%$, $\tau^2 = 0.2333$, $p < 0.01$
