# Supplementary material for: Trimester-Specific Serum Lipid Profiles in Gestational Diabetes Mellitus: A Systematic Review, Meta-Analysis, and Meta-Regression
Source: Medicina (Kaunas). 2025 Jul 17;61(7):1290. doi: 10.3390/medicina61071290 (PMC12300116; doi:10.3390/medicina61071290)

Study	Experimental Total	Experimental Mean	Experimental SD	Control Total	Control Mean	Control SD	SMD	95%−CI	Weight (fixed)	Weight (random)
Paradisi G, 2010	12	2.66	0.8700	38	2.58	1.4200	0.06	[−0.59; 0.71]	0.1%	0.9%
Savvidou M, 2010	124	2.59	0.7400	248	2.29	0.7100	0.42	[0.20; 0.63]	0.8%	1.5%
dos Santos−Weiss I, 2012	288	2.50	0.8900	288	2.80	0.6700	−0.38	[−0.55; −0.22]	1.5%	1.5%
Li G, 2015	379	2.18	0.7200	2166	2.09	0.5900	0.15	[0.04; 0.26]	3.4%	1.6%
Kumru P, 2016	38	2.77	0.7700	295	2.24	0.6400	0.81	[0.46; 1.15]	0.3%	1.3%
Yang X, 2017	19	2.86	0.1800	20	3.40	0.1700	−3.02	[−3.97; −2.08]	0.0%	0.6%
Yuan X, 2018	86	2.48	0.6000	273	2.50	0.5700	−0.03	[−0.28; 0.21]	0.7%	1.4%
Hou W, 2018	131	2.40	0.5900	138	2.30	0.5900	0.17	[−0.07; 0.41]	0.7%	1.4%
Yuan X, 2018	86	2.50	1.3000	273	2.45	0.5500	0.06	[−0.18; 0.31]	0.7%	1.4%
Bao W, 2018	107	2.39	1.6600	214	2.24	1.1700	0.11	[−0.12; 0.34]	0.7%	1.4%
Madhu SV, 2019	45	4.11	2.9300	45	2.18	1.2000	0.85	[0.42; 1.29]	0.2%	1.2%
Bawah AT, 2019	21	4.40	0.1700	291	3.79	0.1600	3.79	[3.25; 4.32]	0.1%	1.0%
Pezeshki B, 2019	25	2.11	0.3800	301	1.97	0.2900	0.47	[0.06; 0.88]	0.2%	1.2%
Correa, 2018	16	3.01	0.9100	80	2.37	0.5300	1.05	[0.49; 1.60]	0.1%	1.0%
Wang, 2019	300	2.21	0.5500	1283	2.09	0.5600	0.21	[0.09; 0.34]	2.5%	1.5%
Alyas S, 2019	58	4.29	0.3800	100	3.40	0.3000	2.67	[2.23; 3.11]	0.2%	1.1%
Zheng T, 2019	612	2.31	0.5600	4152	2.25	0.5900	0.10	[0.02; 0.19]	5.6%	1.6%
Jia H, 2019	136	3.10	1.0000	138	2.81	0.8700	0.31	[0.07; 0.55]	0.7%	1.4%
Benhalima K, 2019	189	2.50	0.6700	1113	2.40	0.5900	0.17	[0.01; 0.32]	1.7%	1.5%
Ma S, 2020	98	2.50	0.6500	98	2.36	0.6400	0.22	[−0.06; 0.50]	0.5%	1.4%
Mohammed Ali D, 2020	60	2.59	0.2700	30	2.56	0.1400	0.13	[−0.31; 0.57]	0.2%	1.1%
Sun T, 2020	258	2.81	1.0700	1154	3.11	0.8800	−0.33	[−0.46; −0.19]	2.2%	1.5%
Contreras−Duarte S, 2020	69	2.42	0.7600	41	2.67	0.7900	−0.32	[−0.71; 0.07]	0.3%	1.2%
Zhang X, 2020	274	2.16	0.6100	1111	2.01	0.5300	0.27	[0.14; 0.41]	2.3%	1.5%
McMichael L, 2021	34	2.69	1.0900	34	2.46	0.7000	0.25	[−0.23; 0.73]	0.2%	1.1%
Tian M, 2021	51	2.60	0.6000	51	2.30	0.6200	0.49	[0.09; 0.88]	0.3%	1.2%
Wang X, 2021	607	2.09	0.5800	833	1.97	0.5200	0.22	[0.11; 0.32]	3.6%	1.6%
Wang Y, 2021	336	1.80	0.5000	672	1.60	0.4000	0.46	[0.33; 0.59]	2.3%	1.5%
Catov J, 2021	1102	2.30	0.7000	3285	2.30	0.7000	0.00	[−0.07; 0.07]	8.6%	1.6%
Coussa R, 2021	34	3.20	0.9000	124	2.70	0.8000	0.61	[0.22; 0.99]	0.3%	1.2%
Kotzaeridi G, 2021	239	2.60	0.7000	893	2.30	0.6000	0.48	[0.34; 0.63]	1.9%	1.5%
Abdualhay R, 2022	44	3.43	0.1200	45	2.82	0.0900	5.71	[4.76; 6.66]	0.0%	0.6%
An R, 2022	94	2.59	0.6500	572	2.45	0.6700	0.21	[−0.01; 0.43]	0.8%	1.5%
Chen X, 2022	6	2.01	0.5300	27	2.66	0.7500	−0.88	[−1.80; 0.03]	0.0%	0.6%
Juchnicka I, 2022	24	2.09	0.2500	24	2.01	0.2400	0.32	[−0.25; 0.89]	0.1%	1.0%
Song S, 2022	249	2.15	0.7000	879	1.99	0.5500	0.27	[0.13; 0.41]	2.0%	1.5%
Shen L, 2023	233	2.88	0.6800	1001	2.75	0.5500	0.23	[0.08; 0.37]	2.0%	1.5%
Zheng Y, 2022	142	2.85	0.6300	442	2.61	0.5300	0.43	[0.24; 0.62]	1.1%	1.5%
Sahoo D, 2022	20	2.48	0.3100	45	2.48	0.2200	0.00	[−0.53; 0.53]	0.1%	1.0%
Tunc S, 2022	12	2.05	0.6100	88	2.03	0.5900	0.03	[−0.57; 0.64]	0.1%	0.9%
Song S, 2022	145	1.97	0.6400	555	1.88	0.5600	0.16	[−0.03; 0.34]	1.2%	1.5%
Aslan Çin N, 2022	46	3.35	2.1500	768	2.97	2.7000	0.14	[−0.16; 0.44]	0.5%	1.4%
Zeljkovic A, 2022	15	2.88	0.8800	48	2.87	0.8300	0.01	[−0.57; 0.59]	0.1%	0.9%
Zheng W, 2022	396	2.45	0.6400	2789	2.24	0.6100	0.34	[0.24; 0.45]	3.6%	1.6%
Wang F, 2023	59	2.22	0.6000	243	2.12	0.5500	0.18	[−0.11; 0.46]	0.5%	1.4%
Cui, 2023	750	2.45	0.6600	4122	2.30	0.5700	0.26	[0.18; 0.33]	6.6%	1.6%
Liu, 2023	67	2.12	0.6100	446	1.96	0.5300	0.30	[0.04; 0.55]	0.6%	1.4%
Li, 2023	100	2.03	0.8600	218	2.14	0.6800	−0.15	[−0.39; 0.09]	0.7%	1.4%
Duo, 2022	300	2.22	0.7100	1043	1.98	0.5500	0.41	[0.28; 0.54]	2.4%	1.5%
Zou, 2023	40	3.74	0.7100	65	3.55	1.2900	0.17	[−0.22; 0.56]	0.3%	1.2%
Wang, 2023	162	1.42	0.3300	48	1.38	0.3600	0.12	[−0.20; 0.44]	0.4%	1.3%
Gao, 2023	37	2.18	0.7300	553	2.17	0.5500	0.02	[−0.32; 0.35]	0.4%	1.3%
Mustaniemi, 2023	1040	2.54	0.6800	958	2.29	0.6800	0.37	[0.28; 0.46]	5.1%	1.6%
Cui, 2023	150	3.91	0.7600	150	2.65	0.2100	2.25	[1.96; 2.54]	0.5%	1.4%
Duo, 2023	272	2.20	0.5900	1017	1.90	0.5200	0.56	[0.42; 0.70]	2.2%	1.5%
Ma, 2024	201	2.08	0.7100	872	1.88	0.6600	0.30	[0.14; 0.45]	1.7%	1.5%
Zhao, 2024	261	2.16	0.6100	1327	2.01	0.5300	0.28	[0.14; 0.41]	2.3%	1.5%
Rajeevan, 2024	29	3.84	0.8900	143	3.88	1.1700	−0.04	[−0.43; 0.36]	0.3%	1.2%
Niu, 2024	519	1.92	0.6900	1281	1.88	0.6300	0.06	[−0.04; 0.16]	3.9%	1.6%
Ma, 2024	103	2.03	0.8600	225	2.15	0.6800	−0.16	[−0.40; 0.07]	0.7%	1.4%
Hou W, 2016	268	2.50	0.7000	474	2.50	0.7000	0.00	[−0.15; 0.15]	1.8%	1.5%
Houde AA, 2013	26	2.62	0.7200	74	2.53	0.7400	0.12	[−0.33; 0.57]	0.2%	1.1%
Pazhohan A, 2017	176	2.90	0.7300	778	2.75	0.6800	0.22	[0.05; 0.38]	1.5%	1.5%
Ren Z, 2020	51	3.98	1.3700	48	4.23	1.4200	−0.18	[−0.57; 0.22]	0.3%	1.2%
Ruchat SM, 2013	30	2.48	0.7000	14	2.79	0.8900	−0.40	[−1.04; 0.24]	0.1%	0.9%
Bawah AT, 2019	70	4.69	1.4400	70	3.27	1.5700	0.94	[0.59; 1.29]	0.3%	1.3%
Li J, 2021	80	2.52	0.6700	317	2.36	0.6600	0.24	[0.00; 0.49]	0.7%	1.4%
Lu L, 2022	74	4.65	0.5400	414	3.64	0.4700	2.10	[1.82; 2.38]	0.5%	1.4%
Song S, 2021	239	2.12	0.4300	843	2.00	0.3900	0.30	[0.16; 0.44]	1.9%	1.5%
Sun J, 2021	144	2.12	0.5500	600	2.04	0.5300	0.15	[−0.03; 0.33]	1.2%	1.5%
Wang N, 2021	532	2.35	0.5500	516	2.22	0.5600	0.23	[0.11; 0.36]	2.7%	1.5%
Wang X, 2022	49	2.34	0.6000	50	2.07	0.4900	0.49	[0.09; 0.89]	0.3%	1.2%
Wang W, 2023	256	2.74	0.9200	2272	2.61	0.8100	0.16	[0.03; 0.29]	2.4%	1.5%
Wani K, 2020	123	3.10	1.3000	375	3.76	1.2000	−0.54	[−0.74; −0.33]	0.9%	1.5%
Zhao X, 2023	231	2.12	0.5400	1091	2.03	0.5500	0.16	[0.02; 0.31]	2.0%	1.5%
**Fixed effect model**	**13699**			**47712**			**0.22**	**[0.20; 0.24]**	**100.0%**	**−−**
**Random effects model**							**0.32**	**[0.24; 0.41]**	**−−**	**100.0%**

Heterogeneity: $I^2 = 94\%$, $\tau^2 = 0.1271$, $p < 0.01$

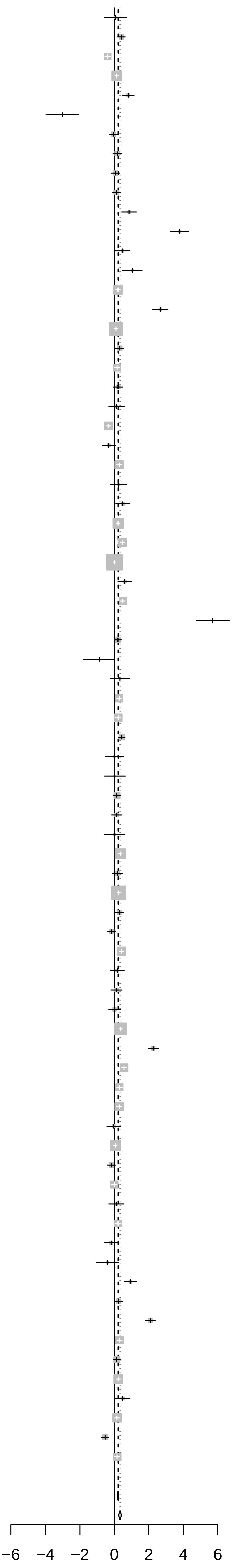

Supplement: Supplementary file 1 [file medicina-61-01290-s001.zip › Figure S25 LDL 1st trimester.pdf]
