# Supplementary material for: Trimester-Specific Serum Lipid Profiles in Gestational Diabetes Mellitus: A Systematic Review, Meta-Analysis, and Meta-Regression
Source: Medicina (Kaunas). 2025 Jul 17;61(7):1290. doi: 10.3390/medicina61071290 (PMC12300116; doi:10.3390/medicina61071290)

Study	Experimental Total	Experimental Mean	Experimental SD	Control Total	Control Mean	Control SD	SMD	95%−CI	Weight (fixed)	Weight (random)
Clark C, 1997	52	3.03	1.0700	127	3.31	0.9200	−0.29	[−0.61; 0.04]	0.3%	0.5%
Toescu V, 2004	12	3.10	0.5500	17	2.10	0.5000	1.87	[0.96; 2.77]	0.0%	0.2%
Tarim E, 2004	28	3.25	1.0100	210	3.12	0.8200	0.15	[−0.24; 0.55]	0.2%	0.4%
Di Cianni G, 2005	36	3.90	0.8000	121	4.01	1.0000	−0.11	[−0.49; 0.26]	0.2%	0.4%
Tarim E, 2006	30	3.33	0.8000	40	3.31	1.1800	0.02	[−0.45; 0.49]	0.1%	0.4%
Qui C, 2007	105	2.25	0.8200	96	2.56	0.7800	−0.39	[−0.66; −0.11]	0.4%	0.5%
Altinova A, 2007	34	3.31	0.7000	31	3.12	0.7200	0.26	[−0.22; 0.75]	0.1%	0.4%
Molnar J, 2008	17	3.80	1.3200	20	3.90	0.9400	−0.09	[−0.73; 0.56]	0.1%	0.3%
Davari−Tanha F, 2008	40	2.68	0.5400	40	2.53	0.7200	0.23	[−0.21; 0.67]	0.2%	0.4%
Idzior−Walus B, 2008	44	3.30	1.0000	17	3.50	0.8000	−0.21	[−0.77; 0.35]	0.1%	0.4%
Rizzo M, 2008	27	3.30	0.6000	23	3.20	0.8000	0.14	[−0.42; 0.70]	0.1%	0.4%
McGrowder D, 2009	84	3.32	2.2000	90	3.05	3.2300	0.10	[−0.20; 0.39]	0.3%	0.5%
Kuzmicki M, 2009	81	4.00	1.2600	82	3.40	1.6300	0.41	[0.10; 0.72]	0.3%	0.5%
Habib F, 2009	100	3.26	1.1300	100	2.93	1.1300	0.29	[0.01; 0.57]	0.4%	0.5%
Su Y, 2010	63	3.00	0.9000	58	3.10	0.9000	−0.11	[−0.47; 0.25]	0.2%	0.5%
Stein S, 2010	40	3.80	1.9000	80	3.70	1.6000	0.06	[−0.32; 0.44]	0.2%	0.4%
Coskun A, 2010	21	2.71	1.0000	24	2.35	0.4900	0.46	[−0.13; 1.05]	0.1%	0.4%
Santos I, 2010	150	3.20	1.1000	600	3.00	1.1000	0.18	[0.00; 0.36]	0.9%	0.5%
Paradisi G, 2010	12	3.65	1.0700	38	3.49	1.7900	0.10	[−0.55; 0.74]	0.1%	0.3%
Caglar G, 2011	19	3.59	1.0800	15	3.51	0.8000	0.08	[−0.60; 0.76]	0.1%	0.3%
Ozuguz U, 2011	61	3.67	1.0800	40	3.49	0.9300	0.17	[−0.22; 0.57]	0.2%	0.4%
Winhofer Y, 2010	26	3.98	1.0000	52	3.98	0.9000	0.00	[−0.47; 0.47]	0.1%	0.4%
Ping F, 2012	488	3.36	0.8500	582	3.49	0.8600	−0.15	[−0.27; −0.03]	2.0%	0.5%
Vural M, 2012	39	2.94	1.0000	40	2.79	0.7200	0.17	[−0.27; 0.61]	0.1%	0.4%
Baykus Y, 2012	20	4.18	0.8200	20	4.06	1.0000	0.13	[−0.49; 0.75]	0.1%	0.3%
Alanbay I, 2012	37	3.49	0.9500	42	3.79	0.7500	−0.35	[−0.80; 0.10]	0.1%	0.4%
Rezvan N, 2011	35	2.97	0.8900	35	2.92	0.7100	0.06	[−0.41; 0.53]	0.1%	0.4%
Khan R,2012	103	2.40	0.4900	97	2.27	0.4100	0.29	[0.01; 0.56]	0.4%	0.5%
Gkiomisi A, 2013	44	3.42	1.1900	44	4.02	1.1900	−0.50	[−0.92; −0.08]	0.2%	0.4%
Atay A, 2013	65	3.67	0.7200	66	2.01	0.6200	2.46	[2.00; 2.91]	0.1%	0.4%
dos Santos−Weiss I, 2012	288	2.60	0.9600	288	3.70	1.0400	−1.10	[−1.27; −0.92]	0.9%	0.5%
Todoric J, 2013	64	3.46	1.0400	165	3.82	0.9000	−0.38	[−0.67; −0.09]	0.3%	0.5%
Wang D, 2013	30	3.57	0.8000	60	3.42	0.7000	0.20	[−0.24; 0.64]	0.2%	0.4%
Kuzmicki M, 2014	130	3.40	1.2600	140	2.40	0.9600	0.89	[0.64; 1.15]	0.5%	0.5%
Atay A, 2014	68	2.30	0.2300	73	2.09	0.1500	1.08	[0.73; 1.44]	0.2%	0.5%
Javadian P, 2013	52	3.33	0.3300	50	2.04	0.5900	2.69	[2.15; 3.23]	0.1%	0.4%
Ebert T, 2014	74	4.05	1.9100	74	3.73	1.5700	0.18	[−0.14; 0.50]	0.3%	0.5%
Bullon, 2013	26	3.40	1.2200	162	3.19	0.7600	0.25	[−0.16; 0.66]	0.2%	0.4%
Guimarães, 2014	150	3.25	1.1000	295	3.20	1.1000	0.05	[−0.15; 0.24]	0.8%	0.5%
Houde, 2014	27	3.24	0.7600	99	3.62	1.0000	−0.40	[−0.82; 0.03]	0.2%	0.4%
Atay, 2013	37	3.60	0.7200	38	2.02	0.6200	2.33	[1.74; 2.92]	0.1%	0.4%
Wei, 2014	76	3.40	0.5500	86	2.58	0.4500	1.63	[1.28; 1.99]	0.2%	0.5%
Wei, 2014	37	3.40	0.7000	26	4.00	0.9000	−0.75	[−1.27; −0.23]	0.1%	0.4%
Reyes Lopez	90	2.68	0.9000	108	2.68	0.9300	0.00	[−0.28; 0.28]	0.4%	0.5%
Hesham, 2015	112	3.78	1.3800	218	2.60	1.6200	0.76	[0.53; 1.00]	0.5%	0.5%
Beigi, 2015	40	2.97	0.6500	40	3.00	0.5000	−0.05	[−0.49; 0.39]	0.2%	0.4%
Trebotic, 2015	21	4.06	1.3000	19	3.20	1.1000	0.70	[0.06; 1.34]	0.1%	0.3%
Telejko, 2015	49	3.10	1.5600	30	3.80	1.2600	−0.48	[−0.94; −0.02]	0.1%	0.4%
Lehmann, 2015	9	3.00	0.9900	15	3.30	0.8900	−0.31	[−1.14; 0.52]	0.0%	0.3%
Simon Muela, 2015	66	3.70	0.9700	71	3.96	0.9700	−0.27	[−0.60; 0.07]	0.3%	0.5%
Altinova A, 2015	30	3.47	1.0700	35	3.52	1.2600	−0.04	[−0.53; 0.45]	0.1%	0.4%
De Melo SF, 2015	200	3.20	1.0600	200	3.15	1.0500	0.05	[−0.15; 0.24]	0.8%	0.5%
Takhshid MA, 2015	70	2.95	0.7500	70	3.07	0.7200	−0.16	[−0.49; 0.17]	0.3%	0.5%
Takhshid MA, 2015	65	2.87	0.7500	70	3.10	0.7000	−0.32	[−0.66; 0.02]	0.3%	0.5%
Wurst U, 2015	74	4.05	1.9100	74	3.73	1.5700	0.18	[−0.14; 0.50]	0.3%	0.5%
Li XM, 2015	16	3.58	0.7600	15	3.40	0.6600	0.25	[−0.46; 0.95]	0.1%	0.3%
Li XM, 2015	16	2.86	0.7200	15	3.59	0.8100	−0.93	[−1.68; −0.18]	0.1%	0.3%
Li XM, 2015	16	3.14	0.6400	15	3.61	0.6900	−0.69	[−1.42; 0.04]	0.1%	0.3%
Korkmazer E, 2015	39	3.18	1.0400	40	3.60	1.3000	−0.35	[−0.80; 0.09]	0.1%	0.4%
Iyidir OT, 2014	26	3.80	1.3400	24	3.38	1.0400	0.34	[−0.22; 0.90]	0.1%	0.4%
Demirpençe M, 2016	20	3.23	0.9400	11	4.06	1.6000	−0.67	[−1.43; 0.09]	0.1%	0.3%
Zhang Y, 2016	40	3.39	0.9600	240	3.01	0.7400	0.49	[0.15; 0.83]	0.3%	0.5%
Edu A, 2016	13	4.18	1.0300	96	3.70	0.7200	0.63	[0.04; 1.21]	0.1%	0.4%
Ertug EY, 2016	29	3.21	1.0600	20	3.65	1.3400	−0.37	[−0.94; 0.21]	0.1%	0.4%
Mou Y, 2016	40	2.47	0.5300	40	2.80	0.5600	−0.60	[−1.05; −0.15]	0.1%	0.4%
Zheng D, 2016	50	2.23	0.4700	50	2.44	0.6100	−0.38	[−0.78; 0.01]	0.2%	0.4%
Khosrowbeygi A, 2015	30	3.68	1.8100	30	3.86	1.3700	−0.11	[−0.62; 0.40]	0.1%	0.4%
Aydemir B, 2015	116	2.93	0.3400	120	2.91	0.2500	0.07	[−0.19; 0.32]	0.4%	0.5%
Qiu YH, 2016	223	2.93	0.6400	265	3.18	0.6800	−0.38	[−0.56; −0.20]	0.9%	0.5%
Ye D, 2016	556	3.30	0.8000	500	3.10	0.7000	0.26	[0.14; 0.39]	2.0%	0.5%
Mac−Marcjanek K, 2017	104	3.53	0.8200	41	3.50	0.6100	0.04	[−0.32; 0.40]	0.2%	0.4%
Zhou X, 2017	180	3.61	0.8400	60	3.07	0.5200	0.70	[0.40; 1.00]	0.3%	0.5%
del Mar Roca−Rodríguez M, 2017	63	7.80	1.9000	63	8.20	2.3400	−0.19	[−0.54; 0.16]	0.2%	0.5%
Usluogullari B, 2017	48	2.82	0.7500	46	2.87	0.8500	−0.06	[−0.47; 0.34]	0.2%	0.4%
Mousavi SN, 2017	200	2.69	0.8900	200	3.23	0.9000	−0.60	[−0.80; −0.40]	0.7%	0.5%
Ersoy GS, 2016	62	3.93	1.0200	73	3.70	1.0600	0.22	[−0.12; 0.56]	0.3%	0.5%
Jameshorani M, 2018	65	2.67	0.8900	65	3.21	0.9100	−0.60	[−0.95; −0.24]	0.2%	0.5%
Yang Y, 2016	209	3.23	0.5900	215	2.82	0.8400	0.56	[0.37; 0.76]	0.8%	0.5%
Barat S, 2018	250	3.18	0.8200	87	3.17	1.1200	0.01	[−0.23; 0.25]	0.5%	0.5%
Yuan X, 2018	86	2.60	0.6600	273	2.95	0.6500	−0.54	[−0.78; −0.29]	0.5%	0.5%
Eken MK, 2018	63	4.00	0.9000	64	3.80	1.0000	0.21	[−0.14; 0.56]	0.2%	0.5%
Bukowiecka−Matusiak M, 2018	32	3.60	1.1100	11	3.10	0.6700	0.48	[−0.21; 1.17]	0.1%	0.3%
Bagci H, 2018	40	3.21	0.8200	40	3.25	0.6900	−0.05	[−0.49; 0.39]	0.2%	0.4%
Khosrowbeygi A, 2018	40	3.62	1.6400	40	3.78	1.2700	−0.11	[−0.55; 0.33]	0.2%	0.4%
Franzago M, 2018	104	3.39	1.5800	124	2.09	1.8100	0.76	[0.49; 1.03]	0.4%	0.5%
Al−Ajlan A, 2018	116	3.10	0.9000	303	3.00	0.8000	0.12	[−0.09; 0.33]	0.6%	0.5%
Al−Daghri NM, 2018	63	3.20	0.8000	54	3.10	0.8000	0.12	[−0.24; 0.49]	0.2%	0.4%
Bao W, 2018	107	2.79	1.5500	214	2.81	1.7600	−0.01	[−0.24; 0.22]	0.5%	0.5%
Siddiqui K, 2017	14	3.08	1.1300	21	1.98	0.6200	1.25	[0.51; 2.00]	0.1%	0.3%
Cao W, 2018	33	3.42	0.2000	33	2.66	0.1000	4.75	[3.79; 5.72]	0.0%	0.2%
Yue CY, 2018	88	3.66	0.7500	456	3.70	0.8500	−0.05	[−0.28; 0.18]	0.6%	0.5%
Tuzun D, 2018	54	2.82	0.8800	33	3.02	0.8600	−0.23	[−0.66; 0.21]	0.2%	0.4%
Siddiqui K, 2019	44	3.16	0.8800	48	2.88	0.9700	0.30	[−0.11; 0.71]	0.2%	0.4%
Demi E, 2019	85	2.97	1.3000	90	2.86	0.9300	0.10	[−0.20; 0.39]	0.3%	0.5%
Li S, 2018	90	3.20	0.8400	90	3.21	0.8200	−0.01	[−0.30; 0.28]	0.3%	0.5%
de la Torre NG, 2019	130	3.17	0.9100	802	3.12	0.8300	0.06	[−0.13; 0.24]	0.8%	0.5%
Pezeshki B, 2019	30	1.97	0.5200	301	1.97	0.2900	0.00	[−0.38; 0.38]	0.2%	0.4%
Wu, 2019	40	3.23	0.6300	40	3.43	0.4800	−0.35	[−0.80; 0.09]	0.1%	0.4%
Kang, 2019	72	2.54	0.7600	100	2.39	0.6800	0.21	[−0.09; 0.51]	0.3%	0.5%
Huang, 2019	33	3.38	0.6900	293	3.20	0.7700	0.24	[−0.12; 0.60]	0.2%	0.4%
Alhabri, 2019	200	3.70	0.9000	200	3.70	1.0000	0.00	[−0.20; 0.20]	0.8%	0.5%
Anjum, 2019	25	2.89	0.4500	50	2.95	0.9200	−0.07	[−0.55; 0.41]	0.1%	0.4%
Layton, 2018	67	3.26	0.8500	739	3.41	0.8900	−0.17	[−0.42; 0.08]	0.5%	0.5%
Wang, 2019	300	2.96	0.8200	1283	2.76	0.7700	0.26	[0.13; 0.38]	1.8%	0.5%
Abo−Elmatty, 2018	85	4.86	0.9000	80	3.43	1.2200	1.33	[1.00; 1.67]	0.3%	0.5%
Abo−Elmatty, 2018	85	4.83	0.9200	80	3.43	1.2300	1.29	[0.95; 1.62]	0.3%	0.5%
Alyas S, 2019	58	5.88	0.6900	100	5.22	0.7000	0.94	[0.60; 1.28]	0.3%	0.5%
Benhalima K, 2019	189	3.40	0.8900	1113	3.50	0.9600	−0.11	[−0.26; 0.05]	1.2%	0.5%
Abd KI, 2020	45	3.11	0.8600	45	2.79	0.5900	0.43	[0.01; 0.85]	0.2%	0.4%
Bulmus F, 2020	30	3.64	0.9800	30	3.36	0.9900	0.28	[−0.23; 0.79]	0.1%	0.4%
Jack−Roberts C, 2020	33	3.31	1.6600	29	3.40	1.2400	−0.06	[−0.56; 0.44]	0.1%	0.4%
Khosrowbeygi A, 2020	49	3.58	1.5400	51	3.71	1.2900	−0.09	[−0.48; 0.30]	0.2%	0.4%
Liu Y, 2020	30	3.40	0.7700	30	3.54	0.7200	−0.19	[−0.69; 0.32]	0.1%	0.4%
Mosavat M, 2020	53	2.59	1.0200	43	3.02	0.7200	−0.47	[−0.88; −0.07]	0.2%	0.4%
Mohammed Ali D, 2020	60	3.33	0.2700	30	2.61	0.2700	2.64	[2.05; 3.23]	0.1%	0.4%
Sun M, 2020	64	4.89	0.8200	128	4.63	0.2100	0.52	[0.21; 0.82]	0.3%	0.5%
Yavuzkir S, 2020	30	3.16	0.5800	30	2.88	0.6200	0.46	[−0.05; 0.97]	0.1%	0.4%
Zhong L, 2020	40	3.20	0.6800	40	3.07	0.6700	0.19	[−0.25; 0.63]	0.2%	0.4%
Liu L, 2020	61	2.22	0.8000	122	2.37	0.7600	−0.19	[−0.50; 0.11]	0.3%	0.5%
Contreras−Duarte S, 2020	69	3.01	0.8500	41	3.23	0.8000	−0.26	[−0.65; 0.13]	0.2%	0.4%
Contreras−Duarte S, 2020	48	2.52	0.6800	41	3.23	0.8000	−0.95	[−1.40; −0.51]	0.2%	0.4%
Gülcü Bulmus F, 2020	30	3.64	0.9800	30	3.36	0.9900	0.28	[−0.23; 0.79]	0.1%	0.4%
O'Malley E, 2020	108	3.40	1.2000	94	3.10	1.3000	0.24	[−0.04; 0.52]	0.4%	0.5%
Liu T, 2020	300	2.92	0.7000	261	2.51	0.7100	0.58	[0.41; 0.75]	1.0%	0.5%
Lu W, 2021	199	3.24	0.9600	199	2.91	0.7400	0.38	[0.19; 0.58]	0.7%	0.5%
Mei J, 2021	104	3.49	0.2800	104	3.15	0.1700	1.46	[1.16; 1.77]	0.3%	0.5%
Meng X, 2021	34	2.63	0.7000	34	3.08	0.6300	−0.67	[−1.16; −0.18]	0.1%	0.4%
Sudana N, 2021	25	3.85	0.3400	50	2.77	0.2900	3.48	[2.73; 4.22]	0.1%	0.3%
Raczkowska B, 2021	26	1.96	0.6900	29	3.38	1.8400	−0.99	[−1.55; −0.42]	0.1%	0.4%
Ramasammy R, 2021	210	2.87	0.9500	209	2.50	0.5900	0.47	[0.27; 0.66]	0.8%	0.5%
Ramanjaneya M, 2021a	34	2.90	0.9000	16	3.10	1.0000	−0.21	[−0.81; 0.38]	0.1%	0.4%
Ramanjaneya M, 2021b	46	3.00	0.9000	26	3.00	0.9000	0.00	[−0.48; 0.48]	0.1%	0.4%
Kapustin V, 2021	88	3.39	0.3000	38	3.27	0.2500	0.42	[0.03; 0.80]	0.2%	0.4%
Kapustin V, 2021	42	3.22	0.3600	38	3.27	0.2500	−0.16	[−0.60; 0.28]	0.2%	0.4%
Zhang L, 2021	36	2.90	0.9300	38	3.15	0.8100	−0.28	[−0.74; 0.17]	0.1%	0.4%
Rakchna M, 2021	73	3.46	0.5200	72	3.03	0.4400	0.89	[0.55; 1.23]	0.2%	0.5%
Baryla I, 2021	98	3.60	1.1700	37	3.70	0.9200	−0.09	[−0.47; 0.29]	0.2%	0.4%
Abd−Eltawab A, 2020	109	3.32	0.4100	103	2.46	0.2800	2.43	[2.07; 2.78]	0.2%	0.5%
Ren M, 2021	56	3.42	0.3900	56	2.71	0.3200	1.98	[1.52; 2.43]	0.1%	0.4%
Xu D, 2021	80	3.06	0.6900	80	2.86	0.8600	0.26	[−0.06; 0.57]	0.3%	0.5%
Benhalima K, 2021	97	3.50	0.5900	327	3.50	1.0400	0.00	[−0.23; 0.23]	0.6%	0.5%
Eppel D, 2021	27	3.04	0.8500	40	2.91	0.8000	0.16	[−0.33; 0.65]	0.1%	0.4%
Onat T, 2021	60	2.86	0.5000	52	2.82	0.5200	0.08	[−0.29; 0.45]	0.2%	0.4%
Wang Y, 2021	855	3.10	0.9000	785	2.80	0.7000	0.37	[0.27; 0.47]	3.1%	0.5%
Bulut A, 2021	22	3.69	0.8300	29	3.51	0.8700	0.21	[−0.35; 0.76]	0.1%	0.4%
Yu X, 2021	573	3.49	1.0300	740	3.35	1.0400	0.14	[0.03; 0.24]	2.4%	0.5%
Shalabi T, 2021	114	2.61	0.2000	114	2.29	0.2000	1.59	[1.30; 1.89]	0.3%	0.5%
Adiga U, 2021	100	3.85	1.2900	100	4.00	1.1600	−0.12	[−0.40; 0.16]	0.4%	0.5%
Cheng E, 2022	163	2.69	0.8300	145	2.75	1.1400	−0.06	[−0.28; 0.16]	0.6%	0.5%
Huang G, 2022	566	3.49	1.0300	710	3.40	1.0200	0.09	[−0.02; 0.20]	2.4%	0.5%
Juchnicka I, 2022	24	3.09	0.2500	24	3.09	1.0600	0.00	[−0.57; 0.57]	0.1%	0.4%
Liu L, 2021	69	2.57	0.5900	71	2.73	0.6600	−0.25	[−0.59; 0.08]	0.3%	0.5%
Obaidullah M, 2021	132	3.87	0.8400	100	3.35	0.8800	0.60	[0.34; 0.87]	0.4%	0.5%
Adiga U, 2022	74	3.82	1.1000	132	4.00	1.2900	−0.15	[−0.43; 0.14]	0.4%	0.5%
Shen L, 2023	233	3.64	0.9500	1001	3.59	0.7400	0.06	[−0.08; 0.21]	1.4%	0.5%
Sahoo D, 2022	20	3.02	0.2600	45	2.99	0.1800	0.14	[−0.38; 0.67]	0.1%	0.4%
Arslan A, 2022	40	3.14	0.8600	37	3.34	1.4100	−0.17	[−0.62; 0.28]	0.1%	0.4%
Hu W, 2022	315	2.31	0.5600	1876	2.26	0.5400	0.09	[−0.03; 0.21]	2.0%	0.5%
Wu Y, 2022	130	2.93	0.6900	170	2.90	0.8800	0.04	[−0.19; 0.27]	0.6%	0.5%
Cibickova L, 2022	43	3.20	1.3500	19	3.20	1.0800	0.00	[−0.54; 0.54]	0.1%	0.4%
Karagoz ZK, 2022	30	4.43	1.1100	30	4.30	0.5100	0.15	[−0.36; 0.66]	0.1%	0.4%
Wang, Y 2022	232	3.31	0.8200	696	3.51	0.8100	−0.25	[−0.39; −0.10]	1.3%	0.5%
Jia X, 2022	62	1.88	0.6500	58	1.87	0.5600	0.02	[−0.34; 0.37]	0.2%	0.4%
Zeljkovic A, 2022	15	3.58	1.5400	48	3.80	1.1300	−0.18	[−0.76; 0.40]	0.1%	0.4%
Wang J, 2023	47	2.33	0.6100	47	2.08	0.4800	0.45	[0.04; 0.86]	0.2%	0.4%
Ma, 2023	110	3.22	0.9900	110	3.32	1.1000	−0.10	[−0.36; 0.17]	0.4%	0.5%
Akash, 2023	400	3.27	0.6700	100	2.72	0.3900	0.88	[0.65; 1.11]	0.6%	0.5%
Hou, 2021	189	3.56	0.3500	100	3.23	0.3100	0.98	[0.72; 1.23]	0.4%	0.5%
Kagitci, 2023	30	3.60	1.2100	30	3.83	1.0600	−0.20	[−0.71; 0.31]	0.1%	0.4%
Burzynska−Pedziwiatr, 2023	32	3.49	1.1700	11	3.08	0.6500	0.38	[−0.31; 1.07]	0.1%	0.3%
Shi, 2023	1632	3.22	1.2700	9067	3.13	1.1300	0.08	[0.03; 0.13]	10.5%	0.5%
Li, 2023	148	2.53	0.6700	296	2.45	0.7500	0.11	[−0.09; 0.31]	0.7%	0.5%
Ali Khan, 2023	90	3.83	0.8600	90	3.71	0.9800	0.13	[−0.16; 0.42]	0.3%	0.5%
Shen, 2024	12	3.23	0.8300	10	3.04	1.1200	0.19	[−0.65; 1.03]	0.0%	0.3%
Thilak, 2024	60	3.80	3.2800	60	1.95	0.7900	0.77	[0.40; 1.14]	0.2%	0.4%
Özer, 2024	44	3.01	0.9200	44	3.32	0.8100	−0.35	[−0.78; 0.07]	0.2%	0.4%
Ghaneialvar, 2024	40	3.40	0.8300	40	3.27	0.5200	0.19	[−0.25; 0.63]	0.2%	0.4%
Aziz, 2024	50	2.35	0.0800	50	1.99	0.0800	4.47	[3.72; 5.21]	0.1%	0.3%
Uysal, 2024	52	3.69	1.0700	31	3.70	0.9400	−0.01	[−0.45; 0.44]	0.1%	0.4%
Wu, 2024	35	3.02	0.8700	70	3.00	0.6300	0.03	[−0.38; 0.43]	0.2%	0.4%
Li, 2024	1323	2.13	0.4000	1421	2.13	0.4000	0.00	[−0.07; 0.07]	5.2%	0.5%
Ademoglu EN, 2015	47	3.40	1.1000	32	3.30	1.0000	0.09	[−0.36; 0.54]	0.1%	0.4%
Anghebem−Oliveira MI, 2017	127	3.19	0.9100	125	3.38	0.9300	−0.21	[−0.45; 0.04]	0.5%	0.5%
Boghossian NS, 2017	93	3.38	1.0700	136	3.91	0.9500	−0.53	[−0.80; −0.26]	0.4%	0.5%
Dudzik D, 2014	20	3.75	0.9100	20	3.51	0.7600	0.28	[−0.34; 0.90]	0.1%	0.3%
Dudzik D, 2017	24	3.66	0.9300	24	3.37	1.0100	0.29	[−0.28; 0.86]	0.1%	0.4%
Yorulmaz Ertug E, 2016	29	3.21	1.0600	20	3.64	1.0200	−0.41	[−0.98; 0.17]	0.1%	0.4%
Guimarães LO,2014	150	3.26	1.1000	295	3.21	1.1200	0.04	[−0.15; 0.24]	0.8%	0.5%
Inegol Gumus I, 2013	34	2.79	0.7500	50	2.23	0.8500	0.68	[0.24; 1.13]	0.1%	0.4%
Hollingsworth DR, 1982	5	2.94	0.5000	21	3.00	0.5500	−0.11	[−1.08; 0.87]	0.0%	0.2%
Knopp RH, 1980	22	3.62	1.0200	38	3.47	0.9200	0.15	[−0.37; 0.68]	0.1%	0.4%
Liang Z, 2015	38	3.20	1.1000	35	2.20	0.8000	1.02	[0.53; 1.51]	0.1%	0.4%
Liu B, 2016	276	3.26	0.8600	1270	3.30	0.8100	−0.05	[−0.18; 0.08]	1.7%	0.5%
Liu B, 2016	28	3.37	1.1300	25	3.44	0.9700	−0.07	[−0.60; 0.47]	0.1%	0.4%
Rojas I, 2002	38	3.57	1.0800	38	3.43	1.2500	0.12	[−0.33; 0.57]	0.1%	0.4%
Whyte K, 2013	22	2.86	0.7500	167	2.74	0.7800	0.15	[−0.29; 0.60]	0.1%	0.4%
Anderlová K, 2022	36	3.25	1.0200	22	4.15	1.2200	−0.81	[−1.36; −0.26]	0.1%	0.4%
Attique H, 2022	37	3.51	1.2200	47	3.13	0.9100	0.36	[−0.08; 0.79]	0.2%	0.4%
Beunen K, 2014	231	3.40	0.6000	1612	3.10	0.5000	0.58	[0.44; 0.72]	1.5%	0.5%
Chen Y, 2017	28	2.50	0.7400	56	2.97	0.9200	−0.54	[−1.00; −0.08]	0.1%	0.4%
Diboun I, 2020	32	2.80	0.7000	21	2.90	0.9000	−0.13	[−0.68; 0.43]	0.1%	0.4%
Franzago M, 2020	50	3.50	0.9000	50	3.00	0.8000	0.58	[0.18; 0.98]	0.2%	0.4%
Guan L, 2021	461	2.98	0.9900	626	3.14	1.0500	−0.16	[−0.28; −0.04]	2.0%	0.5%
Guo Y, 2020	87	2.80	0.7000	88	2.50	0.6000	0.46	[0.16; 0.76]	0.3%	0.5%
Huo Y, 2019	30	3.34	0.7700	28	3.40	0.6200	−0.08	[−0.60; 0.43]	0.1%	0.4%
Iimura Y, 2015	53	3.41	0.6000	1108	3.38	0.5000	0.06	[−0.22; 0.33]	0.4%	0.5%
Kralisch S, 2009	40	3.80	1.9000	80	3.70	1.6000	0.06	[−0.32; 0.44]	0.2%	0.4%
Li J, 2015	35	3.30	0.8000	43	2.60	0.5000	1.06	[0.59; 1.54]	0.1%	0.4%
Li J, 2017	100	3.11	0.9200	100	2.86	0.8700	0.28	[0.00; 0.56]	0.4%	0.5%
Li F, 2020	48	2.55	0.8700	202	2.16	0.8800	0.44	[0.13; 0.76]	0.3%	0.5%
Li Y, 2021	359	2.61	0.7300	273	2.69	0.7500	−0.11	[−0.27; 0.05]	1.2%	0.5%
Lu L, 2023	83	3.78	0.8500	649	2.83	0.5100	1.70	[1.45; 1.94]	0.5%	0.5%
Ma J, 2022	31	1.56	0.2900	69	1.42	0.1800	0.63	[0.20; 1.07]	0.2%	0.4%
Onat T, 2021	40	2.78	0.4700	40	2.79	0.4300	−0.02	[−0.46; 0.42]	0.2%	0.4%
Qin L, 2023	82	3.24	0.8600	89	3.39	0.7800	−0.18	[−0.48; 0.12]	0.3%	0.5%
Sun J, 2022	80	3.34	0.8900	79	3.26	0.7100	0.10	[−0.21; 0.41]	0.3%	0.5%
Takhshid M, 2015	75	2.94	0.7400	70	3.07	0.7100	−0.18	[−0.50; 0.15]	0.3%	0.5%
Theodorakopoulou M, 2021	21	3.90	1.1100	15	3.90	0.9600	0.00	[−0.66; 0.66]	0.1%	0.3%
Tönjes A, 2019	74	4.00	1.3000	74	3.95	1.2600	0.04	[−0.28; 0.36]	0.3%	0.5%
Torun G, 2023	50	3.29	0.6300	50	2.82	0.5800	0.77	[0.36; 1.18]	0.2%	0.4%
Tutar D, 2022	40	3.26	1.1500	40	3.67	0.8500	−0.40	[−0.84; 0.04]	0.1%	0.4%
Wang X, 2015	839	3.25	0.9000	900	3.35	0.8800	−0.11	[−0.21; −0.02]	3.3%	0.5%
Wang H, 2019	35	3.07	0.6500	35	3.05	0.5600	0.03	[−0.44; 0.50]	0.1%	0.4%
Wang H, 2019	35	3.16	0.7400	35	2.99	0.6500	0.24	[−0.23; 0.71]	0.1%	0.4%
Wójcik M, 2015	77	4.03	1.3000	34	3.76	1.2000	0.21	[−0.19; 0.62]	0.2%	0.4%
Xu L, 2013	120	3.39	0.8800	60	3.58	0.7000	−0.23	[−0.54; 0.08]	0.3%	0.5%
Xu M, 2015	55	3.25	1.0600	66	3.35	1.0400	−0.09	[−0.45; 0.26]	0.2%	0.4%
Yakout S, 2023	46	3.40	1.1000	77	2.90	0.7000	0.57	[0.20; 0.94]	0.2%	0.4%
Yi J, 2018	141	3.00	0.8800	230	2.91	0.7400	0.11	[−0.10; 0.32]	0.7%	0.5%
Zhang Y, 2015	186	2.27	1.3600	330	2.21	1.0900	0.05	[−0.13; 0.23]	0.9%	0.5%
Zhang H, 2022	110	2.96	0.7000	416	2.78	0.7200	0.25	[0.04; 0.46]	0.7%	0.5%
**Fixed effect model**	**23982**			**45869**			**0.12**	**[0.11; 0.14]**	**100.0%**	**−−**
**Random effects model**							**0.20**	**[0.14; 0.26]**	**−−**	**100.0%**

Heterogeneity: $I^2 = 91\%$, $\tau^2 = 0.1883$, $p = 0$

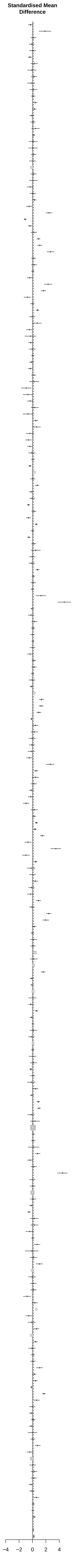

Supplement: Supplementary file 1 [file medicina-61-01290-s001.zip › Figure S26 LDL 2nd trimester.pdf]
