# Supplementary material for: Trimester-Specific Serum Lipid Profiles in Gestational Diabetes Mellitus: A Systematic Review, Meta-Analysis, and Meta-Regression
Source: Medicina (Kaunas). 2025 Jul 17;61(7):1290. doi: 10.3390/medicina61071290 (PMC12300116; doi:10.3390/medicina61071290)

Study	Experimental Total	Experimental Mean	Experimental SD	Control Total	Control Mean	Control SD	SMD	95%−CI	Weight (fixed)	Weight (random)
Koukkou E, 1997	20	3.08	1.2000	22.000	4.01	1.1000	−0.79	[−1.43; −0.16]	0.2%	0.9%
Couch S, 1998	25	2.12	0.3900	25.000	2.35	0.7200	−0.39	[−0.95; 0.17]	0.2%	0.9%
Bartha J, 2000	34	3.09	1.2000	32.000	3.33	0.9800	−0.22	[−0.70; 0.27]	0.3%	1.0%
Paradisi G, 2002	13	3.64	0.3200	15.000	3.83	0.4600	−0.46	[−1.21; 0.29]	0.1%	0.8%
Vitoratos G, 2002	15	4.13	2.0900	21.000	4.42	1.8100	−0.15	[−0.81; 0.52]	0.2%	0.9%
Toescu V, 2004	12	3.20	0.6000	17.000	2.25	0.7000	1.40	[0.56; 2.23]	0.1%	0.7%
Tsai P, 2005	34	2.70	0.6000	219.000	3.00	0.8000	−0.39	[−0.75; −0.02]	0.6%	1.1%
Szymanska M, 2008	81	3.08	0.7200	41.000	3.31	0.8300	−0.30	[−0.68; 0.08]	0.5%	1.1%
Akturk M, 2008	47	3.12	1.1000	31.000	3.38	1.2200	−0.22	[−0.68; 0.23]	0.4%	1.0%
Pfau D, 2010	40	3.80	1.9000	80.000	3.70	1.6000	0.06	[−0.32; 0.44]	0.5%	1.1%
Paradisi G, 2010	12	4.50	1.4200	38.000	3.93	1.9100	0.31	[−0.34; 0.96]	0.2%	0.9%
Akturk M, 2010	54	3.02	1.1000	69.000	3.25	1.0800	−0.21	[−0.57; 0.15]	0.6%	1.1%
Retnakaran R, 2010	136	4.72	1.1800	87.000	4.87	1.2900	−0.12	[−0.39; 0.15]	1.0%	1.1%
Culha C, 2011	24	3.45	0.7000	20.000	3.27	0.5800	0.27	[−0.32; 0.87]	0.2%	0.9%
Ghafoor S, 2012	46	3.23	1.7000	50.000	2.89	1.2700	0.23	[−0.18; 0.63]	0.5%	1.1%
Farhan S, 2012	10	2.97	0.7300	10.000	4.11	1.2000	−1.10	[−2.05; −0.14]	0.1%	0.7%
Cocelli V, 2012	62	3.67	3.0200	61.000	2.87	1.0700	0.35	[−0.01; 0.71]	0.6%	1.1%
Gkiomisi A, 2013	44	3.42	1.0600	44.000	4.16	1.4600	−0.57	[−1.00; −0.15]	0.4%	1.0%
Khan R, 2013	103	2.40	0.4900	97.000	5.04	0.6200	−4.72	[−5.27; −4.18]	0.2%	1.0%
dos Santos−Weiss I, 2012	288	3.20	1.0400	288.000	3.60	0.8900	−0.41	[−0.58; −0.25]	2.7%	1.2%
Soydinc S, 2013	42	2.94	0.8200	33.000	3.05	0.8700	−0.13	[−0.59; 0.33]	0.3%	1.0%
Kärkkäinen H, 2013	42	3.72	0.3300	32.000	4.13	0.3700	−1.17	[−1.67; −0.67]	0.3%	1.0%
Agakidou E, 2013	27	3.62	0.9600	27.000	3.60	1.3200	0.02	[−0.52; 0.55]	0.3%	1.0%
Eslamian R, 2013	112	3.33	0.7500	159.000	3.23	0.5400	0.16	[−0.09; 0.40]	1.2%	1.2%
Eslamian R, 2013	112	3.55	0.7500	159.000	3.57	0.8200	−0.03	[−0.27; 0.22]	1.2%	1.2%
Yousefzadeh G, 2013	60	3.36	1.2600	30.000	3.28	1.0800	0.07	[−0.37; 0.50]	0.4%	1.0%
Al−Hakeem M, 2014	200	3.70	0.9300	300.000	3.70	1.0000	0.00	[−0.18; 0.18]	2.3%	1.2%
Wójcik M, 2014	132	3.59	1.1800	43.000	3.67	1.5000	−0.06	[−0.41; 0.28]	0.6%	1.1%
Al Rubeaan, 2014	201	3.52	1.1600	328.000	3.57	1.0500	−0.05	[−0.22; 0.13]	2.4%	1.2%
Du M, 2015	38	3.31	0.9400	38.000	2.90	0.5100	0.54	[0.08; 0.99]	0.3%	1.0%
Zhang Y, 2016	40	3.44	0.9300	240.000	3.05	0.8200	0.47	[0.13; 0.80]	0.6%	1.1%
Savona−Ventura C, 2016	459	3.90	1.1000	603.000	4.10	1.1000	−0.18	[−0.30; −0.06]	4.9%	1.2%
Yang X, 2017	19	4.63	0.2900	20.000	4.96	0.2800	−1.13	[−1.82; −0.45]	0.2%	0.9%
Zhang Y, 2017	50	3.63	0.7300	50.000	3.53	0.8000	0.13	[−0.26; 0.52]	0.5%	1.1%
Burlina S, 2016	21	4.11	1.0000	21.000	4.15	0.9200	−0.04	[−0.65; 0.56]	0.2%	0.9%
Hussain Z, 2018	60	5.71	1.5300	60.000	3.16	1.9700	1.44	[1.03; 1.84]	0.4%	1.1%
Yuan X, 2018	86	2.49	0.8000	273.000	3.01	0.6900	−0.72	[−0.97; −0.47]	1.2%	1.2%
Zhang Y, 2018	50	3.60	0.9000	47.000	3.40	0.9000	0.22	[−0.18; 0.62]	0.5%	1.1%
Bao W, 2018	107	2.91	1.6600	214.000	3.29	2.3400	−0.18	[−0.41; 0.06]	1.3%	1.2%
Bugatto F, 2018	22	3.70	0.9800	23.000	3.78	0.9300	−0.08	[−0.67; 0.50]	0.2%	0.9%
Al−Daghri NM, 2019	39	4.00	1.1000	63.000	4.10	1.1000	−0.09	[−0.49; 0.31]	0.5%	1.1%
Wu, 2019	65	3.28	0.8700	65.000	3.35	0.8400	−0.08	[−0.43; 0.26]	0.6%	1.1%
Kang, 2019	72	3.43	0.9600	100.000	3.62	1.0500	−0.19	[−0.49; 0.12]	0.8%	1.1%
Wang, 2019	300	3.16	0.8900	1283.000	2.94	0.9200	0.24	[0.11; 0.37]	4.6%	1.2%
Aydemir B, 2019	99	3.65	0.9600	98.000	3.72	0.7800	−0.08	[−0.36; 0.20]	0.9%	1.1%
Fan Y, 2020	65	3.72	0.1300	55.000	2.39	0.1100	10.90	[9.45; 12.35]	0.0%	0.4%
Mohammed Ali D, 2020	60	3.44	0.2700	30.000	2.99	0.1900	1.81	[1.29; 2.32]	0.3%	1.0%
Contreras−Duarte S, 2020	69	3.41	0.9900	41.000	3.53	0.9600	−0.12	[−0.51; 0.27]	0.5%	1.1%
Contreras−Duarte S, 2020	48	3.12	1.0600	41.000	3.53	0.9600	−0.40	[−0.82; 0.02]	0.4%	1.0%
Liu M, 2020	50	2.87	0.8500	47.000	3.10	0.8500	−0.27	[−0.67; 0.13]	0.5%	1.1%
Li G, 2021	23	3.07	0.5400	29.000	2.69	0.3600	0.84	[0.26; 1.41]	0.2%	0.9%
Hussain Z, 2021	60	5.18	2.0100	60.000	3.10	2.2500	0.97	[0.59; 1.35]	0.5%	1.1%
Wu L, 2021	213	3.23	1.0700	191.000	3.28	1.0100	−0.05	[−0.24; 0.15]	1.9%	1.2%
Zhou J, 2021	50	3.32	0.9700	50.000	3.12	1.0800	0.19	[−0.20; 0.59]	0.5%	1.1%
Wang F, 2021	53	2.93	0.8000	46.000	3.21	1.2200	−0.27	[−0.67; 0.12]	0.5%	1.1%
Balachandiran M, 2021	40	3.46	1.1200	40.000	3.12	1.2500	0.28	[−0.16; 0.72]	0.4%	1.0%
Abdualhay R, 2022	44	3.93	1.4600	45.000	3.90	1.3500	0.02	[−0.39; 0.44]	0.4%	1.1%
Franzago M, 2022	33	3.75	1.2100	27.000	3.82	0.8200	−0.07	[−0.57; 0.44]	0.3%	1.0%
Dualib P, 2022	56	2.94	0.9500	59.000	2.48	0.7700	0.53	[0.16; 0.90]	0.5%	1.1%
Yang J, 2022	21	3.85	0.5500	60.000	4.13	1.0200	−0.30	[−0.80; 0.20]	0.3%	1.0%
Parveen S, 2022	37	3.32	0.2200	163.000	3.12	0.3200	0.65	[0.29; 1.02]	0.6%	1.1%
Mahmood K, 2022	50	1.81	0.3500	50.000	1.87	0.3800	−0.16	[−0.56; 0.23]	0.5%	1.1%
Sobczynska−Malefora A, 2021	24	3.72	0.8100	35.000	3.41	0.8200	0.37	[−0.15; 0.90]	0.3%	1.0%
Han L, 2022	100	3.89	0.4500	100.000	3.14	0.3200	1.91	[1.58; 2.25]	0.6%	1.1%
Algaba−Chueca F, 2022	62	3.21	0.9300	74.000	3.13	1.4000	0.07	[−0.27; 0.40]	0.6%	1.1%
Zeljkovic A, 2022	15	3.72	1.4300	48.000	4.27	1.3000	−0.41	[−0.99; 0.18]	0.2%	0.9%
Zheng W, 2022	396	3.32	0.9400	2789.000	3.55	0.9800	−0.24	[−0.34; −0.13]	6.6%	1.2%
Wang J, 2023	47	3.35	0.9700	47.000	3.56	0.9900	−0.21	[−0.62; 0.19]	0.4%	1.1%
Visiedo, 2023	20	3.73	1.1300	17.000	3.83	0.9400	−0.09	[−0.74; 0.55]	0.2%	0.9%
Jiang, 2023	719	2.94	0.8600	1205.000	2.37	0.4300	0.91	[0.81; 1.01]	7.8%	1.2%
Shi, 2023	1632	3.79	1.2700	9067.000	3.71	1.3800	0.06	[0.01; 0.11]	26.2%	1.2%
Nahdh Saleem, 2024	30	3.12	0.8200	22.000	3.24	1.3400	−0.11	[−0.66; 0.44]	0.2%	1.0%
Nahdh Saleem, 2024	24	3.21	1.2600	22.000	3.24	1.3400	−0.02	[−0.60; 0.56]	0.2%	0.9%
Heiskanen N, 2010	51	3.70	1.0000	28.000	4.30	1.1000	−0.57	[−1.04; −0.10]	0.3%	1.0%
Hsu HW, 1997	8	3.50	0.8000	8.000	3.20	0.7000	0.38	[−0.61; 1.37]	0.1%	0.6%
Huo Y, 2015	30	2.73	0.7500	27.000	2.89	0.6900	−0.22	[−0.74; 0.30]	0.3%	1.0%
Keskin FE, 2015	44	3.50	1.2100	45.000	3.66	1.0200	−0.14	[−0.56; 0.27]	0.4%	1.1%
Liao Y, 2018	16	4.06	0.9000	16.000	2.58	1.0700	1.46	[0.67; 2.25]	0.1%	0.8%
Maitland RA, 2014	29	2.74	1.3900	77.000	2.93	1.3400	−0.14	[−0.57; 0.29]	0.4%	1.0%
Maple−Brown, 2012	198	3.50	1.2000	477.000	3.70	1.1000	−0.18	[−0.34; −0.01]	2.6%	1.2%
Marseille−Tremblay C, 2018	7	3.67	1.1400	7.000	3.66	1.2200	0.01	[−1.04; 1.06]	0.1%	0.6%
Miettinen HE, 2014	22	3.25	1.0300	30.000	3.57	1.0400	−0.30	[−0.86; 0.25]	0.2%	0.9%
Miettine HE, 2018	15	3.10	0.8100	13.000	3.45	0.9400	−0.39	[−1.14; 0.36]	0.1%	0.8%
Morimitsu LK, 2007	23	3.37	1.0600	11.000	2.84	1.0400	0.49	[−0.24; 1.22]	0.1%	0.8%
Mrizak I, 2013	28	2.94	1.2700	24.000	2.59	1.3900	0.26	[−0.29; 0.81]	0.2%	1.0%
Mrizak I, 2014	30	2.16	0.2600	30.000	2.61	0.2600	−1.71	[−2.31; −1.11]	0.2%	0.9%
Sreckovic I, 2014	9	3.93	1.1000	11.000	3.70	1.2000	0.19	[−0.69; 1.07]	0.1%	0.7%
Visiedo F, 2013	8	3.51	0.9800	14.000	3.15	1.2600	0.30	[−0.58; 1.17]	0.1%	0.7%
Weng Q, 2019	113	2.76	0.8200	396.000	2.90	0.9200	−0.16	[−0.36; 0.05]	1.7%	1.2%
Zakovicova E, 2014	35	4.00	0.9000	31.000	4.40	0.7000	−0.49	[−0.98; 0.00]	0.3%	1.0%
Anderlová K, 2022	36	3.25	1.1400	22.000	4.12	1.5000	−0.67	[−1.21; −0.12]	0.2%	1.0%
Barrett HL, 2021	22	3.50	1.4100	7.000	4.10	2.5900	−0.33	[−1.19; 0.52]	0.1%	0.7%
Bronisz A, 2007	121	2.91	0.9800	36.000	3.48	0.9900	−0.58	[−0.96; −0.20]	0.5%	1.1%
Franzago M, 2021	33	3.70	0.7400	27.000	4.10	0.8100	−0.51	[−1.03; 0.01]	0.3%	1.0%
Genova M, 2024	54	3.58	1.1700	54.000	3.15	0.7000	0.44	[0.06; 0.82]	0.5%	1.1%
Guo Y, 2020	87	2.80	0.7000	88.000	2.50	0.6000	0.46	[0.16; 0.76]	0.8%	1.1%
Ozyildirim S, 2023	87	3.15	0.5500	36.000	2.80	0.7100	0.58	[0.18; 0.97]	0.5%	1.1%
Prakash G, 2017	139	3.00	0.5200	139.000	2.80	0.9700	0.26	[0.02; 0.49]	1.3%	1.2%
Radojicic O, 2024	53	3.85	1.0700	49.000	3.76	1.0800	0.08	[−0.31; 0.47]	0.5%	1.1%
Tang F, 2023	660	2.98	0.9700	1.097	3.17	0.9800	−0.20	[−2.07; 1.68]	0.0%	0.3%
**Fixed effect model**	**9554**			**21740.097**			**0.06**	**[0.03; 0.08]**	**100.0%**	**−−**
**Random effects model**							**0.01**	**[−0.10; 0.12]**	**−−**	**100.0%**

Heterogeneity: $I^2 = 93\%$, $\tau^2 = 0.2801$, $p < 0.01$

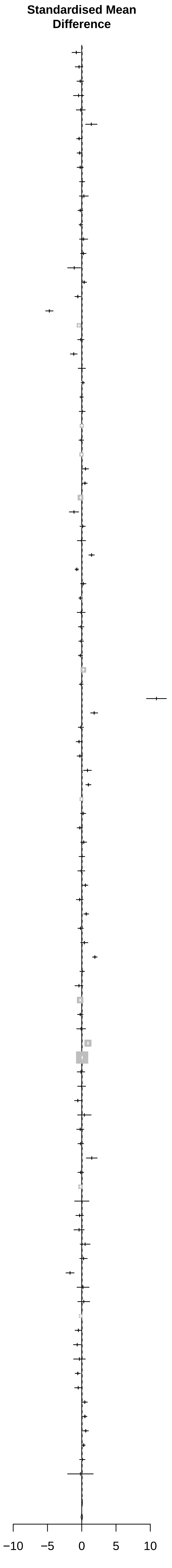

Supplement: Supplementary file 1 [file medicina-61-01290-s001.zip › Figure S27 LDL 3rd trimester.pdf]
