# Supplementary material for: Trimester-Specific Serum Lipid Profiles in Gestational Diabetes Mellitus: A Systematic Review, Meta-Analysis, and Meta-Regression
Source: Medicina (Kaunas). 2025 Jul 17;61(7):1290. doi: 10.3390/medicina61071290 (PMC12300116; doi:10.3390/medicina61071290)

Study	Experimental Total	Experimental Mean	Experimental SD	Control Total	Control Mean	Control SD	SMD	95%−CI	Weight (fixed)	Weight (random)
Paradisi G, 2010	12	1.70	0.6200	38	1.62	1.1100	0.08	[−0.57; 0.73]	0.1%	0.9%
Savvidou M, 2010	124	1.55	0.3800	248	1.68	0.3600	−0.35	[−0.57; −0.14]	0.8%	1.4%
Makgoba M, 2011	90	1.58	0.3800	158	1.74	0.3500	−0.44	[−0.70; −0.18]	0.5%	1.3%
dos Santos−Weiss I, 2012	288	1.20	0.3000	288	1.40	0.4000	−0.56	[−0.73; −0.40]	1.3%	1.4%
Li G, 2015	379	1.84	0.4600	2166	1.97	0.5000	−0.26	[−0.37; −0.15]	3.0%	1.5%
Kumru P, 2016	38	1.64	0.3400	295	1.76	0.4200	−0.29	[−0.63; 0.05]	0.3%	1.3%
Yang X, 2017	19	1.71	0.1100	20	1.99	0.0700	−2.99	[−3.93; −2.05]	0.0%	0.6%
Yuan X, 2018	86	1.57	0.3900	273	1.58	0.2700	−0.03	[−0.28; 0.21]	0.6%	1.4%
Hou W, 2018	131	1.80	0.4000	138	1.90	0.4000	−0.25	[−0.49; −0.01]	0.6%	1.4%
Yuan X, 2018	86	1.51	0.3900	273	1.55	0.3200	−0.12	[−0.36; 0.12]	0.6%	1.4%
Bao W, 2018	107	1.42	0.0600	214	1.63	0.0400	−4.40	[−4.82; −3.99]	0.2%	1.2%
Madhu SV, 2019	45	1.05	0.2200	45	1.01	0.2200	0.18	[−0.23; 0.59]	0.2%	1.2%
Bawah AT, 2019	21	1.20	0.7000	291	1.44	0.8000	−0.30	[−0.75; 0.14]	0.2%	1.1%
Pezeshki B, 2019	25	1.06	0.1700	301	1.10	0.1500	−0.26	[−0.67; 0.15]	0.2%	1.2%
Correa, 2018	16	1.55	0.3000	80	1.71	0.4500	−0.37	[−0.91; 0.17]	0.1%	1.0%
Wang, 2019	300	1.37	0.2600	1283	1.51	0.2700	−0.52	[−0.65; −0.39]	2.2%	1.5%
Alyas S, 2019	58	1.18	0.1500	100	1.55	0.2000	−2.01	[−2.40; −1.61]	0.2%	1.2%
Zheng T, 2019	612	1.87	0.5100	4152	2.04	0.7300	−0.24	[−0.33; −0.16]	4.9%	1.5%
Jia H, 2019	136	1.84	0.3800	138	1.74	0.3800	0.26	[0.02; 0.50]	0.6%	1.4%
Benhalima K, 2019	189	1.70	0.3700	1113	1.70	0.3700	0.00	[−0.15; 0.15]	1.5%	1.4%
Ma S, 2020	98	1.80	0.4100	98	1.92	0.4000	−0.30	[−0.58; −0.01]	0.4%	1.3%
Mohammed Ali D, 2020	60	1.21	0.1200	30	1.39	0.1000	−1.57	[−2.07; −1.07]	0.1%	1.1%
Sun T, 2020	258	1.69	0.4800	1154	1.59	0.3500	0.27	[0.13; 0.40]	1.9%	1.5%
Ye Y, 2020	2181	1.08	0.2200	2719	1.05	0.2100	0.14	[0.08; 0.20]	11.2%	1.5%
Contreras−Duarte S, 2020	69	1.67	0.3200	41	1.73	0.3200	−0.19	[−0.57; 0.20]	0.2%	1.2%
Zhang X, 2020	274	1.40	0.6300	1111	1.41	0.2700	−0.03	[−0.16; 0.11]	2.0%	1.5%
McMichael L, 2021	34	1.55	0.3900	34	1.71	0.4000	−0.40	[−0.88; 0.08]	0.2%	1.1%
Tian M, 2021	51	1.80	0.4000	51	1.90	0.3700	−0.26	[−0.65; 0.13]	0.2%	1.2%
Wang X, 2021	607	1.72	0.3200	833	1.71	0.3200	0.03	[−0.07; 0.14]	3.3%	1.5%
Wang Y, 2021	336	1.40	0.3000	672	1.50	0.3000	−0.33	[−0.46; −0.20]	2.1%	1.5%
Catov J, 2021	1102	1.80	0.4000	3285	1.90	0.4000	−0.25	[−0.32; −0.18]	7.6%	1.5%
Coussa R, 2021	34	1.60	0.7000	124	1.80	0.8000	−0.26	[−0.64; 0.13]	0.2%	1.2%
Hu Z, 2021	78	1.48	0.3200	30	1.55	0.2400	−0.23	[−0.65; 0.19]	0.2%	1.2%
Kotzaeridi G, 2021	239	1.45	0.2000	893	1.55	0.3000	−0.35	[−0.50; −0.21]	1.7%	1.4%
Abdualhay R, 2022	44	0.89	0.2100	45	0.93	0.1900	−0.20	[−0.61; 0.22]	0.2%	1.2%
An R, 2022	94	1.83	0.4300	572	1.98	0.4100	−0.36	[−0.58; −0.14]	0.7%	1.4%
Chen X, 2022	6	1.95	0.2300	27	1.82	0.3500	0.38	[−0.51; 1.27]	0.0%	0.7%
Juchnicka I, 2022	24	1.91	0.1900	24	2.09	0.2200	−0.86	[−1.46; −0.27]	0.1%	1.0%
Song S, 2022	249	1.43	0.3100	879	1.50	0.3200	−0.22	[−0.36; −0.08]	1.8%	1.4%
Shen L, 2023	233	1.78	0.3200	1001	1.79	0.3300	−0.03	[−0.17; 0.11]	1.8%	1.4%
Zheng Y, 2022	142	1.70	0.3000	442	1.73	0.3000	−0.10	[−0.29; 0.09]	1.0%	1.4%
Sahoo D, 2022	20	1.21	0.0500	45	1.28	0.1000	−0.79	[−1.33; −0.24]	0.1%	1.0%
Tunc S, 2022	12	1.52	0.5100	88	1.53	0.4100	−0.02	[−0.63; 0.58]	0.1%	0.9%
Song S, 2022	145	1.40	0.3100	555	1.49	0.3600	−0.26	[−0.44; −0.07]	1.1%	1.4%
Aslan Çin N, 2022	46	1.10	0.8100	768	1.06	1.0200	0.04	[−0.26; 0.34]	0.4%	1.3%
Zeljkovic A, 2022	15	1.97	0.6400	48	1.80	0.3000	0.42	[−0.17; 1.00]	0.1%	1.0%
Zheng W, 2022	396	1.45	0.2700	2789	1.53	0.2800	−0.29	[−0.39; −0.18]	3.2%	1.5%
Wang F, 2023	59	1.02	0.2100	243	0.92	0.1300	0.67	[0.38; 0.96]	0.4%	1.3%
Cui, 2023	750	1.62	0.3300	4122	1.66	0.3200	−0.12	[−0.20; −0.05]	5.9%	1.5%
Liu, 2023	67	1.47	0.2400	446	1.56	0.2800	−0.33	[−0.58; −0.07]	0.5%	1.4%
Li, 2023	100	1.88	0.5600	218	2.06	0.6200	−0.30	[−0.54; −0.06]	0.6%	1.4%
Duo, 2022	300	1.45	0.2800	1043	1.48	0.3000	−0.10	[−0.23; 0.03]	2.2%	1.5%
Zou, 2023	40	2.02	0.4300	65	2.68	1.6100	−0.51	[−0.91; −0.11]	0.2%	1.2%
Gao, 2023	37	1.53	0.4300	553	1.69	0.3400	−0.46	[−0.80; −0.13]	0.3%	1.3%
Mustaniemi, 2023	1040	0.82	0.1700	958	0.86	0.1800	−0.23	[−0.32; −0.14]	4.6%	1.5%
Cui, 2023	150	1.12	0.2300	150	3.81	0.2000	−12.45	[−13.48; −11.42]	0.0%	0.6%
Duo, 2023	272	1.43	0.3000	1017	1.50	0.3000	−0.23	[−0.37; −0.10]	2.0%	1.5%
Ma, 2024	201	1.88	0.4100	872	1.93	0.4100	−0.12	[−0.28; 0.03]	1.5%	1.4%
Zhao, 2024	261	1.40	0.6300	1327	1.41	0.2700	−0.03	[−0.16; 0.10]	2.0%	1.5%
Rajeevan, 2024	29	1.21	0.3400	143	1.21	0.3400	0.00	[−0.40; 0.40]	0.2%	1.2%
Niu, 2024	519	1.87	0.5200	1281	2.00	0.4800	−0.26	[−0.37; −0.16]	3.4%	1.5%
Ma, 2024	103	1.88	0.5500	225	2.06	0.6100	−0.30	[−0.54; −0.07]	0.6%	1.4%
Hou W, 2016	268	1.80	0.4000	474	1.80	0.4000	0.00	[−0.15; 0.15]	1.6%	1.4%
Houde AA, 2013	26	1.62	0.3400	74	1.56	0.2800	0.20	[−0.25; 0.65]	0.2%	1.1%
Pazhohan A, 2017	176	1.51	0.4700	778	1.62	0.4200	−0.26	[−0.42; −0.09]	1.3%	1.4%
Ren Z, 2020	51	1.38	0.1400	48	1.41	0.1600	−0.20	[−0.59; 0.20]	0.2%	1.2%
Ruchat SM, 2013	30	1.62	0.3200	14	1.65	0.2100	−0.10	[−0.74; 0.53]	0.1%	0.9%
Bawah AT, 2019	70	1.32	0.9600	70	1.48	0.9100	−0.17	[−0.50; 0.16]	0.3%	1.3%
Jin C, 2021	135	1.70	0.3700	135	1.80	0.3000	−0.30	[−0.54; −0.06]	0.6%	1.4%
Lu L, 2022	74	2.51	0.1300	414	2.09	0.2300	1.92	[1.65; 2.20]	0.5%	1.3%
Meyer B, 2023	43	1.59	0.4900	26	1.72	0.5600	−0.25	[−0.74; 0.24]	0.1%	1.1%
Song S, 2021	239	1.41	0.2100	843	1.49	0.2500	−0.33	[−0.47; −0.19]	1.7%	1.4%
Sun J, 2021	144	1.50	0.8100	600	1.42	0.2500	0.19	[0.01; 0.37]	1.1%	1.4%
Wang N, 2021	532	1.57	0.3100	516	1.66	0.3200	−0.29	[−0.41; −0.16]	2.4%	1.5%
Wang X, 2022	49	1.48	0.3000	50	1.48	0.2900	0.00	[−0.39; 0.39]	0.2%	1.2%
Wang W, 2023	256	1.65	0.4600	2272	1.82	0.4600	−0.37	[−0.50; −0.24]	2.1%	1.5%
Wani K, 2020	123	1.30	0.4000	375	1.40	0.3000	−0.30	[−0.51; −0.10]	0.9%	1.4%
Zhao X, 2023	231	1.42	0.2900	1091	1.41	0.2800	0.04	[−0.11; 0.18]	1.8%	1.4%
**Fixed effect model**	**15984**			**50415**			**−0.16**	**[−0.18; −0.14]**	**100.0%**	**--**
**Random effects model**							**−0.32**	**[−0.41; −0.22]**	**--**	**100.0%**

Heterogeneity: $I^2 = 96\%$, $\tau^2 = 0.1603$, $p < 0.01$

Standardised Mean Difference

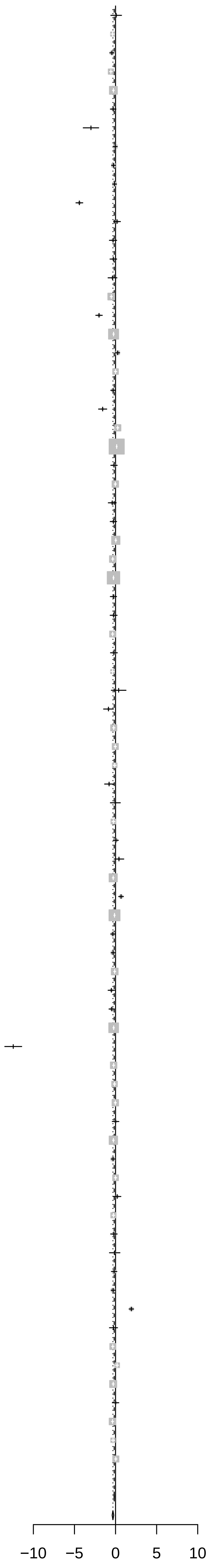

Supplement: Supplementary file 1 [file medicina-61-01290-s001.zip › Figure S28 HDL 1st trimester.pdf]
