# Supplementary material for: Trimester-Specific Serum Lipid Profiles in Gestational Diabetes Mellitus: A Systematic Review, Meta-Analysis, and Meta-Regression
Source: Medicina (Kaunas). 2025 Jul 17;61(7):1290. doi: 10.3390/medicina61071290 (PMC12300116; doi:10.3390/medicina61071290)

Study	Experimental Total	Experimental Mean	Experimental SD	Control Total	Control Mean	Control SD	SMD	95%−CI	Weight (fixed)	Weight (random)
Clark C, 1997	52	1.16	0.2700	127	1.32	0.3400	−0.50	[−0.82; −0.17]	0.3%	0.4%
Seghieri G, 2003	15	1.60	0.4000	78	1.60	0.3000	0.00	[−0.55; 0.55]	0.1%	0.4%
Tarim E, 2004	28	1.75	0.3600	210	1.81	0.3600	−0.17	[−0.56; 0.23]	0.2%	0.4%
Di Cianni G, 2005	36	1.60	0.3000	121	1.68	0.4000	−0.21	[−0.58; 0.16]	0.2%	0.4%
Tarim E, 2006	30	1.68	0.4600	40	1.62	0.6200	0.11	[−0.37; 0.58]	0.1%	0.4%
Qui C, 2007	105	1.26	0.4100	96	1.44	0.2900	−0.50	[−0.78; −0.22]	0.4%	0.5%
Altinova A, 2007	34	1.70	0.4700	31	1.81	0.3900	−0.25	[−0.74; 0.24]	0.1%	0.4%
Molnar J, 2008	17	1.80	0.4500	20	2.00	0.3400	−0.50	[−1.15; 0.16]	0.1%	0.3%
Davari−Tanha F, 2008	40	1.29	0.2000	40	1.55	0.3600	−0.88	[−1.34; −0.42]	0.1%	0.4%
Idzior−Walus B, 2008	44	1.90	0.5000	17	2.20	0.4000	−0.62	[−1.19; −0.05]	0.1%	0.4%
Rizzo M, 2008	27	1.70	0.5000	23	1.80	0.4000	−0.22	[−0.77; 0.34]	0.1%	0.4%
McGrowder D, 2009	84	1.25	0.7300	90	1.45	0.7600	−0.27	[−0.57; 0.03]	0.3%	0.5%
Kuzmicki M, 2009	81	1.50	0.1500	82	1.50	0.2200	0.00	[−0.31; 0.31]	0.3%	0.5%
Habib F, 2009	100	1.24	0.2500	100	1.42	0.2400	−0.73	[−1.02; −0.45]	0.3%	0.5%
Bo S, 2009	500	1.70	0.4000	500	1.90	0.4000	−0.50	[−0.63; −0.37]	1.8%	0.5%
Su Y, 2010	63	1.90	0.4000	58	1.90	0.3000	0.00	[−0.36; 0.36]	0.2%	0.4%
Stein S, 2010	40	1.70	0.7000	80	1.90	0.5000	−0.35	[−0.73; 0.04]	0.2%	0.4%
Coskun A, 2010	21	1.24	0.3100	24	1.26	0.2100	−0.08	[−0.66; 0.51]	0.1%	0.4%
Santos I, 2010	150	1.50	0.4000	600	1.40	0.4000	0.25	[0.07; 0.43]	0.9%	0.5%
Paradisi G, 2010	12	1.93	0.7600	38	1.93	1.3600	0.00	[−0.65; 0.65]	0.1%	0.3%
Caglar G, 2011	19	1.73	0.3600	15	1.93	0.5100	−0.45	[−1.14; 0.23]	0.1%	0.3%
Ozuguz U, 2011	61	1.65	0.3800	40	1.62	0.4100	0.08	[−0.32; 0.47]	0.2%	0.4%
Winhofer Y, 2010	26	1.83	0.3800	52	1.99	0.3600	−0.43	[−0.91; 0.04]	0.1%	0.4%
Ping F, 2012	488	2.13	0.4500	582	2.19	0.4300	−0.14	[−0.26; −0.02]	1.9%	0.5%
Vural M, 2012	39	1.29	0.2500	40	1.42	0.3600	−0.41	[−0.86; 0.03]	0.1%	0.4%
Naf S, 2012	77	1.85	0.3400	130	1.91	0.3100	−0.19	[−0.47; 0.10]	0.4%	0.5%
Baykus Y, 2012	20	1.62	0.3100	20	1.75	0.3800	−0.37	[−0.99; 0.26]	0.1%	0.3%
Alanbay I, 2012	37	1.47	0.2100	42	1.70	0.3100	−0.85	[−1.31; −0.39]	0.1%	0.4%
Rezvan N, 2011	35	1.13	0.5900	35	1.29	0.2400	−0.35	[−0.82; 0.12]	0.1%	0.4%
Khan R,2012	103	1.42	0.2100	97	1.44	0.2300	−0.09	[−0.37; 0.19]	0.4%	0.5%
Gkiomisi A, 2013	44	1.70	0.4000	44	1.88	0.4600	−0.41	[−0.84; 0.01]	0.2%	0.4%
Atay A, 2013	65	2.22	0.1300	66	1.26	0.1300	7.34	[6.38; 8.31]	0.0%	0.2%
dos Santos−Weiss I, 2012	288	1.50	0.3000	288	1.60	0.4000	−0.28	[−0.45; −0.12]	1.0%	0.5%
Barden, A 2013	53	1.50	0.3000	72	1.80	0.4000	−0.83	[−1.20; −0.46]	0.2%	0.4%
Todoric J, 2013	64	1.78	0.4000	165	1.96	0.2600	−0.59	[−0.88; −0.29]	0.3%	0.5%
Wang D, 2013	30	2.00	0.3000	60	1.91	0.3600	0.26	[−0.18; 0.70]	0.1%	0.4%
Kuzmicki M, 2014	130	1.90	0.3700	140	1.60	0.3000	0.89	[0.64; 1.14]	0.4%	0.5%
Atay A, 2014	68	1.08	0.1300	73	1.26	0.1400	−1.32	[−1.69; −0.96]	0.2%	0.4%
Javadian P, 2013	52	1.47	0.9300	50	2.37	0.4600	−1.21	[−1.63; −0.79]	0.2%	0.4%
Ebert T, 2014	74	1.82	0.8000	74	1.93	0.5100	−0.16	[−0.49; 0.16]	0.3%	0.4%
Bullon, 2013	26	1.90	0.5000	162	1.90	0.4200	0.00	[−0.41; 0.41]	0.2%	0.4%
Guimarães, 2014	150	1.47	0.3900	295	1.37	0.4200	0.24	[0.05; 0.44]	0.7%	0.5%
Houde, 2014	27	1.75	0.2900	99	1.80	0.4100	−0.13	[−0.55; 0.30]	0.2%	0.4%
Atay, 2013	37	1.11	0.1200	38	1.26	0.1300	−1.19	[−1.68; −0.69]	0.1%	0.4%
Wei, 2014	37	1.60	0.3000	26	1.90	0.3000	−0.99	[−1.52; −0.46]	0.1%	0.4%
Reyes Lopez	90	1.50	0.2000	108	1.56	0.2600	−0.25	[−0.54; 0.03]	0.4%	0.5%
Hesham, 2015	112	0.98	0.4200	218	1.08	0.4200	−0.24	[−0.47; −0.01]	0.5%	0.5%
Beigi, 2015	40	1.40	0.3300	40	1.40	0.3000	0.00	[−0.44; 0.44]	0.1%	0.4%
Trebotic, 2015	21	1.70	0.3600	19	1.80	0.4600	−0.24	[−0.86; 0.38]	0.1%	0.3%
Telejko, 2015	49	1.50	0.2200	30	1.50	0.2200	0.00	[−0.45; 0.45]	0.1%	0.4%
Lehmann, 2015	9	1.78	0.3000	15	2.12	0.3000	−1.09	[−1.99; −0.20]	0.0%	0.2%
Simon Muela, 2015	66	1.88	0.5100	71	1.91	0.3200	−0.07	[−0.41; 0.26]	0.2%	0.4%
Altinova A, 2015	30	1.28	0.3500	35	1.57	0.3400	−0.83	[−1.34; −0.32]	0.1%	0.4%
De Melo SF, 2015	200	1.44	0.3400	200	1.20	0.2800	0.77	[0.57; 0.97]	0.7%	0.5%
Takhshid MA, 2015	70	1.37	0.3600	70	1.35	0.3100	0.06	[−0.27; 0.39]	0.3%	0.4%
Takhshid MA, 2015	65	1.29	0.2800	70	1.35	0.3100	−0.20	[−0.54; 0.14]	0.2%	0.4%
Wurst U, 2015	74	1.82	0.7900	74	1.93	0.5100	−0.16	[−0.49; 0.16]	0.3%	0.4%
Li XM, 2015	16	1.98	0.3200	15	1.94	0.5800	0.08	[−0.62; 0.79]	0.1%	0.3%
Li XM, 2015	16	1.71	0.4400	15	1.99	1.0500	−0.34	[−1.05; 0.37]	0.1%	0.3%
Li XM, 2015	16	1.91	0.4000	15	1.76	0.4300	0.35	[−0.36; 1.06]	0.1%	0.3%
Korkmazer E, 2015	39	1.68	0.3600	40	1.81	0.3900	−0.34	[−0.79; 0.10]	0.1%	0.4%
Iyidir OT, 2014	26	1.56	0.3800	24	1.59	0.3800	−0.08	[−0.63; 0.48]	0.1%	0.4%
Demirpençe M, 2016	20	1.57	0.3100	11	1.59	0.5200	−0.05	[−0.79; 0.69]	0.1%	0.3%
Zhang Y, 2016	40	1.78	0.5500	240	1.86	0.4900	−0.16	[−0.49; 0.18]	0.3%	0.4%
Edu A, 2016	13	2.09	0.2500	96	1.76	0.3700	0.91	[0.32; 1.51]	0.1%	0.3%
Ertug EY, 2016	29	1.65	0.3400	20	1.78	0.4100	−0.35	[−0.92; 0.23]	0.1%	0.4%
Mou Y, 2016	40	1.10	0.2100	40	1.35	0.2300	−1.12	[−1.60; −0.65]	0.1%	0.4%
Zheng D, 2016	50	1.69	0.3800	50	1.81	0.2300	−0.38	[−0.77; 0.02]	0.2%	0.4%
Khosrowbeygi A, 2015	30	0.95	0.4400	30	1.61	0.3300	−1.68	[−2.27; −1.08]	0.1%	0.3%
Aydemir B, 2015	116	1.36	0.1600	120	1.33	0.1400	0.20	[−0.06; 0.45]	0.4%	0.5%
Qiu YH, 2016	223	1.31	0.3200	265	1.38	0.3000	−0.23	[−0.40; −0.05]	0.9%	0.5%
Ye D, 2016	556	2.10	0.6000	500	2.20	0.6000	−0.17	[−0.29; −0.05]	1.9%	0.5%
Mac−Marcjanek K, 2017	104	1.76	0.4400	41	1.95	0.4600	−0.42	[−0.79; −0.06]	0.2%	0.4%
Tu WJ, 2017	135	1.50	0.4400	1015	1.89	0.4900	−0.80	[−0.99; −0.62]	0.8%	0.5%
Zhou X, 2017	180	1.78	0.2500	60	1.82	0.2800	−0.15	[−0.45; 0.14]	0.3%	0.5%
del Mar Roca−Rodríguez M, 2017	63	3.90	0.9000	63	4.54	0.9200	−0.70	[−1.06; −0.34]	0.2%	0.4%
Mousavi SN, 2017	200	1.44	0.3000	200	1.77	0.5000	−0.80	[−1.00; −0.60]	0.7%	0.5%
Ersoy GS, 2016	62	1.64	0.3600	73	1.71	0.3000	−0.21	[−0.55; 0.13]	0.2%	0.4%
Jameshorani M, 2018	65	1.45	0.3000	65	1.77	0.5100	−0.76	[−1.12; −0.40]	0.2%	0.4%
Yang Y, 2016	209	3.23	0.5900	215	1.83	0.4200	2.74	[2.47; 3.00]	0.4%	0.5%
Barat S, 2018	250	1.38	0.3800	87	1.72	0.6700	−0.72	[−0.97; −0.47]	0.4%	0.5%
Yuan X, 2018	86	1.56	0.3600	273	1.64	0.2800	−0.27	[−0.51; −0.02]	0.5%	0.5%
Eken MK, 2018	63	1.60	0.3000	64	1.60	0.3000	0.00	[−0.35; 0.35]	0.2%	0.4%
Bukowiecka−Matusiak M, 2018	32	1.90	0.5200	11	1.60	0.2200	0.63	[−0.07; 1.33]	0.1%	0.3%
Bagci H, 2018	40	1.61	0.3200	40	1.53	0.3100	0.25	[−0.19; 0.69]	0.1%	0.4%
Khosrowbeygi A, 2018	40	0.90	0.4400	40	1.35	0.5100	−0.94	[−1.40; −0.47]	0.1%	0.4%
Franzago M, 2018	104	1.84	0.4900	124	1.84	0.4700	0.00	[−0.26; 0.26]	0.4%	0.5%
Al−Ajlan A, 2018	116	1.30	0.3000	303	1.30	0.3000	0.00	[−0.21; 0.21]	0.6%	0.5%
Al−Daghri NM, 2018	63	1.30	0.4000	54	1.20	0.3000	0.28	[−0.09; 0.64]	0.2%	0.4%
Bao W, 2018	107	1.67	0.6200	214	1.87	0.0400	−0.56	[−0.79; −0.32]	0.5%	0.5%
Siddiqui K, 2017	14	1.44	0.4200	21	1.28	0.2500	0.48	[−0.21; 1.16]	0.1%	0.3%
Cao W, 2018	33	1.57	0.0400	33	2.05	0.0500	−10.48	[−12.38; −8.57]	0.0%	0.1%
Yue CY, 2018	88	1.38	0.1900	456	1.38	0.2100	0.00	[−0.23; 0.23]	0.5%	0.5%
Tuzun D, 2018	54	1.70	0.5600	33	1.71	0.2700	−0.02	[−0.45; 0.41]	0.1%	0.4%
Siddiqui K, 2019	44	1.23	0.2800	48	1.56	0.4600	−0.85	[−1.28; −0.42]	0.2%	0.4%
Demi E, 2019	85	1.72	0.4300	90	1.77	0.4700	−0.11	[−0.41; 0.19]	0.3%	0.5%
Li S, 2018	90	1.88	0.7100	90	1.89	0.3800	−0.02	[−0.31; 0.27]	0.3%	0.5%
de la Torre NG, 2019	130	1.66	0.3900	802	1.64	0.3900	0.05	[−0.13; 0.24]	0.8%	0.5%
Pezeshki B, 2019	30	1.22	0.2100	301	1.10	0.1500	0.77	[0.39; 1.15]	0.2%	0.4%
Wu, 2019	40	1.83	0.3200	40	1.91	0.2200	−0.29	[−0.73; 0.15]	0.1%	0.4%
Kang, 2019	72	1.82	0.4700	100	1.84	0.3300	−0.05	[−0.35; 0.25]	0.3%	0.5%
Huang, 2019	33	1.77	0.3400	293	1.74	0.3000	0.10	[−0.26; 0.46]	0.2%	0.4%
Alhabri, 2019	200	0.90	0.4000	200	0.90	0.2000	0.00	[−0.20; 0.20]	0.7%	0.5%
Anjum, 2019	25	1.27	0.2200	50	1.61	0.4000	−0.96	[−1.46; −0.45]	0.1%	0.4%
Layton, 2018	67	2.20	0.5600	739	1.90	0.4100	0.71	[0.45; 0.96]	0.4%	0.5%
Wang, 2019	300	1.75	0.3300	1283	1.89	0.3500	−0.40	[−0.53; −0.28]	1.8%	0.5%
Abo−Elmatty, 2018	85	1.16	0.2000	80	1.40	0.3100	−0.92	[−1.24; −0.60]	0.3%	0.4%
Abo−Elmatty, 2018	85	1.20	0.2200	80	1.40	0.3100	−0.74	[−1.06; −0.43]	0.3%	0.5%
Alyas S, 2019	58	0.87	0.3800	100	1.08	0.2000	−0.75	[−1.08; −0.41]	0.3%	0.4%
Benhalima K, 2019	189	1.90	0.3700	1113	1.90	0.3700	0.00	[−0.15; 0.15]	1.2%	0.5%
Abd KI, 2020	45	0.96	0.1800	45	1.17	0.2900	−0.86	[−1.30; −0.43]	0.1%	0.4%
Bulmus F, 2020	30	1.60	0.4100	30	1.84	0.4400	−0.56	[−1.07; −0.04]	0.1%	0.4%
Jack−Roberts C, 2020	33	1.62	0.4400	29	1.74	0.2900	−0.31	[−0.82; 0.19]	0.1%	0.4%
Khosrowbeygi A, 2020	49	0.83	0.4200	51	1.37	0.4300	−1.26	[−1.69; −0.83]	0.2%	0.4%
Liu Y, 2020	30	1.89	0.3200	30	1.92	0.3100	−0.09	[−0.60; 0.41]	0.1%	0.4%
Mosavat M, 2020	53	2.05	0.4400	43	2.26	0.4600	−0.46	[−0.87; −0.06]	0.2%	0.4%
Mohammed Ali D, 2020	60	1.13	0.1300	30	1.34	0.1000	−1.72	[−2.23; −1.21]	0.1%	0.4%
Sun M, 2020	64	1.78	0.2600	128	1.71	0.1300	0.38	[0.08; 0.68]	0.3%	0.5%
Yavuzkir S, 2020	30	1.28	0.2800	30	1.44	0.3300	−0.52	[−1.03; 0.00]	0.1%	0.4%
Zhong L, 2020	40	1.92	0.3800	40	1.93	0.3500	−0.03	[−0.47; 0.41]	0.1%	0.4%
Liu L, 2020	61	1.35	0.4400	122	1.43	0.4000	−0.19	[−0.50; 0.12]	0.3%	0.5%
Contreras−Duarte S, 2020	69	1.81	0.4700	41	2.05	0.5700	−0.47	[−0.86; −0.08]	0.2%	0.4%
Contreras−Duarte S, 2020	48	1.71	0.4300	41	2.05	0.5700	−0.68	[−1.10; −0.25]	0.2%	0.4%
Gülcü Bulmus F, 2020	30	1.61	0.4100	30	1.84	0.4400	−0.53	[−1.05; −0.02]	0.1%	0.4%
O'Malley E, 2020	108	1.80	0.5000	94	1.90	0.3000	−0.24	[−0.52; 0.04]	0.4%	0.5%
Liu T, 2020	300	1.73	0.5100	261	1.83	0.4300	−0.21	[−0.38; −0.04]	1.0%	0.5%
Lu W, 2021	199	2.02	0.4200	199	2.05	0.3700	−0.08	[−0.27; 0.12]	0.7%	0.5%
Mei J, 2021	104	1.09	0.1900	104	1.33	0.2500	−1.08	[−1.37; −0.79]	0.3%	0.5%
Meng X, 2021	34	1.78	0.4100	34	2.09	0.4200	−0.74	[−1.23; −0.25]	0.1%	0.4%
Sudana N, 2021	25	1.42	0.2700	50	1.55	0.3100	−0.43	[−0.92; 0.05]	0.1%	0.4%
Raczkowska B, 2021	26	1.96	0.6900	29	1.89	0.5500	0.11	[−0.42; 0.64]	0.1%	0.4%
Ramasammy R, 2021	210	1.46	0.2500	209	1.38	0.2300	0.33	[0.14; 0.53]	0.8%	0.5%
Ramanjaneya M, 2021a	34	1.30	0.3000	16	1.60	0.4000	−0.88	[−1.50; −0.26]	0.1%	0.3%
Ramanjaneya M, 2021b	46	1.40	0.3000	26	1.50	0.4000	−0.29	[−0.78; 0.19]	0.1%	0.4%
Kapustin V, 2021	88	1.78	0.1500	38	2.04	0.5800	−0.76	[−1.15; −0.37]	0.2%	0.4%
Kapustin V, 2021	42	1.55	0.1600	38	2.04	0.5800	−1.17	[−1.64; −0.69]	0.1%	0.4%
Zhang L, 2021	36	1.86	0.4400	38	1.83	0.3500	0.07	[−0.38; 0.53]	0.1%	0.4%
Rakchna M, 2021	73	1.35	0.2500	72	1.40	0.2700	−0.19	[−0.52; 0.14]	0.3%	0.4%
Baryla I, 2021	98	2.02	0.4400	37	2.07	0.5900	−0.10	[−0.48; 0.28]	0.2%	0.4%
Abd−Eltawab A, 2020	109	1.13	0.1800	103	1.17	0.1400	−0.25	[−0.52; 0.02]	0.4%	0.5%
Ren M, 2021	56	1.53	0.1800	56	1.69	0.2200	−0.79	[−1.18; −0.41]	0.2%	0.4%
Xu D, 2021	80	1.73	0.4700	80	2.07	0.6200	−0.62	[−0.93; −0.30]	0.3%	0.5%
Benhalima K, 2021	97	1.90	0.4400	327	2.00	0.3700	−0.26	[−0.49; −0.03]	0.5%	0.5%
Tabacu C, 2021	16	1.63	0.5000	83	1.80	0.3800	−0.42	[−0.96; 0.12]	0.1%	0.4%
Eppel D, 2021	27	1.71	0.4200	40	2.08	0.3700	−0.94	[−1.45; −0.42]	0.1%	0.4%
Onat T, 2021	60	1.26	0.1800	52	1.58	0.2400	−1.51	[−1.94; −1.09]	0.2%	0.4%
Wang Y, 2021	855	1.50	0.4000	785	1.80	0.3000	−0.84	[−0.94; −0.74]	2.7%	0.5%
Bulut A, 2021	22	1.83	0.3100	29	2.13	0.4800	−0.71	[−1.28; −0.14]	0.1%	0.4%
Yu X, 2021	573	1.61	0.3700	740	1.66	0.4100	−0.13	[−0.24; −0.02]	2.4%	0.5%
Shalabi T, 2021	114	1.45	0.1000	114	1.55	0.1000	−1.00	[−1.27; −0.72]	0.4%	0.5%
Adiga U, 2021	100	1.31	0.3100	100	1.39	0.3100	−0.26	[−0.54; 0.02]	0.4%	0.5%
Cheng E, 2022	163	1.66	0.4700	145	1.70	0.5400	−0.08	[−0.30; 0.14]	0.6%	0.5%
Huang G, 2022	566	1.66	0.4200	710	1.65	0.3900	0.02	[−0.09; 0.14]	2.3%	0.5%
Juchnicka I, 2022	24	2.20	0.2400	24	2.50	0.6000	−0.65	[−1.23; −0.06]	0.1%	0.4%
Liu L, 2021	69	1.52	0.5600	71	1.65	0.6900	−0.21	[−0.54; 0.13]	0.3%	0.4%
Obaidullah M, 2021	132	1.02	0.1900	100	1.13	0.1700	−0.60	[−0.87; −0.34]	0.4%	0.5%
Adiga U, 2022	74	1.39	0.3000	132	1.37	0.3300	0.06	[−0.22; 0.35]	0.3%	0.5%
Shen L, 2023	233	2.06	0.4200	1001	2.08	0.3600	−0.05	[−0.20; 0.09]	1.4%	0.5%
Sahoo D, 2022	20	1.06	0.0300	45	1.13	0.0400	−1.86	[−2.48; −1.23]	0.1%	0.3%
Arslan A, 2022	40	1.39	0.3200	37	1.47	0.3200	−0.25	[−0.70; 0.20]	0.1%	0.4%
Hu W, 2022	315	1.88	0.4600	1876	2.06	0.5000	−0.36	[−0.48; −0.24]	2.0%	0.5%
Wu Y, 2022	130	2.61	0.5200	170	2.95	0.6200	−0.59	[−0.82; −0.35]	0.5%	0.5%
Cibickova L, 2022	43	2.00	0.5300	19	2.20	0.2800	−0.42	[−0.97; 0.12]	0.1%	0.4%
Karagoz ZK, 2022	30	1.49	0.2400	30	1.60	0.2900	−0.41	[−0.92; 0.10]	0.1%	0.4%
Wang, Y 2022	232	2.15	0.4500	696	2.36	0.4500	−0.47	[−0.62; −0.32]	1.2%	0.5%
Jia X, 2022	62	1.75	0.4100	58	2.05	0.4400	−0.70	[−1.07; −0.33]	0.2%	0.4%
Zeljkovic A, 2022	15	2.11	0.3900	48	2.10	0.3900	0.03	[−0.55; 0.61]	0.1%	0.4%
Wang J, 2023	47	1.48	0.3000	47	1.48	0.2900	0.00	[−0.40; 0.40]	0.2%	0.4%
Ma, 2023	110	1.51	0.3000	110	1.64	0.3600	−0.39	[−0.66; −0.12]	0.4%	0.5%
Akash, 2023	400	1.21	0.2400	100	1.42	0.2200	−0.89	[−1.11; −0.66]	0.5%	0.5%
Hou, 2021	189	2.03	0.1400	100	2.22	0.2100	−1.13	[−1.39; −0.87]	0.4%	0.5%
Kagitci, 2023	30	1.58	0.4600	30	1.80	0.3600	−0.53	[−1.04; −0.01]	0.1%	0.4%
Gawlik, 2023	74	1.78	0.2900	32	1.93	0.3100	−0.50	[−0.92; −0.08]	0.2%	0.4%
Burzynska−Pedziwiatr, 2023	32	1.92	0.5500	11	1.59	0.2500	0.66	[−0.04; 1.36]	0.1%	0.3%
Shi, 2023	1632	1.79	0.5500	9067	2.11	0.5000	−0.63	[−0.68; −0.58]	9.9%	0.5%
Li, 2023	148	2.03	0.4800	296	2.13	0.4700	−0.21	[−0.41; −0.01]	0.7%	0.5%
Ali Khan, 2023	90	0.71	0.3600	90	0.92	0.4400	−0.52	[−0.82; −0.22]	0.3%	0.5%
Linares−Pineda, 2023	16	2.01	0.4100	16	2.14	0.4700	−0.29	[−0.98; 0.41]	0.1%	0.3%
Shen, 2024	12	2.01	0.3100	10	1.77	0.3800	0.67	[−0.19; 1.54]	0.0%	0.3%
Thilak, 2024	60	0.99	0.2300	60	0.98	0.2800	0.04	[−0.32; 0.40]	0.2%	0.4%
Özer, 2024	44	1.32	0.2800	44	1.47	0.2800	−0.53	[−0.96; −0.11]	0.2%	0.4%
Ghaneialvar, 2024	40	1.19	0.2900	40	1.27	0.3500	−0.25	[−0.69; 0.19]	0.1%	0.4%
Aziz, 2024	50	0.52	0.0800	50	0.74	0.0800	−2.73	[−3.28; −2.18]	0.1%	0.4%
Uysal, 2024	52	1.58	1.5400	31	1.67	0.4000	−0.07	[−0.52; 0.37]	0.1%	0.4%
Wu, 2024	35	2.13	0.3600	70	2.02	0.3800	0.29	[−0.12; 0.70]	0.2%	0.4%
Li, 2024	1323	1.48	0.2600	1421	1.48	0.2600	0.00	[−0.07; 0.07]	5.0%	0.5%
Ademoglu EN, 2015	47	1.60	0.5000	32	1.90	0.4000	−0.64	[−1.10; −0.18]	0.1%	0.4%
Anghebem−Oliveira MI, 2017	127	1.47	0.4300	125	1.67	0.5200	−0.42	[−0.67; −0.17]	0.5%	0.5%
Boghossian NS, 2017	93	1.48	0.3000	136	1.64	0.3400	−0.49	[−0.76; −0.22]	0.4%	0.5%
Dudzik D, 2014	20	2.23	0.4200	20	2.13	0.4500	0.23	[−0.40; 0.85]	0.1%	0.3%
Dudzik D, 2017	24	2.21	0.4500	24	2.37	0.4000	−0.37	[−0.94; 0.20]	0.1%	0.4%
Yorulmaz Ertug E, 2016	29	1.66	0.3400	20	1.79	0.3300	−0.38	[−0.96; 0.19]	0.1%	0.4%
Guimarães LO,2014	150	1.47	0.3900	295	1.37	0.4200	0.24	[0.05; 0.44]	0.7%	0.5%
Inegol Gumus I, 2013	34	1.34	0.3000	50	1.45	0.3500	−0.33	[−0.77; 0.11]	0.1%	0.4%
Hollingsworth DR, 1982	5	1.14	0.2500	21	1.54	0.3000	−1.33	[−2.38; −0.27]	0.0%	0.2%
Knopp RH, 1980	22	1.10	0.2300	38	1.33	0.2400	−0.96	[−1.51; −0.41]	0.1%	0.4%
Liang Z, 2015	38	1.90	0.4000	35	2.40	0.8000	−0.79	[−1.27; −0.31]	0.1%	0.4%
Liu B, 2016	276	1.82	0.3500	1270	1.85	0.3300	−0.09	[−0.22; 0.04]	1.7%	0.5%
Liu B, 2016	28	1.67	0.3800	25	1.67	0.3600	0.00	[−0.54; 0.54]	0.1%	0.4%
Rojas I, 2002	38	1.87	0.4600	38	2.00	0.5300	−0.26	[−0.71; 0.19]	0.1%	0.4%
Whyte K, 2013	22	1.39	0.3500	167	1.54	0.4100	−0.37	[−0.82; 0.08]	0.1%	0.4%
Anderlová K, 2022	36	2.21	0.4800	22	2.31	0.5200	−0.20	[−0.73; 0.33]	0.1%	0.4%
Attique H, 2022	37	1.38	0.2500	47	1.41	0.2300	−0.12	[−0.56; 0.31]	0.2%	0.4%
Beunen K, 2014	231	1.50	0.2000	1612	1.60	0.2000	−0.50	[−0.64; −0.36]	1.5%	0.5%
Chen Y, 2017	28	2.06	0.4500	56	2.19	0.4400	−0.29	[−0.75; 0.17]	0.1%	0.4%
Diboun I, 2020	32	1.30	0.3000	21	1.60	0.4000	−0.86	[−1.44; −0.29]	0.1%	0.4%
Franzago M, 2020	50	1.50	0.3000	50	1.70	0.2000	−0.78	[−1.19; −0.37]	0.2%	0.4%
Grieger J, 2021	103	1.60	0.3000	2812	1.80	0.4000	−0.50	[−0.70; −0.31]	0.7%	0.5%
Guan L, 2021	461	1.98	0.4700	626	2.00	0.4500	−0.04	[−0.16; 0.08]	1.9%	0.5%
Guo Y, 2020	87	1.90	0.5200	88	2.00	0.3700	−0.22	[−0.52; 0.08]	0.3%	0.5%
Huo Y, 2019	30	1.75	0.4400	28	1.94	0.3900	−0.45	[−0.97; 0.07]	0.1%	0.4%
Iimura Y, 2015	53	2.10	0.3000	1108	2.14	0.4000	−0.10	[−0.38; 0.17]	0.4%	0.5%
Kralisch S, 2009	40	1.70	0.7000	80	1.90	0.5000	−0.35	[−0.73; 0.04]	0.2%	0.4%
Li J, 2015	35	1.80	0.4000	43	1.70	0.4000	0.25	[−0.20; 0.70]	0.1%	0.4%
Li J, 2017	100	1.62	0.5100	100	1.53	0.3700	0.20	[−0.08; 0.48]	0.4%	0.5%
Li F, 2020	48	2.09	0.9400	202	1.96	0.6100	0.19	[−0.13; 0.50]	0.3%	0.5%
Li Y, 2021	359	1.86	0.4200	273	2.02	0.4100	−0.38	[−0.54; −0.23]	1.1%	0.5%
Lu L, 2023	83	1.33	0.4200	649	2.15	0.7100	−1.20	[−1.43; −0.96]	0.5%	0.5%
Ma J, 2022	31	1.31	0.2400	69	1.28	0.3000	0.11	[−0.32; 0.53]	0.2%	0.4%
Meyer B, 2023	43	1.61	0.4600	26	1.65	0.5200	−0.08	[−0.57; 0.41]	0.1%	0.4%
Onat T, 2021	40	1.20	0.1200	40	1.53	0.1500	−2.41	[−2.99; −1.82]	0.1%	0.4%
Qin L, 2023	82	1.90	0.3400	89	2.00	0.3300	−0.30	[−0.60; 0.00]	0.3%	0.5%
Sun J, 2022	80	1.82	0.3600	79	1.87	0.3600	−0.14	[−0.45; 0.17]	0.3%	0.5%
Takhshid M, 2015	75	1.36	0.3500	70	1.36	0.3200	0.00	[−0.33; 0.33]	0.3%	0.4%
Theodorakopoulou M, 2021	21	1.70	0.2200	15	1.90	0.3000	−0.76	[−1.45; −0.08]	0.1%	0.3%
Tönjes A, 2019	74	1.85	0.4700	74	1.90	0.3800	−0.12	[−0.44; 0.21]	0.3%	0.4%
Tutar D, 2022	40	1.54	0.2600	40	1.68	0.3300	−0.47	[−0.91; −0.02]	0.1%	0.4%
Wang D, 2015	110	1.81	0.4400	526	1.88	0.3500	−0.19	[−0.40; 0.02]	0.7%	0.5%
Wang X, 2015	839	1.96	0.3900	900	2.09	0.4200	−0.32	[−0.41; −0.23]	3.1%	0.5%
Wang H, 2019	35	1.73	0.2000	35	2.06	0.3000	−1.28	[−1.80; −0.76]	0.1%	0.4%
Wang H, 2019	35	1.61	0.3000	35	1.91	0.4000	−0.84	[−1.33; −0.35]	0.1%	0.4%
Wójcik M, 2015	77	1.81	0.5000	34	1.99	0.4000	−0.38	[−0.79; 0.03]	0.2%	0.4%
Xu L, 2013	120	2.02	0.4200	60	2.22	0.3500	−0.50	[−0.81; −0.19]	0.3%	0.5%
Xu M, 2015	55	1.61	0.4000	66	1.63	0.3600	−0.05	[−0.41; 0.31]	0.2%	0.4%
Yakout S, 2023	46	1.33	0.4000	77	1.34	0.3000	−0.03	[−0.39; 0.34]	0.2%	0.4%
Yi J, 2018	141	1.67	0.2900	230	1.67	0.2900	0.00	[−0.21; 0.21]	0.6%	0.5%
Zhang Y, 2015	186	1.43	0.8100	330	1.55	0.6900	−0.16	[−0.34; 0.02]	0.9%	0.5%
Zhang H, 2022	110	2.65	0.5600	416	2.80	0.5800	−0.26	[−0.47; −0.05]	0.6%	0.5%
**Fixed effect model**	**24938**			**50960**			**−0.29**	**[−0.30; −0.27]**	**100.0%**	**−−**
**Random effects model**							**−0.31**	**[−0.38; −0.25]**	**−−**	**100.0%**

Heterogeneity: $I^2 = 92\%$, $\tau^2 = 0.1935$, $p = 0$

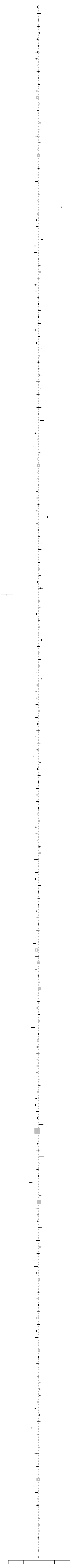

Supplement: Supplementary file 1 [file medicina-61-01290-s001.zip › Figure S29 HDL 2nd trimester.pdf]
