# Supplementary material for: Trimester-Specific Serum Lipid Profiles in Gestational Diabetes Mellitus: A Systematic Review, Meta-Analysis, and Meta-Regression
Source: Medicina (Kaunas). 2025 Jul 17;61(7):1290. doi: 10.3390/medicina61071290 (PMC12300116; doi:10.3390/medicina61071290)

Study	Experimental Total	Experimental Mean	Experimental SD	Control Total	Control Mean	Control SD	SMD	95%−CI	Weight (fixed)	Weight (random)
Koukkou E, 1997	20	1.71	0.4000	22.000	1.72	0.3000	−0.03	[−0.63; 0.58]	0.2%	0.9%
Couch S, 1998	25	1.80	0.4700	25.000	1.99	0.5100	−0.38	[−0.94; 0.18]	0.2%	1.0%
Bartha J, 2000	34	2.04	0.5100	32.000	1.55	0.6200	0.86	[0.35; 1.36]	0.3%	1.0%
Paradisi G, 2002	13	1.68	0.2300	15.000	1.52	2.5600	0.08	[−0.66; 0.83]	0.1%	0.9%
Ranheim T, 2004	22	1.60	0.3800	29.000	1.80	0.4900	−0.44	[−1.00; 0.12]	0.2%	1.0%
Tsai P, 2005	34	1.60	0.3000	219.000	1.60	0.4000	0.00	[−0.36; 0.36]	0.6%	1.0%
Bartha J, 2008	30	2.12	0.4600	20.000	1.83	0.5600	0.57	[−0.01; 1.15]	0.2%	0.9%
Szymanska M, 2008	81	2.12	0.3600	41.000	1.99	0.3800	0.35	[−0.03; 0.73]	0.5%	1.0%
Akturk M, 2008	47	1.88	0.4100	31.000	1.21	0.3300	1.74	[1.21; 2.28]	0.3%	1.0%
Pfau D, 2010	40	1.70	0.7000	80.000	1.90	0.5000	−0.35	[−0.73; 0.04]	0.5%	1.0%
Paradisi G, 2010	12	1.70	0.6200	38.000	2.04	1.6000	−0.23	[−0.88; 0.42]	0.2%	0.9%
Akturk M, 2010	54	1.88	0.3700	69.000	1.99	0.5800	−0.22	[−0.58; 0.14]	0.6%	1.0%
Retnakaran R, 2010	136	1.53	0.2900	87.000	1.63	0.3600	−0.31	[−0.58; −0.04]	1.0%	1.0%
Culha C, 2011	24	1.42	0.3600	20.000	1.60	0.1400	−0.63	[−1.23; −0.02]	0.2%	0.9%
Ghafoor S, 2012	46	0.72	0.3400	50.000	1.00	0.8500	−0.42	[−0.83; −0.02]	0.5%	1.0%
Farhan S, 2012	10	1.81	0.2800	10.000	1.96	1.7100	−0.12	[−0.99; 0.76]	0.1%	0.8%
Cocelli V, 2012	62	1.32	0.2600	61.000	1.70	0.3400	−1.25	[−1.64; −0.86]	0.5%	1.0%
Gkiomisi A, 2013	44	1.66	0.4000	44.000	1.80	0.4000	−0.35	[−0.77; 0.07]	0.4%	1.0%
Khan R, 2013	103	1.42	0.2100	97.000	2.25	0.4100	−2.56	[−2.94; −2.19]	0.5%	1.0%
dos Santos−Weiss I, 2012	288	1.50	0.3000	288.000	1.60	0.4000	−0.28	[−0.45; −0.12]	2.8%	1.1%
Soydinc S, 2013	42	1.57	0.3100	33.000	1.39	0.3100	0.57	[0.11; 1.04]	0.3%	1.0%
Kärkkäinen H, 2013	42	1.92	0.2200	32.000	1.99	0.1300	−0.37	[−0.84; 0.09]	0.3%	1.0%
Agakidou E, 2013	27	1.67	0.4100	27.000	1.84	0.4800	−0.38	[−0.91; 0.16]	0.3%	1.0%
Eslamian R, 2013	112	1.40	0.1200	159.000	1.42	0.0800	−0.20	[−0.44; 0.04]	1.3%	1.0%
Eslamian R, 2013	112	1.54	0.1600	159.000	1.54	0.1600	0.00	[−0.24; 0.24]	1.3%	1.0%
Yousefzadeh G, 2013	60	1.18	0.2000	30.000	1.06	0.3100	0.49	[0.05; 0.94]	0.4%	1.0%
Al−Hakeem M, 2014	200	0.92	0.3800	300.000	0.64	0.2400	0.92	[0.73; 1.11]	2.1%	1.1%
Wójcik M, 2014	132	1.81	0.4600	43.000	1.99	0.4100	−0.40	[−0.75; −0.05]	0.6%	1.0%
Al Rubeaan, 2014	201	1.07	0.3200	328.000	1.15	0.3200	−0.25	[−0.43; −0.07]	2.4%	1.1%
Megia, 2014	79	1.86	0.3400	78.000	1.90	0.3300	−0.12	[−0.43; 0.19]	0.8%	1.0%
Du M, 2015	38	1.59	0.3800	38.000	2.11	0.4500	−1.24	[−1.73; −0.74]	0.3%	1.0%
Zhang Y, 2016	40	1.71	0.3200	240.000	1.76	0.4000	−0.13	[−0.46; 0.21]	0.7%	1.0%
Savona−Ventura C, 2016	459	1.80	0.5000	603.000	1.70	0.5000	0.20	[0.08; 0.32]	5.1%	1.1%
Yang X, 2017	19	2.03	0.3100	20.000	2.07	0.3200	−0.12	[−0.75; 0.50]	0.2%	0.9%
Zhang Y, 2017	50	1.71	1.0100	50.000	1.73	0.5700	−0.02	[−0.42; 0.37]	0.5%	1.0%
Burlina S, 2016	21	1.92	0.3900	21.000	2.22	0.4300	−0.72	[−1.34; −0.09]	0.2%	0.9%
Hussain Z, 2018	60	0.84	0.4900	60.000	1.74	1.1000	−1.05	[−1.43; −0.67]	0.5%	1.0%
Yuan X, 2018	86	1.44	0.2600	273.000	1.48	0.2600	−0.15	[−0.40; 0.09]	1.3%	1.0%
Zhang Y, 2018	50	1.70	0.3000	47.000	1.62	0.3400	0.25	[−0.15; 0.65]	0.5%	1.0%
Bao W, 2018	107	1.39	0.0500	214.000	1.66	0.0500	−5.39	[−5.87; −4.91]	0.3%	1.0%
Bugatto F, 2018	22	1.69	0.4800	23.000	1.83	0.5800	−0.26	[−0.84; 0.33]	0.2%	0.9%
Al−Daghri NM, 2019	39	1.40	0.4000	63.000	1.60	0.4000	−0.50	[−0.90; −0.09]	0.5%	1.0%
Wu, 2019	65	1.78	0.3400	65.000	1.95	0.4100	−0.45	[−0.80; −0.10]	0.6%	1.0%
Kang, 2019	72	2.14	0.5500	100.000	2.07	0.3800	0.15	[−0.15; 0.46]	0.8%	1.0%
Wang, 2019	300	1.64	0.3000	1283.000	1.74	0.3300	−0.31	[−0.43; −0.18]	4.7%	1.1%
Aydemir B, 2019	99	1.62	0.3500	98.000	1.61	0.3200	0.03	[−0.25; 0.31]	1.0%	1.0%
Fan Y, 2020	65	1.23	0.1400	55.000	1.86	0.2300	−3.35	[−3.92; −2.79]	0.2%	1.0%
Mohammed Ali D, 2020	60	1.06	0.0800	30.000	1.31	0.1000	−2.85	[−3.46; −2.24]	0.2%	0.9%
Contreras−Duarte S, 2020	69	1.70	0.4900	41.000	1.98	0.4800	−0.57	[−0.97; −0.18]	0.5%	1.0%
Contreras−Duarte S, 2020	48	1.68	0.4500	41.000	1.98	0.4800	−0.64	[−1.07; −0.21]	0.4%	1.0%
Liu M, 2020	50	1.63	0.2700	47.000	1.83	0.3700	−0.62	[−1.02; −0.21]	0.5%	1.0%
Li G, 2021	23	1.81	0.2900	29.000	2.10	0.3400	−0.90	[−1.47; −0.32]	0.2%	0.9%
Hussain Z, 2021	60	0.75	0.4700	60.000	1.67	0.8500	−1.33	[−1.73; −0.93]	0.5%	1.0%
Wu L, 2021	213	1.82	0.3700	191.000	1.85	0.3600	−0.08	[−0.28; 0.11]	2.0%	1.1%
Zhou J, 2021	50	1.75	0.3100	50.000	2.10	0.4100	−0.96	[−1.37; −0.54]	0.4%	1.0%
Wang F, 2021	53	1.71	0.2900	46.000	1.97	0.3400	−0.82	[−1.23; −0.41]	0.4%	1.0%
Balachandiran M, 2021	40	1.23	0.2700	40.000	1.15	0.3000	0.28	[−0.16; 0.72]	0.4%	1.0%
Abdualhay R, 2022	44	0.89	0.1700	45.000	0.93	0.2300	−0.20	[−0.61; 0.22]	0.4%	1.0%
Franzago M, 2022	33	1.83	0.4300	27.000	1.70	0.5100	0.27	[−0.24; 0.79]	0.3%	1.0%
Dualib P, 2022	56	1.60	0.3800	59.000	1.62	0.3800	−0.05	[−0.42; 0.31]	0.6%	1.0%
Yang J, 2022	21	1.50	0.3000	60.000	1.81	0.3500	−0.91	[−1.43; −0.39]	0.3%	1.0%
Parveen S, 2022	37	1.02	0.1500	163.000	1.05	0.1500	−0.20	[−0.56; 0.16]	0.6%	1.0%
Mahmood K, 2022	50	1.17	0.2700	50.000	1.22	0.3100	−0.17	[−0.56; 0.22]	0.5%	1.0%
Sobczynska−Malefora A, 2021	24	1.50	0.3000	35.000	1.40	0.3000	0.33	[−0.19; 0.85]	0.3%	1.0%
Algaba−Chueca F, 2022	62	1.86	0.3900	74.000	1.89	0.3400	−0.08	[−0.42; 0.26]	0.7%	1.0%
Bernea E, 2022	10	1.91	0.4600	8.000	1.96	0.6100	−0.09	[−1.02; 0.84]	0.1%	0.8%
Zeljkovic A, 2022	15	1.73	0.3300	48.000	2.08	0.5600	−0.67	[−1.26; −0.08]	0.2%	0.9%
Zheng W, 2022	396	1.72	0.3300	2789.000	1.84	0.3500	−0.35	[−0.45; −0.24]	6.7%	1.1%
Wang J, 2023	47	1.76	0.3100	47.000	1.87	0.4400	−0.29	[−0.69; 0.12]	0.5%	1.0%
Akhtar, 2023	100	2.76	0.3500	100.000	3.53	0.5700	−1.62	[−1.94; −1.30]	0.7%	1.0%
Visiedo, 2023	20	1.62	0.3800	17.000	1.75	0.5300	−0.28	[−0.93; 0.37]	0.2%	0.9%
Jiang, 2023	719	1.98	0.4300	1205.000	1.99	0.4100	−0.02	[−0.12; 0.07]	8.8%	1.1%
Shi, 2023	1632	1.62	0.5100	9067.000	2.45	0.5300	−1.57	[−1.63; −1.52]	23.3%	1.1%
Linares−Pineda, 2023	16	1.89	0.4300	16.000	1.93	0.3200	−0.10	[−0.80; 0.59]	0.2%	0.9%
Nahdh Saleem, 2024	30	1.06	0.2000	22.000	0.86	0.2600	0.87	[0.29; 1.44]	0.2%	0.9%
Nahdh Saleem, 2024	24	1.15	0.3100	22.000	0.86	0.2600	0.99	[0.38; 1.61]	0.2%	0.9%
Heiskanen N, 2010	51	1.80	0.5000	28.000	2.00	0.5000	−0.40	[−0.86; 0.07]	0.3%	1.0%
Hsu HW, 1997	8	1.20	0.3000	8.000	1.40	0.4000	−0.53	[−1.54; 0.47]	0.1%	0.8%
Huo Y, 2015	30	2.32	0.2300	27.000	1.40	0.2600	3.71	[2.83; 4.59]	0.1%	0.8%
Keskin FE, 2015	44	1.66	0.5300	45.000	1.80	0.5600	−0.25	[−0.67; 0.16]	0.4%	1.0%
Liao Y, 2018	16	2.16	0.4100	16.000	1.58	0.3900	1.41	[0.63; 2.20]	0.1%	0.9%
Maitland RA, 2014	29	1.64	1.3200	77.000	1.71	1.2600	−0.05	[−0.48; 0.37]	0.4%	1.0%
Maple−Brown, 2012	198	1.60	0.4000	477.000	1.70	0.4000	−0.25	[−0.42; −0.08]	2.7%	1.1%
Marseille−Tremblay C, 2018	7	1.63	0.4200	7.000	1.81	0.2400	−0.49	[−1.56; 0.58]	0.1%	0.7%
Miettinen HE, 2014	22	1.66	0.5200	30.000	1.76	0.3800	−0.22	[−0.77; 0.33]	0.2%	1.0%
Miettine HE, 2018	15	1.63	0.5400	13.000	1.73	0.3600	−0.21	[−0.95; 0.54]	0.1%	0.9%
Morimitsu LK, 2007	23	1.49	0.3200	11.000	1.46	0.4200	0.08	[−0.64; 0.80]	0.1%	0.9%
Mrizak I, 2013	28	1.31	0.3000	24.000	1.47	0.5500	−0.36	[−0.91; 0.19]	0.2%	1.0%
Mrizak I, 2014	30	2.20	0.1200	30.000	2.25	0.1600	−0.35	[−0.86; 0.16]	0.3%	1.0%
Sreckovic I, 2014	9	1.63	0.5000	11.000	1.84	0.6000	−0.36	[−1.25; 0.53]	0.1%	0.8%
Visiedo F, 2013	8	1.62	0.8700	14.000	1.95	0.9300	−0.35	[−1.23; 0.53]	0.1%	0.8%
Weng Q, 2019	113	1.67	0.4200	396.000	1.73	0.4300	−0.14	[−0.35; 0.07]	1.7%	1.1%
Zakovicova E, 2014	35	1.86	0.4000	31.000	1.80	0.3000	0.17	[−0.32; 0.65]	0.3%	1.0%
Anderlová K, 2022	36	2.24	0.4800	22.000	2.32	0.5600	−0.15	[−0.69; 0.38]	0.3%	1.0%
Barrett HL, 2021	22	1.70	0.2200	7.000	2.00	0.6700	−0.79	[−1.66; 0.09]	0.1%	0.8%
Bronisz A, 2007	121	1.52	0.3400	36.000	1.66	0.3700	−0.40	[−0.78; −0.03]	0.5%	1.0%
Franzago M, 2021	33	1.80	0.4400	27.000	1.50	0.6700	0.53	[0.02; 1.05]	0.3%	1.0%
Genova M, 2024	54	1.72	0.4300	54.000	1.73	0.4400	−0.02	[−0.40; 0.35]	0.5%	1.0%
Guo Y, 2020	87	1.80	0.3700	88.000	1.90	0.3000	−0.30	[−0.59; 0.00]	0.8%	1.0%
Ozyildirim S, 2023	87	1.18	0.2300	36.000	1.18	0.2600	0.00	[−0.39; 0.39]	0.5%	1.0%
Prakash G, 2017	139	1.13	0.1800	139.000	1.14	0.2200	−0.05	[−0.28; 0.19]	1.4%	1.0%
Radojicic O, 2024	53	1.90	0.4500	49.000	2.22	0.4800	−0.68	[−1.08; −0.28]	0.5%	1.0%
Tang F, 2023	660	1.97	0.4500	1.097	1.97	0.4200	0.00	[−1.87; 1.87]	0.0%	0.4%
**Fixed effect model**	**9684**			**21853.097**			**−0.53**	**[−0.55; −0.50]**	**100.0%**	**−−**
**Random effects model**							**−0.30**	**[−0.46; −0.13]**	**−−**	**100.0%**

Heterogeneity: $I^2 = 97\%$, $\tau^2 = 0.6616$, $p = 0$

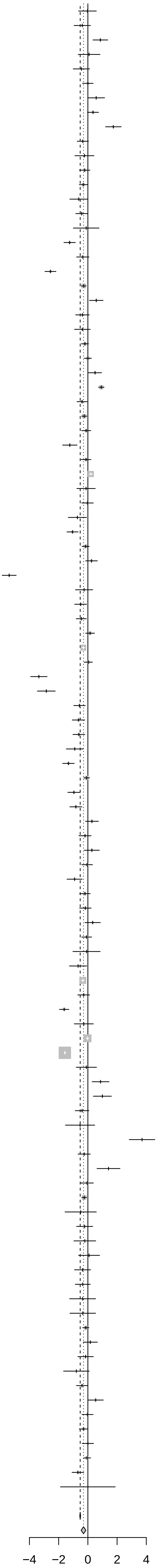

Supplement: Supplementary file 1 [file medicina-61-01290-s001.zip › Figure S30 HDL 3rd trimester.pdf]
