# Supplementary material for: Trimester-Specific Serum Lipid Profiles in Gestational Diabetes Mellitus: A Systematic Review, Meta-Analysis, and Meta-Regression
Source: Medicina (Kaunas). 2025 Jul 17;61(7):1290. doi: 10.3390/medicina61071290 (PMC12300116; doi:10.3390/medicina61071290)
Supplement: Supplementary file 1 [file medicina-61-01290-s001.zip › Figure S31 VLDL 1st trimester.pdf]

Study	Experimental Total	Experimental Mean	Experimental SD	Control Total	Control Mean	Control SD	SMD	95%−CI	Weight (fixed)	Weight (random)
Alyas S, 2019	58	1.03	0.1500	100	0.83	0.1000	1.65	[1.28; 2.02]	32.6%	25.8%
Contreras−Duarte S, 2020	69	0.64	0.3200	41	0.53	0.2000	0.39	[0.00; 0.78]	29.7%	25.7%
Abdualhay R, 2022	44	0.84	0.3100	45	0.69	0.2600	0.52	[0.10; 0.94]	25.3%	25.3%
Tunc S, 2022	12	0.61	0.1800	88	0.60	0.2100	0.05	[−0.56; 0.65]	12.4%	23.2%
**Fixed effect model**	**183**			**274**			**0.79**	**[0.58; 1.00]**	**100.0%**	**--**
**Random effects model**							**0.67**	**[−0.04; 1.38]**	**--**	**100.0%**

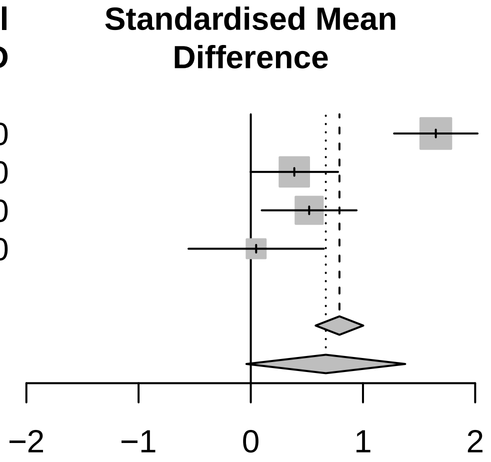

Heterogeneity: $I^2 = 91\%$, $\tau^2 = 0.4684$, $p < 0.01$
