# Supplementary material for: Trimester-Specific Serum Lipid Profiles in Gestational Diabetes Mellitus: A Systematic Review, Meta-Analysis, and Meta-Regression
Source: Medicina (Kaunas). 2025 Jul 17;61(7):1290. doi: 10.3390/medicina61071290 (PMC12300116; doi:10.3390/medicina61071290)
Supplement: Supplementary file 1 [file medicina-61-01290-s001.zip › Supplement material. Figures S1-S18. Funnel plots.docx]

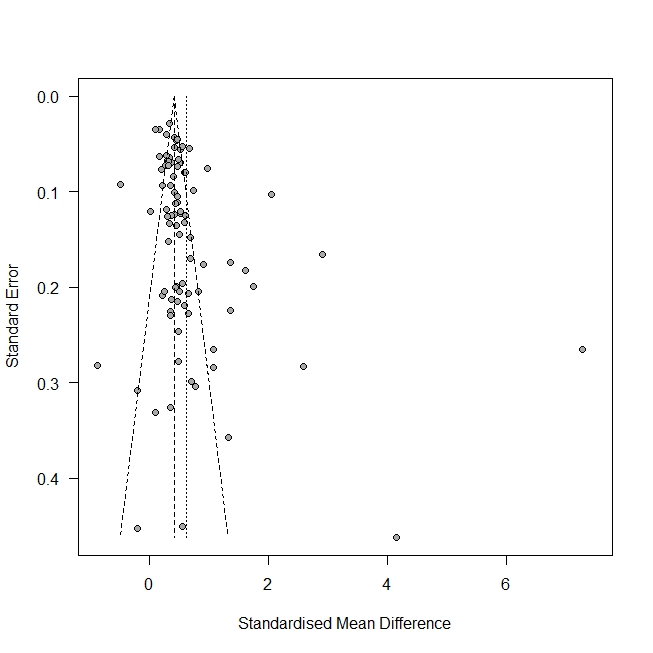


Figure S1. Funnel plot for TG in 1^st^ trimester


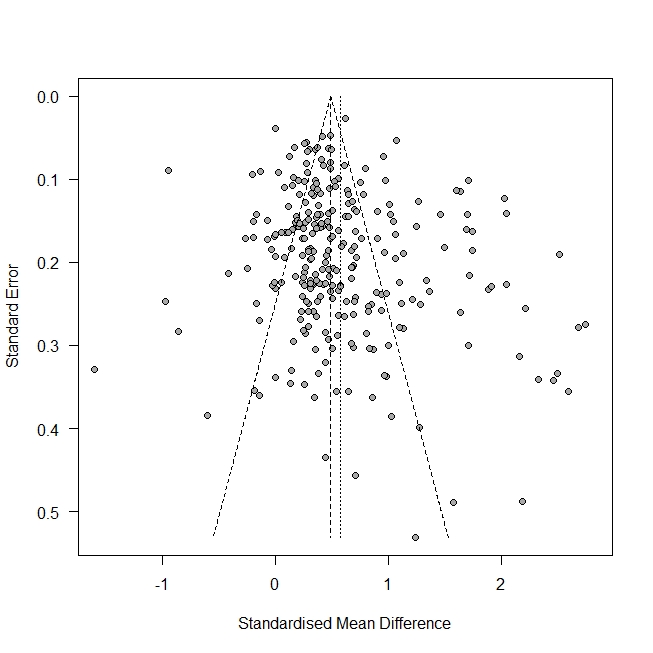


Figure S2. Funnel plot for TG in 2^nd^ trimester


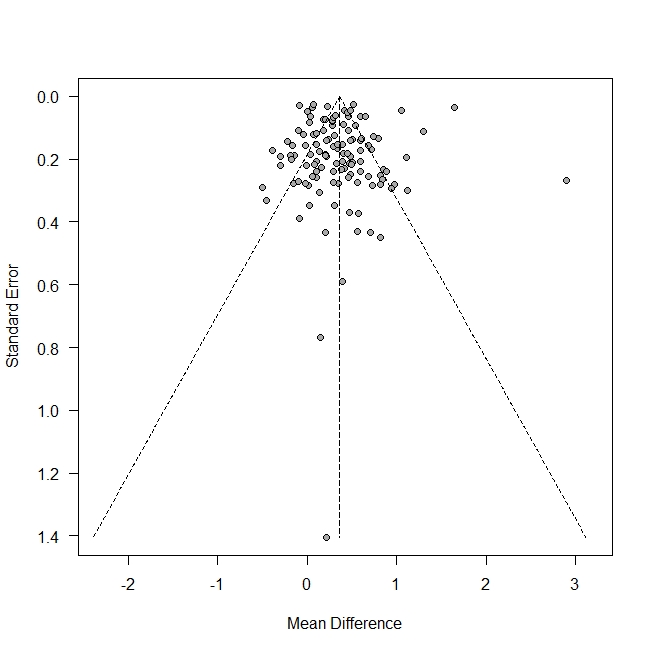


Figure S3. Funnel plot for TG in 3^rd^ trimester


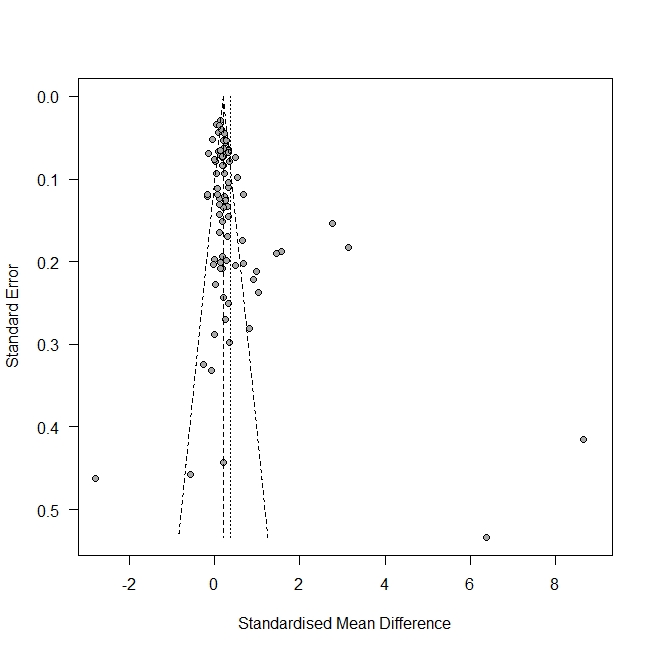


Figure S4. Funnel plot for TC in 1^st^ trimester


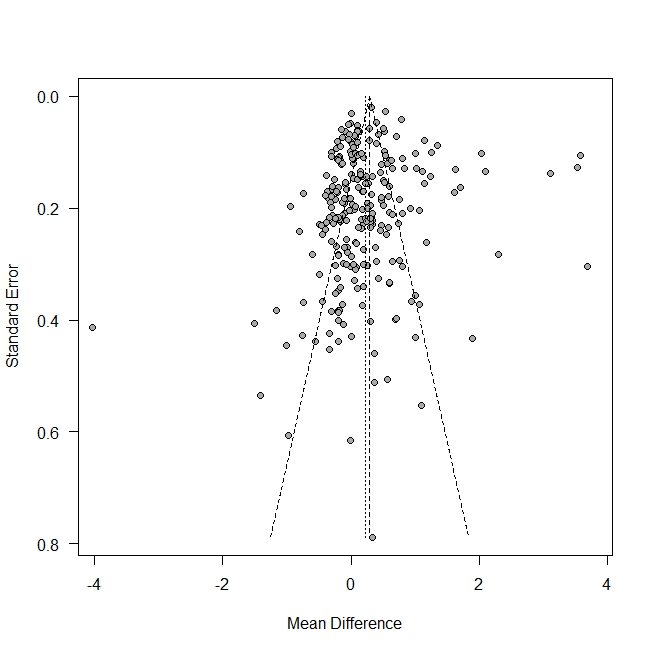


Figure S5. Funnel plot for TC in 2^nd^ trimester


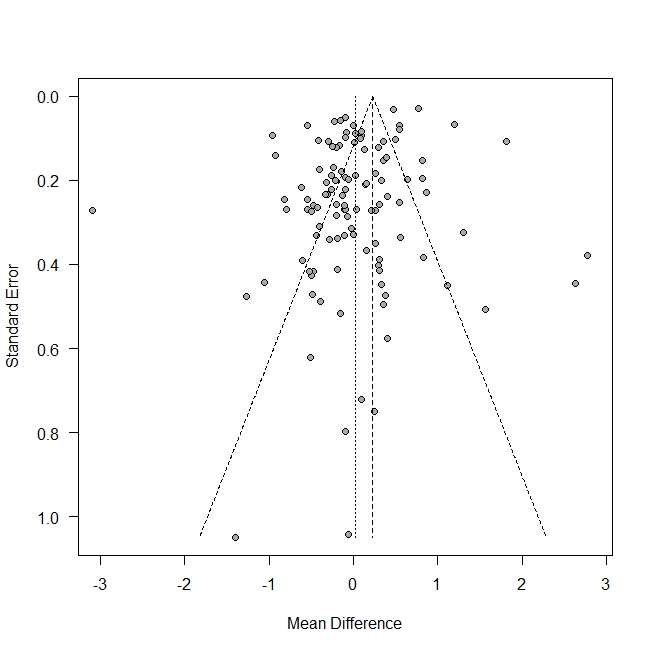


Figure S6. Funnel plot for TC in 3^rd^ trimester


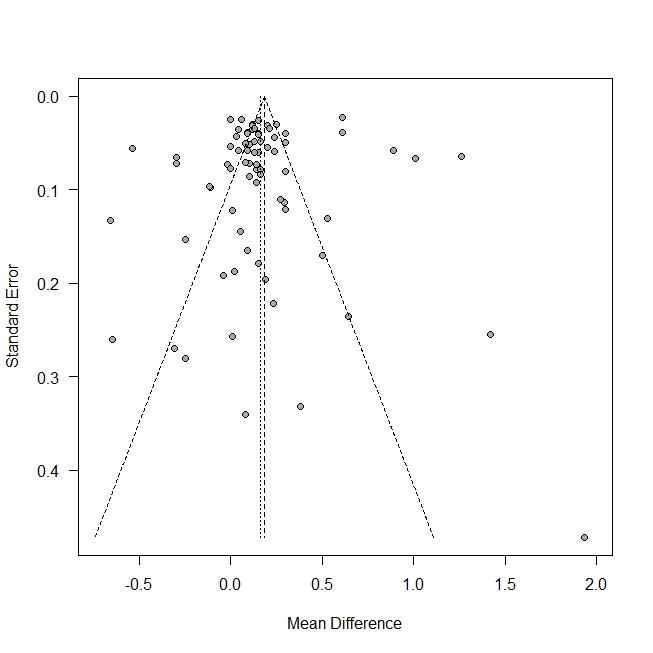


Figure S7. Funnel plot for LDL in 1^st^ trimester


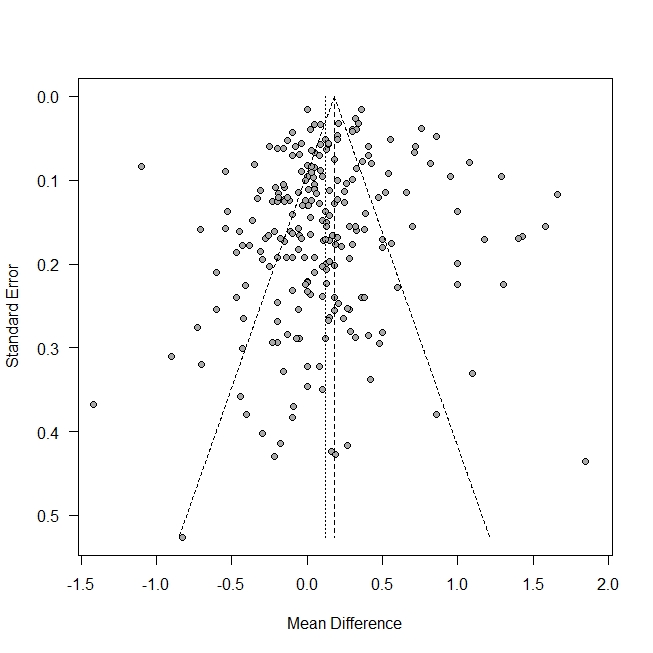


Figure S8. Funnel plot for LDL in 2^nd^ trimester


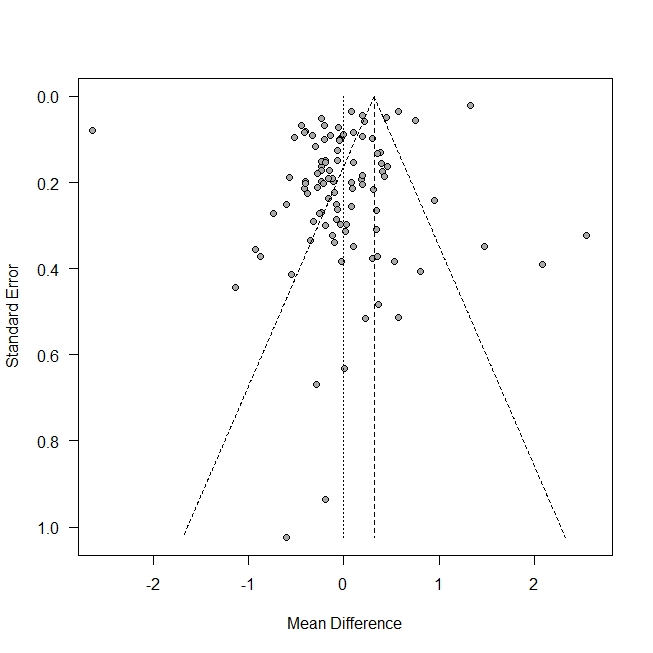


Figure S9. Funnel plot for LDL in 3^rd^ trimester


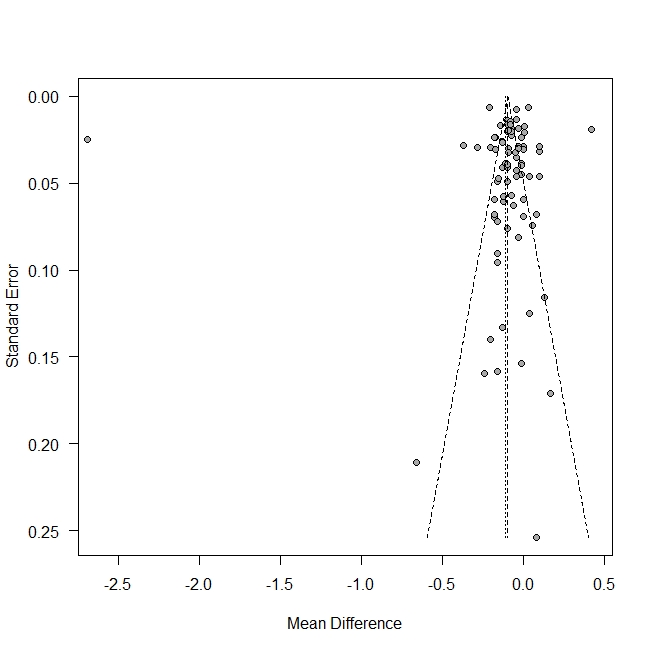


Figure S10. Funnel plot for HDL in 1^st^ trimester


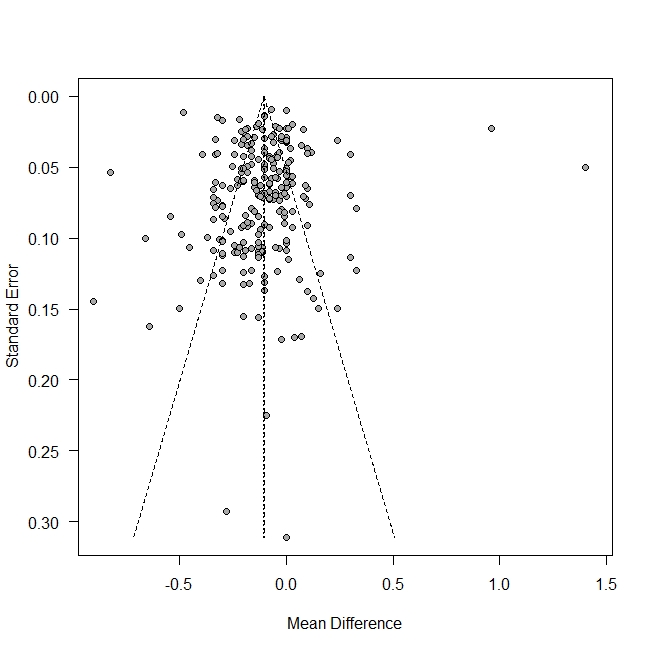


Figure S11. Funnel plot for HDL in 2^nd^ trimester


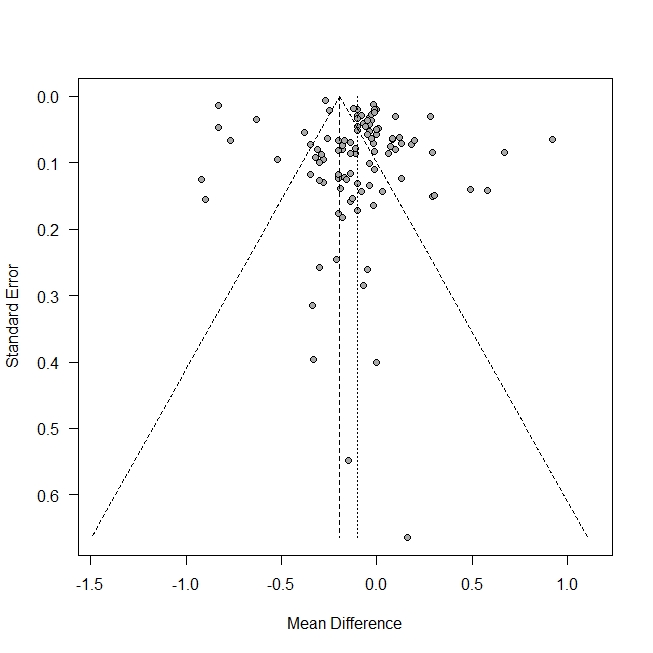


Figure S12. Funnel plot for HDL in 3^rd^ trimester


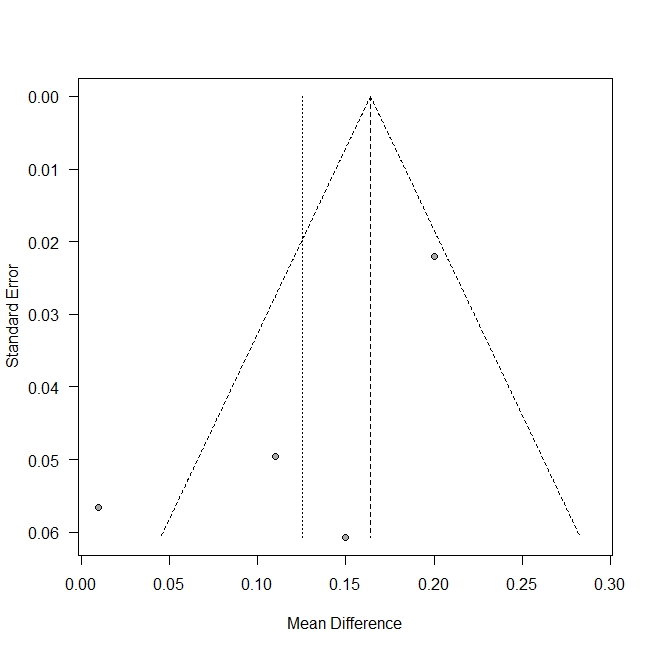


Figure S13. Funnel plot for VLDL in 1^st^ trimester


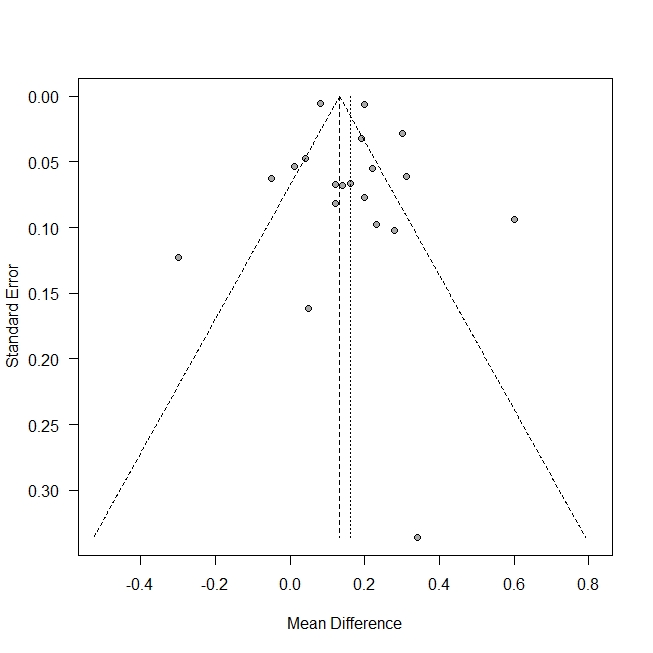


Figure S14. Funnel plot for VLDL in 2^nd^ trimester


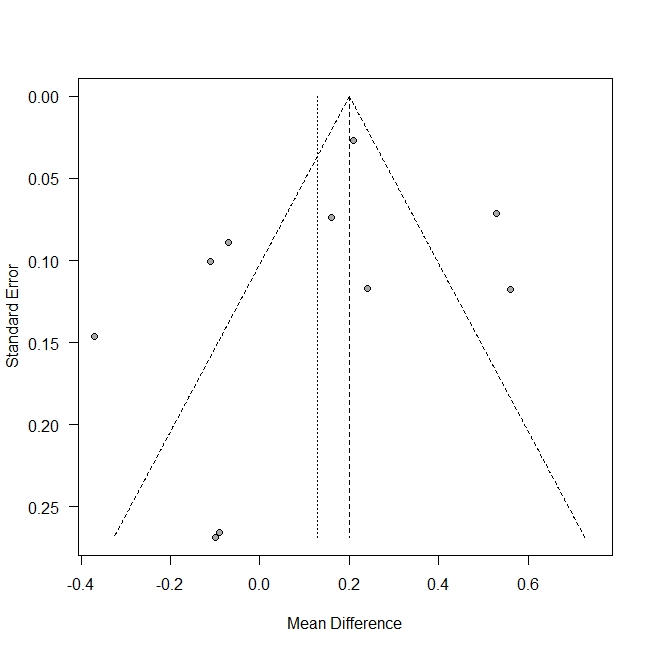


Figure S15. Funnel plot for VLDL in 3^rd^ trimester


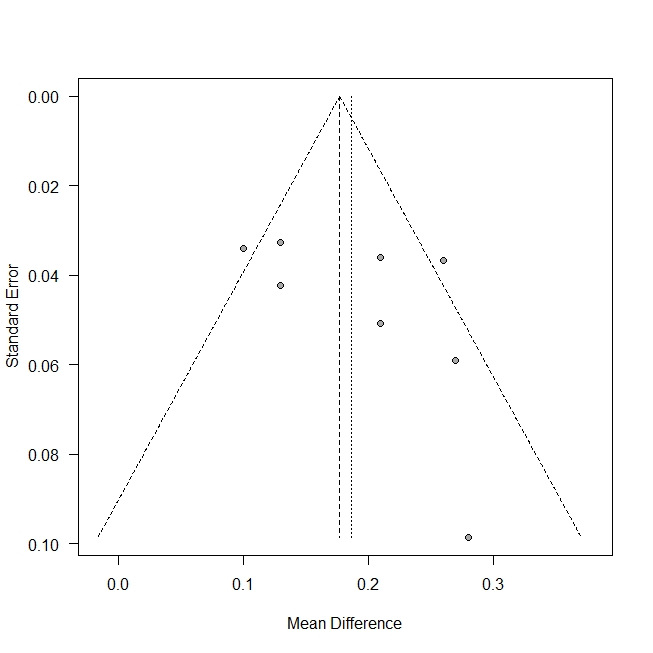


Figure S16. Funnel plot for Tg/HDL ratio in 1^st^ trimester


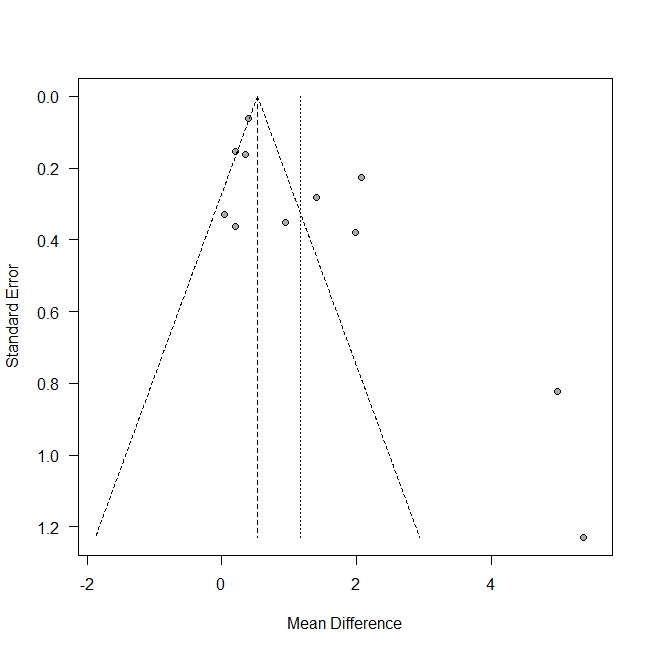


Figure S17. Funnel plot for Tg/HDL ratio in 2^nd^ trimester


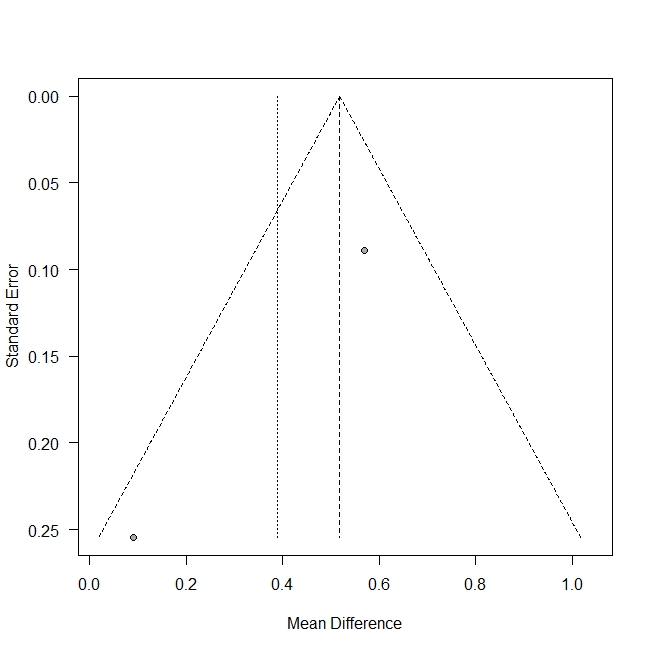


Figure S18. Funnel plot for Tg/HDL ratio in 3^rd^ trimester
