# Supplementary material for: Trimester-Specific Serum Lipid Profiles in Gestational Diabetes Mellitus: A Systematic Review, Meta-Analysis, and Meta-Regression
Source: Medicina (Kaunas). 2025 Jul 17;61(7):1290. doi: 10.3390/medicina61071290 (PMC12300116; doi:10.3390/medicina61071290)

Study	Experimental Total	Experimental Mean	Experimental SD	Control Total	Control Mean	Control SD	SMD	95%−CI	Weight (fixed)	Weight (random)
Montelongo, 1992	9	0.87	0.3000	12	0.68	0.3500	0.55	[−0.33; 1.44]	0.0%	0.5%
Nolan C, 1995	15	1.21	0.4100	108	1.03	0.3700	0.48	[−0.07; 1.02]	0.1%	0.9%
Nolan C, 1995	15	1.63	0.4800	108	1.16	0.4300	1.07	[0.51; 1.63]	0.1%	0.8%
Sánchez−Vera I, 2007	62	1.14	0.4600	45	0.80	0.3300	0.82	[0.42; 1.22]	0.2%	1.0%
Paradisi G, 2010	12	1.33	0.5200	38	1.27	0.6800	0.09	[−0.56; 0.74]	0.1%	0.7%
Savvidou M, 2010	124	1.49	0.6700	248	1.23	0.5000	0.46	[0.24; 0.68]	0.6%	1.2%
Makgoba M, 2011	90	1.38	0.6900	158	1.20	0.4500	0.33	[0.07; 0.59]	0.4%	1.2%
dos Santos−Weiss I, 2012	288	2.50	0.8900	288	1.10	0.3700	2.05	[1.85; 2.25]	0.7%	1.2%
Li G, 2015	379	1.61	0.8800	2166	1.26	0.6300	0.52	[0.41; 0.63]	2.5%	1.3%
Wang C, 2016	1062	1.38	2.1600	4203	1.17	0.9100	0.17	[0.10; 0.23]	6.7%	1.3%
Kumru P, 2016	38	1.64	0.7400	295	1.20	0.4500	0.89	[0.55; 1.24]	0.3%	1.1%
Yang X, 2017	19	3.53	0.2100	20	3.14	0.3500	1.32	[0.62; 2.01]	0.1%	0.7%
Yuan X, 2018	86	2.05	0.7600	273	1.85	0.4600	0.37	[0.12; 0.61]	0.5%	1.2%
Hou W, 2018	131	1.50	0.5200	138	1.30	0.4400	0.41	[0.17; 0.66]	0.5%	1.2%
Yuan X, 2018	86	2.05	0.7600	273	1.85	0.4600	0.37	[0.12; 0.61]	0.5%	1.2%
Bao W, 2018	107	1.78	1.5500	214	1.42	1.1700	0.27	[0.04; 0.51]	0.6%	1.2%
Madhu SV, 2019	45	1.54	0.9900	45	1.25	0.4700	0.37	[−0.05; 0.79]	0.2%	1.0%
Bawah AT, 2019	21	2.27	0.3200	291	1.77	0.8000	0.64	[0.20; 1.09]	0.2%	1.0%
Sweeting AN, 2018	248	0.99	0.3100	232	1.18	0.4500	−0.49	[−0.68; −0.31]	0.9%	1.2%
Pezeshki B, 2019	25	1.15	0.4500	301	1.07	0.3800	0.21	[−0.20; 0.62]	0.2%	1.0%
Correa, 2018	16	1.09	0.3600	80	1.55	0.5400	−0.89	[−1.44; −0.33]	0.1%	0.8%
Wang, 2019	300	1.08	0.5700	1283	0.91	0.5000	0.33	[0.20; 0.46]	1.9%	1.3%
Alyas S, 2019	58	4.17	0.3000	100	3.92	0.4000	0.68	[0.35; 1.01]	0.3%	1.1%
Zheng T, 2019	612	1.68	0.8000	4152	1.41	0.6400	0.41	[0.32; 0.49]	4.2%	1.3%
Jia H, 2019	136	2.18	1.0100	138	2.17	0.8800	0.01	[−0.23; 0.25]	0.5%	1.2%
Benhalima K, 2019	189	1.20	0.5200	1113	1.00	0.3000	0.59	[0.43; 0.74]	1.2%	1.2%
Ma S, 2020	98	1.57	0.6000	98	1.32	0.3800	0.50	[0.21; 0.78]	0.4%	1.1%
Mohammed Ali D, 2020	60	1.41	0.7000	30	1.14	0.9300	0.34	[−0.10; 0.78]	0.2%	1.0%
Sun T, 2020	258	2.86	1.1600	1154	2.51	0.9000	0.37	[0.23; 0.50]	1.6%	1.3%
Ye Y, 2020	2181	1.67	0.7900	2719	1.44	0.5900	0.34	[0.28; 0.39]	9.4%	1.3%
Contreras−Duarte S, 2020	69	1.44	0.7000	41	1.16	0.4500	0.45	[0.06; 0.84]	0.2%	1.0%
Jiang R, 2019	65	1.42	0.5700	366	1.23	0.4000	0.44	[0.18; 0.71]	0.4%	1.2%
Deischinger C. 2020	45	4.86	1.4500	48	1.73	0.9200	2.57	[2.02; 3.13]	0.1%	0.8%
Zhu H, 2020	581	1.20	0.6000	2368	1.00	0.4000	0.45	[0.36; 0.54]	3.6%	1.3%
Zhang X, 2020	274	1.15	0.8000	1111	0.96	0.5600	0.31	[0.18; 0.44]	1.7%	1.3%
McMichael L, 2021	34	2.31	1.0100	34	1.84	0.9200	0.48	[0.00; 0.96]	0.1%	0.9%
Tian M, 2021	51	1.80	0.6800	51	1.50	0.7200	0.43	[0.03; 0.82]	0.2%	1.0%
Wang X, 2021	607	1.56	0.7300	833	1.31	0.4700	0.42	[0.32; 0.53]	2.7%	1.3%
Wang Y, 2021	336	1.70	0.8000	672	1.50	0.6000	0.30	[0.17; 0.43]	1.8%	1.3%
Catov J, 2021	1102	1.40	1.1000	3285	1.30	0.9800	0.10	[0.03; 0.17]	6.5%	1.3%
Coussa R, 2021	34	2.00	1.8000	124	1.36	0.9100	0.55	[0.17; 0.93]	0.2%	1.0%
Hu Z, 2021	78	1.76	1.2300	30	1.13	0.4400	0.58	[0.15; 1.01]	0.2%	1.0%
Kotzaeridi G, 2021	239	1.47	0.5000	893	1.13	0.3000	0.97	[0.82; 1.11]	1.4%	1.3%
Abdualhay R, 2022	44	1.83	0.9300	45	1.46	0.6000	0.47	[0.05; 0.89]	0.2%	1.0%
An R, 2022	94	1.55	0.5400	572	1.33	0.5000	0.43	[0.22; 0.65]	0.6%	1.2%
Chen X, 2022	6	1.25	0.4700	27	1.33	0.3500	−0.21	[−1.10; 0.68]	0.0%	0.5%
Juchnicka I, 2022	24	1.08	0.4200	24	0.81	0.3300	0.70	[0.12; 1.29]	0.1%	0.8%
Song S, 2022	249	0.96	0.4200	879	0.85	0.3300	0.31	[0.17; 0.45]	1.5%	1.3%
Shen L, 2023	233	1.47	0.5600	1001	1.33	0.5300	0.26	[0.12; 0.40]	1.5%	1.3%
Zheng Y, 2022	142	1.91	0.5700	442	1.55	0.4700	0.72	[0.53; 0.92]	0.8%	1.2%
Sahoo D, 2022	20	1.99	0.1000	45	1.70	0.0500	4.15	[3.25; 5.06]	0.0%	0.5%
Tunc S, 2022	12	1.42	0.6000	88	1.52	0.4700	−0.20	[−0.81; 0.40]	0.1%	0.8%
Song S, 2022	145	0.96	0.4800	555	0.82	0.3900	0.34	[0.16; 0.52]	0.9%	1.2%
Aslan Çin N, 2022	46	1.81	0.7400	768	1.57	0.7900	0.30	[0.01; 0.60]	0.3%	1.1%
Zeljkovic A, 2022	15	1.67	0.8300	48	1.26	0.4000	0.76	[0.17; 1.36]	0.1%	0.8%
Zheng W, 2022	396	1.32	0.5600	2789	1.03	0.4200	0.66	[0.55; 0.77]	2.7%	1.3%
Wang F, 2023	59	1.18	0.6800	243	0.86	0.4000	0.68	[0.39; 0.97]	0.4%	1.1%
Cui, 2023	750	1.23	0.5200	4122	1.11	0.4000	0.29	[0.21; 0.36]	5.0%	1.3%
Liu, 2023	67	1.10	0.4300	446	0.90	0.3300	0.58	[0.32; 0.84]	0.5%	1.2%
Li, 2023	100	1.91	1.1000	218	1.50	0.5900	0.52	[0.28; 0.76]	0.5%	1.2%
Duo, 2022	300	1.00	0.4400	1043	0.83	0.3300	0.48	[0.35; 0.60]	1.8%	1.3%
Zou, 2023	40	2.32	0.7500	65	1.81	0.8000	0.65	[0.24; 1.05]	0.2%	1.0%
Wang, 2023	162	1.19	0.2100	48	0.87	0.1500	1.61	[1.25; 1.97]	0.2%	1.1%
Gao, 2023	37	4.56	2.1200	553	2.98	1.0800	1.35	[1.01; 1.69]	0.3%	1.1%
Mustaniemi, 2023	1040	1.70	0.8700	958	1.36	0.5300	0.47	[0.38; 0.56]	3.8%	1.3%
Cui, 2023	150	2.46	0.3200	150	1.39	0.4100	2.90	[2.58; 3.23]	0.3%	1.1%
Duo, 2023	272	1.00	0.4400	1017	0.80	0.3700	0.52	[0.38; 0.65]	1.7%	1.3%
Ortega−Montiel, 2024	294	1.60	0.6000	1762	1.50	0.6000	0.17	[0.04; 0.29]	2.0%	1.3%
Ma, 2024	201	1.90	0.8100	872	1.51	0.6000	0.60	[0.45; 0.76]	1.3%	1.2%
Zhao, 2024	261	1.15	0.8000	1327	0.96	0.5600	0.31	[0.18; 0.45]	1.7%	1.3%
Rajeevan, 2024	29	1.31	0.7900	143	1.19	0.3900	0.25	[−0.15; 0.65]	0.2%	1.0%
Niu, 2024	519	1.85	0.8500	1281	1.47	0.6100	0.55	[0.45; 0.66]	2.8%	1.3%
Ma, 2024	103	1.92	1.1000	225	1.51	0.5900	0.52	[0.28; 0.76]	0.5%	1.2%
Hou W, 2016	268	1.90	1.1000	474	1.70	1.0000	0.19	[0.04; 0.34]	1.3%	1.2%
Houde AA, 2013	26	1.24	0.5000	74	1.07	0.4800	0.35	[−0.10; 0.80]	0.2%	1.0%
Pazhohan A, 2017	176	2.24	1.1900	778	1.85	0.9200	0.40	[0.23; 0.56]	1.1%	1.2%
Ren Z, 2020	51	2.12	0.6100	48	1.32	0.5600	1.35	[0.92; 1.79]	0.2%	1.0%
Ruchat SM, 2013	30	1.36	0.5100	14	1.19	0.4500	0.34	[−0.30; 0.98]	0.1%	0.8%
Bawah AT, 2019	70	2.73	0.7100	70	1.51	0.6900	1.73	[1.34; 2.12]	0.2%	1.0%
Jin C, 2021	135	1.20	0.3700	135	1.00	0.3000	0.59	[0.35; 0.84]	0.5%	1.2%
Li J, 2021	80	1.49	0.7900	317	1.29	0.6500	0.29	[0.05; 0.54]	0.5%	1.2%
Lin Q, 2023	197	1.69	0.5600	209	1.43	0.6900	0.41	[0.22; 0.61]	0.8%	1.2%
Lu L, 2022	74	2.73	0.2100	414	1.62	0.1400	7.26	[6.74; 7.78]	0.1%	0.9%
Meyer B, 2023	43	2.60	0.8900	26	1.71	0.6900	1.07	[0.55; 1.59]	0.1%	0.9%
Song S, 2021	239	0.95	0.3200	843	0.83	0.2400	0.46	[0.32; 0.61]	1.4%	1.3%
Sun J, 2021	144	1.11	0.7700	600	0.98	0.5800	0.21	[0.03; 0.39]	0.9%	1.2%
Wang N, 2021	532	1.76	0.7700	516	1.56	0.6100	0.29	[0.17; 0.41]	2.0%	1.3%
Wang X, 2022	49	1.36	0.6100	50	1.10	0.4100	0.50	[0.10; 0.90]	0.2%	1.0%
Wang W, 2023	256	2.06	0.9100	2272	1.72	0.6900	0.48	[0.35; 0.61]	1.8%	1.3%
Wani K, 2020	123	1.70	0.8000	375	1.40	0.6000	0.46	[0.25; 0.66]	0.7%	1.2%
Zhao X, 2023	231	1.13	0.4700	1091	0.95	0.5800	0.32	[0.18; 0.46]	1.5%	1.3%
**Fixed effect model**	**18819**			**60241**			**0.41**	**[0.39; 0.43]**	**100.0%**	**−−**
**Random effects model**							**0.61**	**[0.52; 0.69]**	**−−**	**100.0%**

Heterogeneity: $I^2 = 95\%$, $\tau^2 = 0.1480$, $p = 0$

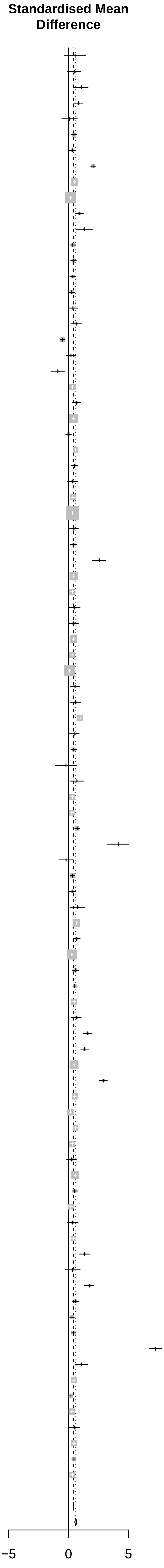

Supplement: Supplementary file 1 [file medicina-61-01290-s001.zip › Figure S19 TG 1st trimester.pdf]
