# Supplementary figures and images for: Trimester-Specific Serum Lipid Profiles in Gestational Diabetes Mellitus: A Systematic Review, Meta-Analysis, and Meta-Regression
Source: Medicina (Kaunas). 2025 Jul 17;61(7):1290. doi: 10.3390/medicina61071290 (PMC12300116; doi:10.3390/medicina61071290)

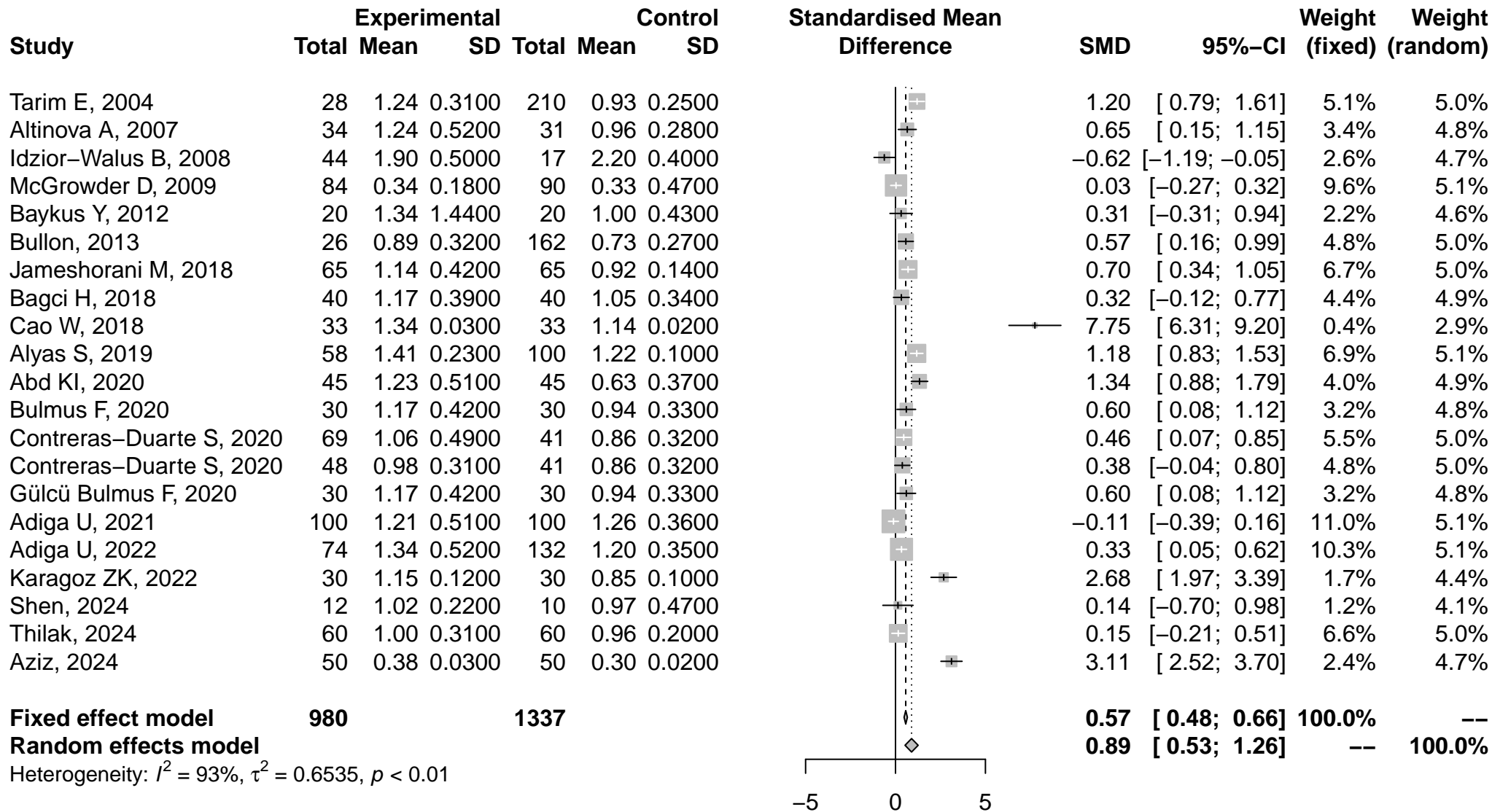

Supplement: Supplementary file 1 [file medicina-61-01290-s001.zip › Figure S32 VLDL 2nd trimester.pdf]

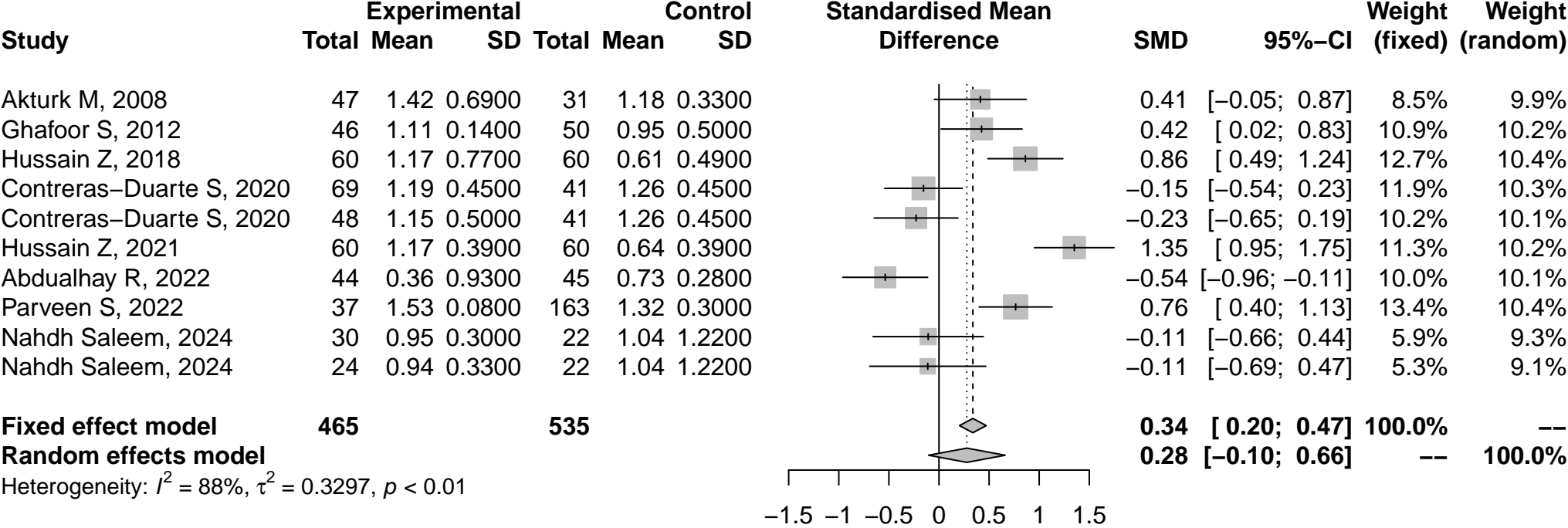

Supplement: Supplementary file 1 [file medicina-61-01290-s001.zip › Figure S33 VLDL 3rd trimester.pdf]

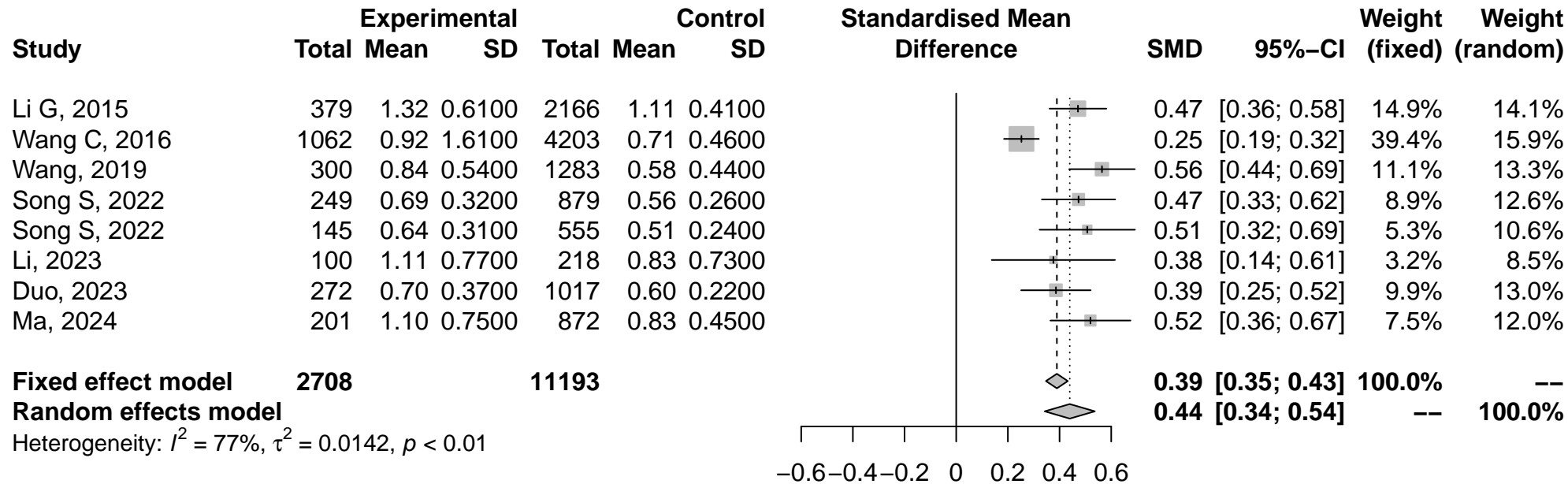

Supplement: Supplementary file 1 [file medicina-61-01290-s001.zip › Figure S34 TgHDL ratio 1st trimester.pdf]

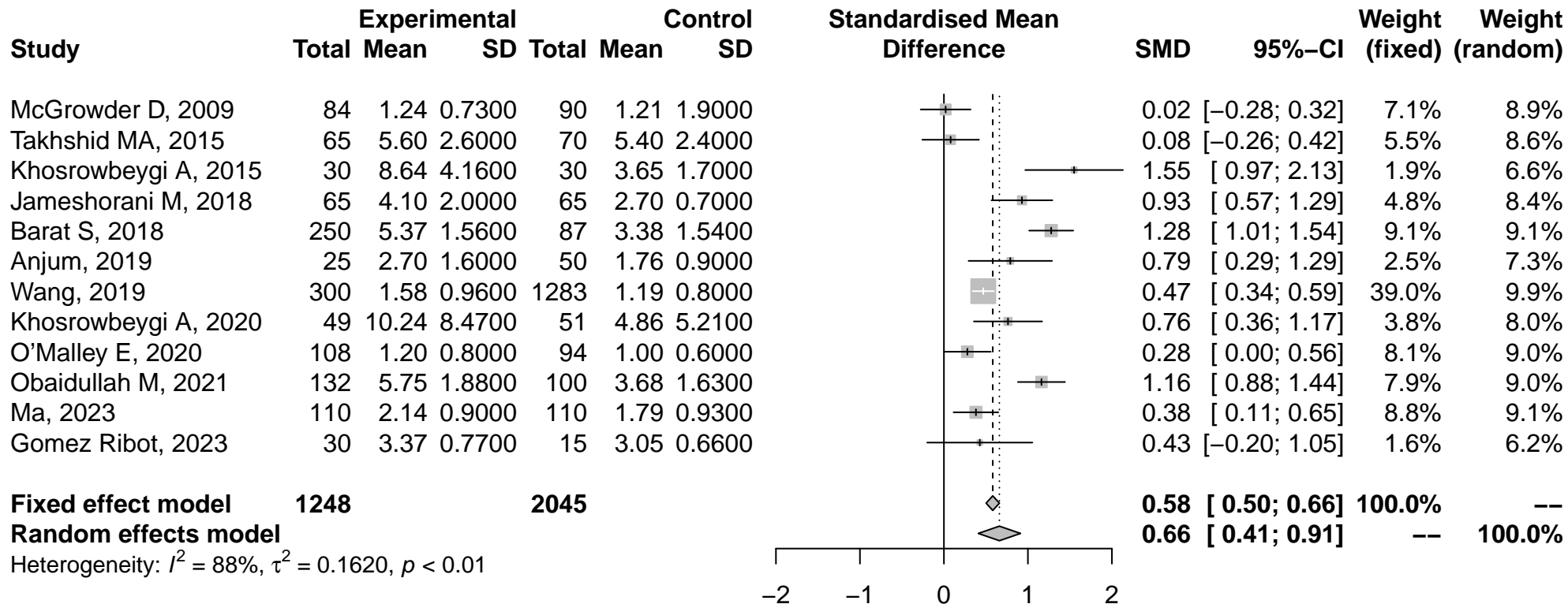

Supplement: Supplementary file 1 [file medicina-61-01290-s001.zip › Figure S35 TgHDL ratio 2nd trimester.pdf]

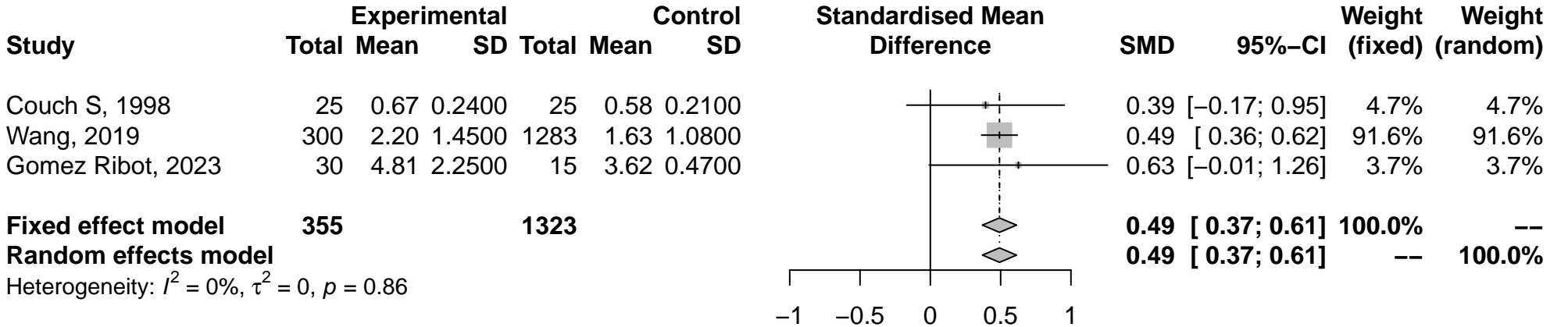

Supplement: Supplementary file 1 [file medicina-61-01290-s001.zip › Figure S36 TgHDL ratio 3rd trimester.pdf]
